# Collective problem decomposition improves the wisdom of deliberative crowds

**DOI:** 10.1038/s41467-026-76365-y

**Published:** 2026-08-05

**Authors:** Federico Barrera-Lemarchand, Victoria Lescano-Charreau, Julieta Ruiz, Nuria Cáceres, Facundo Carrillo, Mariano Sigman, Joaquin Navajas

**Affiliations:** 1 Laboratorio de Neurociencia, Escuela de Negocios, Universidad Torcuato Di Tella, Buenos Aires, Argentina; 2 Consejo Nacional de Investigaciones Científicas y Técnicas (CONICET), Buenos Aires, Argentina; 3 Facultad de Ciencias Exactas y Naturales, Universidad de Buenos Aires, Buenos Aires, Argentina; 4 Laboratorio Interdisciplinario del Tiempo y la Experiencia (LITERA), Universidad de San Andrés, Buenos Aires, Argentina; 5 Instituto de Investigación en Ciencias de la Computación (ICC), CONICET-UBA Buenos Aires, Argentina

**Keywords:** Decision making, Human behaviour

## Abstract

Understanding when and why social interaction improves human judgment is a central question in the behavioural sciences. We examine whether collective accuracy improves when groups break complex estimation problems into simpler components and generate approximate intermediate estimates, a reasoning strategy we call Collective Fermi Estimation. Across three studies analysing 1140 online group deliberations in text chatrooms we study its causal effects and linguistic signatures. In Study 1 (N = 500), spontaneous use of problem decomposition predicts lower collective estimation error. Study 2 (N = 240) provides causal evidence: groups instructed to apply problem decomposition outperform groups instructed to combine their initial guesses. Study 3 (N = 160) shows that the benefits are larger when the strategy is applied collectively rather than individually. Altogether, we show that problem decomposition constitutes a key mechanism behind the wisdom of deliberative crowds and we provide tools to detect and promote it.

## Abstract (web summary)

Consensus estimates from small groups are more accurate than pooled individual estimates, yet what drives this is unclear. Here, the authors show that breaking down the problem and reasoning together is key, surpassing aggregation of initial guesses.

## Introduction

The aggregation of many lay estimates often outperforms individual expert judgments^1,2^. This phenomenon, popularly known as the “wisdom of crowds”^3^, has been applied to a wide range of problems, including predicting financial markets^4^, forecasting geopolitical events^5^, improving medical diagnoses^6^, and even decoding the smell of molecules^7^. Given the ubiquity of this effect and its broad applications across policymaking^8^, medicine^9^, and finance^10^, understanding why collective judgments sometimes enhance decision accuracy and at other times fail to do so has become a central challenge for the social and behavioral sciences^11–13^.

Previous research has examined the impact of social influence on the wisdom of crowds, yielding mixed results. Some studies suggest that it can lead to herding and reduce accuracy^14–16^, whereas others show that social interaction can improve collective estimates^5,17–19^. For instance, averaging the consensus estimates reached by small groups has been found to significantly outperform the wisdom of large, independent crowds^12^. Although this result has been replicated across multiple contexts^20–22^, the procedural mechanisms that determine when group consensus enhances or undermines collective accuracy remain poorly understood.

Based on theoretical considerations^23^, groups can follow two distinct procedures to reach a collective estimate. One approach involves combining the initial private signals, for example, by taking the mean, the median, a confidence-weighted mean, or any other form of aggregation. A fundamentally different approach uses the deliberation process to develop a shared line of reasoning and generate a new estimate that is not merely a function of the initial individual judgments.

To illustrate these two strategies, consider a group of four individuals who independently estimate the height of the Eiffel Tower as 100, 200, 500, and 800 m, and then come together to reach a shared judgment. The group might decide to discard the extreme values and average the two middle estimates, arriving at 350 m, just 26 m off the correct height. They could also average all four values, weight them by confidence, or use the median. What all these procedures have in common is that the final estimate is derived directly from the original individual judgments. Therefore, if groups follow this approach, they must exchange their initial estimates during deliberation.

Alternatively, the group might engage in a joint reasoning process to solve the problem, setting aside their initial estimates. For instance, they could discuss the structural features of the Eiffel Tower, reasoning that it resembles a 100-story building, with each floor about 3.5 m high, again yielding an estimate of roughly 350 m. Unlike the previous approach, it does not require sharing initial estimates. Because this strategy extends a well-established problem-solving technique for back-of-the-envelope calculations (attributed to Enrico Fermi) to a group setting^24–26^, we refer to it as the Collective Fermi Estimation strategy.

Previous works have shown that consensus estimates produced by small groups can outperform the classical wisdom of crowds, suggesting that groups do more than merely averaging their initial judgments^12,20,21^. However, because the content of group discussions was not available in those works, it remained unclear whether groups relied on a mixture of aggregation rules or on a qualitatively different reasoning procedure. The present research was designed to identify the strategies that groups use to reach consensus and to determine which of these strategies are most effective for improving collective accuracy.

Fermi problems are estimation problems that require approximate reasoning in situations where exact information is unavailable. Although the “Fermi Method” is widely referenced across fields ranging from mathematics education^27^ to psychology^28^, its definition is often described informally. Previous work consistently characterizes the method as a structured estimation procedure in which a complex quantity is approximated by decomposing it into simpler components that can be estimated separately^27–31^.

To clarify this concept, we reviewed prior descriptions of the Fermi method in the literature (Table S1). Despite differences in terminology, these definitions converge on two core reasoning steps. First, the target quantity is decomposed into simpler subcomponents that can be estimated independently. Second, approximate values are proposed for those components using rough approximations or “guesstimates”, which are then recombined to produce a final estimate.

Based on this convergence in prior work, we adopt the following operational definition throughout this paper: “*The Fermi Method consists of breaking the problem into simpler components, making rough estimations for each component, and calculating the final answer based on those estimations*.” When this procedure is implemented through group deliberation, we call it Collective Fermi Estimation.

Building on prior evidence that collective reasoning can enhance performance beyond individual reasoning^32–34^, we hypothesized that groups engaging in the Collective Fermi Estimation procedure would achieve lower error. To test this hypothesis, we developed a variety of methods to quantify the extent to which deliberation relies on this strategy, combining human ratings with natural language processing of group discussions.

Study 1 uses this measure to examine whether greater reliance on Collective Fermi Estimation predicts higher accuracy. Study 2 introduces an experimental manipulation, providing some groups with explicit instructions to apply problem decomposition and comparing their performance to that of groups instructed to aggregate their initial values. Study 3 compares individual and collective implementations of the same reasoning procedure, showing that the advantage emerges when the strategy operates at the collective level rather than in isolation.

## Results

### Study 1: Replication of Navajas et al.^12^ in chatrooms

**Fig. 1:**
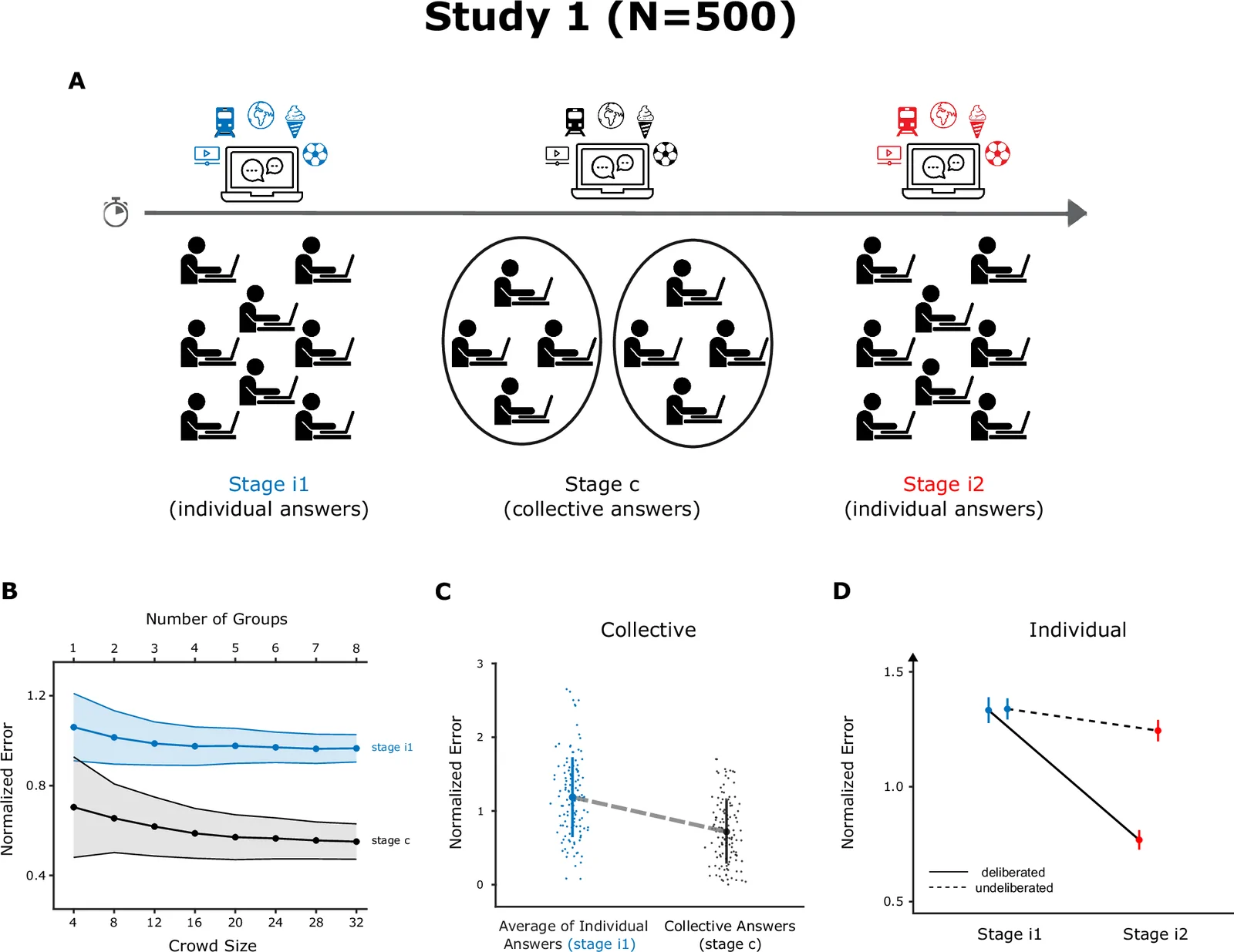
Empirical results for Study 1. **A** The study had three stages. On Stage i1, participants answered eight general-knowledge questions individually. On Stage c, participants were divided into groups of four, and were asked half of the questions from the previous stage. Stage i3 followed the same procedure as Stage i1. Additional icons made by Freepik and Good Ware from www.flaticon.com. **B** Normalized error of the average of *c* individual answers (blue line for stage i1), and normalized error of the average of *c*/4 collective estimates (black line, stage c), where *c* denotes the crowd size. Error bars represent the standard deviation across 500 random subsamples per crowd size. **C** Average normalized error and distributions of normalized errors of the individual answers (blue), and the collective answers (black), computed from *n* = 125 biologically independent groups. The error bars correspond to the standard deviation of the means. **D** Mean normalized error for the individual stages, for both deliberated (*n* = 1291 participant-question observations from biologically independent participants) and undeliberated (*n* = 1812 participant-question observations from biologically independent participants) questions. The error bars correspond to 95% confidence intervals.

500 participants (288 female, mean age 25.4 years, s.d. 7.7 years), organized into 125 groups of four individuals, participated in Study 1. Participants were incentivized to provide accurate answers (for details, see Methods). The study, which was exploratory in nature, consisted of three stages (Fig. 1A). During the first stage (stage i1), participants were asked individually a series of eight general-knowledge estimation questions (Table S2). In the second stage (stage c), they were divided into groups of four, and they were asked a random subset of four of the previous questions. They were instructed to deliberate these questions in a virtual chatroom and asked to try to reach a consensus. All conversations were recorded. In the last stage (stage i2), which followed exactly the same procedure as the first stage, participants could provide new estimates and confidence values.

Despite methodological differences with the original study, particularly with respect to group size (four vs. five) and modality (face-to-face vs. chatroom conversations), we replicated the observation that averaging collective estimates led to lower estimation error than averaging initial independent values (Fig. 1B, see “Methods” for details). We observed this pattern for all values displayed in the *x*-axis of Fig. 1B: For example, the estimation error of aggregating 8 randomly-chosen consensus estimates was lower than the error obtained by averaging 32 randomly-chosen individual values (Mann–Whitney *U* test: *z* = 19.3, *p* < 0.001; effect size: Cohen’s *d* = 6.22, 95% CI [5.79, 6.63]). This finding implies that collective estimates were more accurate than what we would have observed if groups had reached consensus by computing the mean of their initial values (Fig. 1C, normalized error of within-group averages: 1.18 ± 0.05, normalized error of consensus estimates: 0.72 ± 0.04, Wilcoxon signed-rank test: *z* = 7.3, *p* < 0.001; effect size: paired Cohen’s *d* = 0.81, 95% CI [0.61, 1.01]).

Lastly, comparing participants’ estimates from the first and third stages, separating questions that were deliberated in groups from those that were not, reveals a greater reduction in error for the deliberated questions (Fig. 1D). Although participants showed a modest improvement in accuracy for undeliberated questions, the improvement was larger for the deliberated questions (generalized linear mixed-effects model, see Methods for details: *β* = −0.48 [−0.53, −0.43], SE = 0.03, *t*(2787) = −17.9, *p* < 0.001, *R*^2^ = 0.148).

In principle, one might assume that collective deliberation was dominated by individuals with higher accuracy or confidence, and that this alone could account for the improved accuracy of consensus estimates compared to simple averages. However, the data indicate that contributions within groups were approximately uniform. When we rank participants by individual error (Fig. S1A) or by confidence (Fig. S1B), the proportion of interventions made by each of the four group members is statistically indistinguishable from an equal share of 25% (two one-sided tests for equivalence, *p* < 0.001 for all comparisons; see Table S3 for details). This suggests that the accuracy gains were not simply driven by a few dominant individuals but rather emerged from broadly distributed contributions within the group.

### Collective Fermi estimation is associated with lower error

We then turned to the central research question of this study: What strategy do groups use to reach consensus? Do they share and combine their initial estimates, or do they recompute the value through problem decomposition? To investigate this, we trained ten individuals (8 female; mean age = 22.8 years, s.d. = 5.4) to rate each conversation along two dimensions using 11-point Likert scales (see “Methods” for rating instructions). The first rating assessed the extent to which group members shared and combined their initial judgments, a strategy we call “Sharing Numbers” (or simply “Numbers”). The second rating captured the extent to which participants generated new estimates through problem decomposition, a strategy we refer to as “Collective Fermi Estimation” (or simply “Fermi”).

**Fig. 2:**
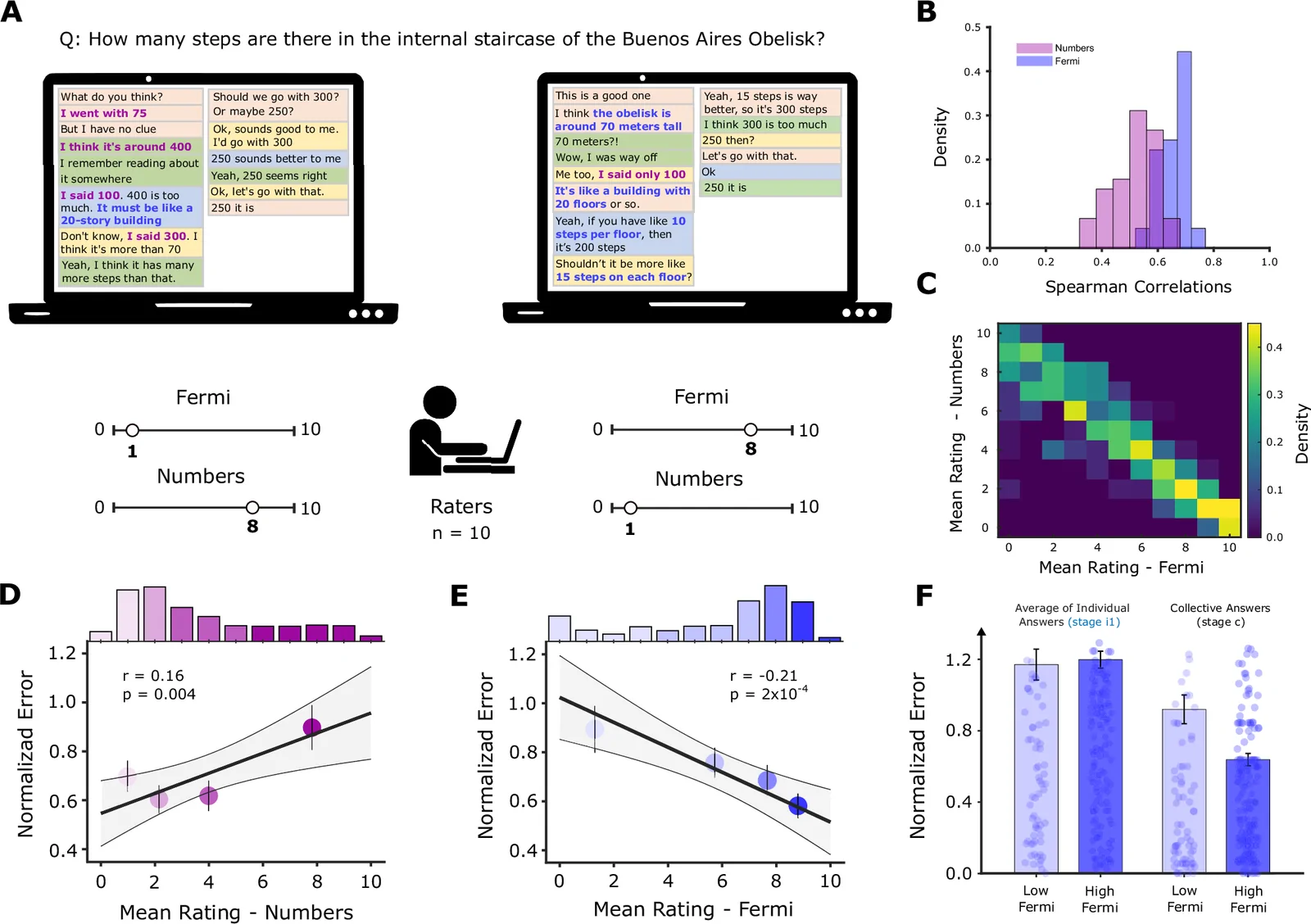
Analysis of conversations. **A** Examples of conversations rated as high in either the Numbers strategy (sharing individual estimates, left panel) or the Fermi strategy (collective approximation, right panel). These examples are illustrative and do not pretend to imply that these particular values (1 and 8) are uniquely correct. Ten raters classified each conversation along these dimensions. Additional icons made by Freepik and Maxim Basinski Premium from www.flaticon.com. **B** Distribution of pairwise Spearman correlations between raters for each strategy. **C** Density plot of mean Numbers and Fermi ratings, showing a strong negative correlation between strategies. **D**, **E** Normalized error as a function of average strategy ratings. Dots represent quartiles in the distribution of mean ratings, which are displayed above each plot. Lines depict the best linear fits, and shades represent 95% confidence intervals. Statistical test reported in Results: two-sided Pearson correlation. **F** Normalized error before and after deliberation for conversations categorized as low Fermi (or high Numbers; *n* = 101 biologically independent conversations) or high Fermi (or low Numbers; *n* = 224 biologically independent conversations) based on which strategy received the higher average rating. Error bars represent the standard error of the mean.

Figure 2A illustrates two real examples: one conversation in which participants combined their initial estimates to reach consensus (left panel), and another in which participants decomposed the problem into intermediate quantities and proposed approximate values for those quantities (right panel). These examples illustrate how different conversational strategies can lead raters to assign different Numbers and Fermi scores. In particular, conversations dominated by the exchange and aggregation of initial estimates would typically receive higher Numbers ratings, whereas conversations involving problem decomposition and rough approximations would tend to receive higher Fermi ratings. These examples are intended only as illustrative cases showing how raters may interpret the conversational strategies present in the discussion, rather than implying that any specific numerical rating is uniquely correct for these conversations.

We observed moderate-to-strong pairwise Spearman correlations between raters, ranging from 0.35 to 0.76 (Fig. 2B; inter-rater correlation for Numbers: 0.518 ± 0.002, *p* < 0.001; for Fermi: 0.661 ± 0.001, *p* < 0.001), indicating a high level of consistency across them. Given this reliability, we used the average rating across raters as a summary measure of the extent to which each strategy was employed in each conversation. These averaged ratings revealed a strong negative correlation between Numbers and Fermi scores (Fig. 2C; Pearson *r* = −0.92, 95% CI [−0.94, −0.91], *p* < 0.001), suggesting that the two strategies were typically used in a mutually exclusive manner.

We conducted two control analyses to increase confidence in human ratings. First, we found that the mean Numbers rating was positively correlated with the actual proportion of messages in which participants shared a number matching one of the initial individual estimates (Pearson *r* = 0.32, 95% CI [0.23, 0.40], *p* < 0.001), and that this behavioral measure also correlated negatively with Fermi ratings (Pearson *r* = −0.30, 95% CI [−0.38, −0.21], *p* < 0.001).

Second, we conducted an additional annotation exercise designed to capture a broader form of deliberation. A separate group of six raters (5 female; mean age = 25.8 years, s.d. = 5.0) evaluated the conversations using more general instructions, rating the extent to which participants exchanged reasons to justify the group’s consensus value. This instruction was motivated by the distinction commonly made in the collective intelligence literature between number-based aggregation and deliberation involving the exchange of reasons (e.g., see Landemore and Page^23^). Because the Fermi method is itself a reasoning procedure, we expected that conversations exhibiting stronger Fermi-style reasoning would also receive higher reasoning ratings.

Consistent with this expectation, these broader reasoning ratings were correlated with the original Fermi ratings (Pearson *r* = 0.84, 95% CI [0.81, 0.87], *p* < 0.001), and the main results reported in Fig. 2 remain unchanged under this alternative annotation scheme (Fig. S2). Importantly, these reasoning ratings were not intended as a mechanistic operationalization of the Fermi method itself, but to increase confidence in the robustness of the annotations: because Fermi-style estimation is one form of reason-based deliberation, similar results under this broader coding scheme indicate that the findings do not hinge on a narrowly framed annotation instruction.

We then examined whether and how the application of each strategy was associated with lower or higher estimation error. We found that conversations with higher Numbers ratings produced consensus estimates with higher estimation error (Fig. 2D, Pearson correlation: *r* = 0.16, 95% CI [0.05, 0.26], *p* = 0.004) and that conversations scoring more highly on the Fermi strategy led to collective estimates which were less erroneous (Fig. 2E, Pearson correlation: *r* = −0.20, 95% CI [−0.31, −0.10], *p* < 0.001).

One might assume that groups with higher Fermi scores were more accurate at the collective stage simply because they were already more accurate at the individual stage. However, our data rule out this explanation. Conversations in which the Fermi score exceeded the Numbers score did not originate from more accurate individuals than those where the Numbers score was higher (Fig. 2F; Cohen’s *d* = 0.018; BF_01_ = 7.5, strong evidence for the null hypothesis; posterior median *δ* = 0.02, 95% CrI [−0.21, 0.25]; sensitivity: BF_01_ = 10.5 with *r* = 1, BF_01_ = 14.7 with *r* = $\sqrt{2}$). The difference in accuracy between these groups only emerged after deliberation (Fig. 2F; Cohen’s *d* = 0.37; BF_01_ = 0.08, strong evidence for the alternative hypothesis; posterior median |δ| = 0.36, 95% CrI [0.13, 0.59]; sensitivity: BF_01_ = 0.10 with *r* = 1, BF_01_ = 0.13 with *r* = $\sqrt{2}$).

Another potential concern is that Fermi-based discussions may simply take longer than conversations focused on sharing numbers, and this alone may drive performance. However, while conversations with higher Fermi ratings indeed tended to be longer (Pearson *r* = 0.42, 95% CI [0.33, 0.49], *p* < 0.001), conversation length itself did not significantly predict estimation error (Pearson *r* = −0.07, 95% CI [−0.17, 0.04], *p* = 0.228), with a Bayesian analysis providing moderate evidence for the null hypothesis (BF_01_ = 6.59, posterior median *ρ* = −0.07, 95% CrI [−0.18, 0.04], qualitatively robust across alternative prior scales, with BF_01_ between 5.37 and 8.17). This suggests that the observed performance gains are not simply driven by groups spending more time discussing the problems.

### Predicting accuracy through automated language analysis

Because identifying groups that implemented the Collective Fermi Estimation strategy relies on human ratings, the process remains costly, time-consuming, and difficult to scale. To address this, we developed a computational method to automatically identify high-performing conversations. The objective of this analysis is not to provide a mechanistic test of Fermi reasoning, but rather to examine whether a simple and low-cost linguistic proxy could identify conversational patterns associated with higher Fermi ratings and lower collective error. This motivation is relevant because extracting the wisdom of crowds from deliberative settings has numerous potential applications (e.g., prediction markets, financial forecasting, health decision-making, etc.) and scalable methods for identifying high-performing conversations could therefore be practically valuable.

**Fig. 3:**
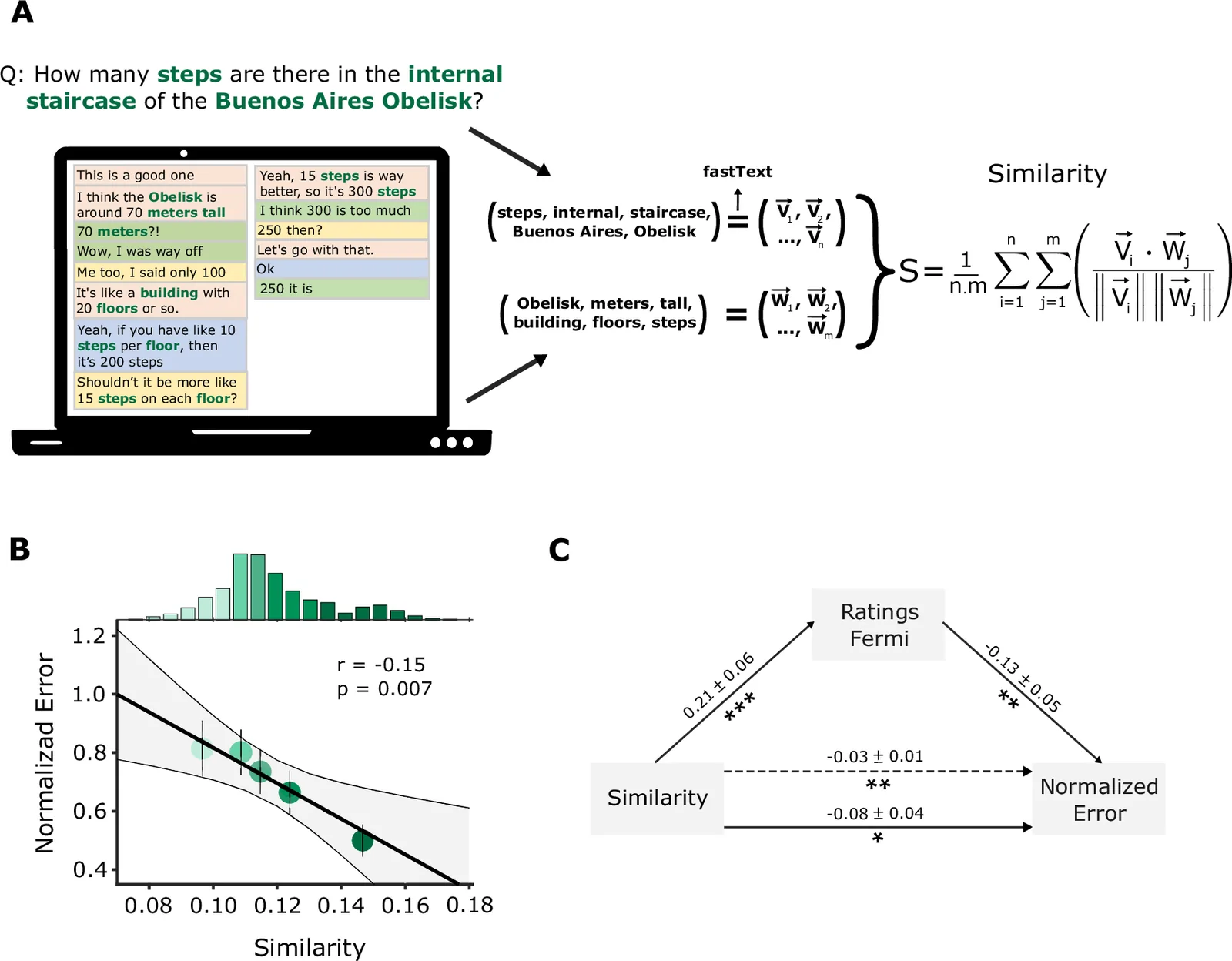
Analysis of text. **A** For each conversation, we computed the similarity between all words in the conversation and all words in the question (excluding stop words and short words). Bags of words were created for both the question and the conversation, and FastText was used to obtain word embeddings. We then calculated the average similarity between all vectors from the two sets. Laptop icon made by Maxim Basinski Premium from www.flaticon.com. **B** Normalized error as a function of similarity. Quintiles of the similarity distribution are shown (dots represent the mean normalized error within each similarity quintile) along with the best linear fit, 95% confidence intervals, and the two-sided Pearson correlation coefficient (*r*) with its corresponding *p* value. Bars above the graph indicate the full distribution of similarity values. **C** Mediation analysis testing whether the relationship between similarity and normalized error is mediated by Fermi ratings. Asterisks denote significance from bootstrap-based two-sided tests on each path coefficient: *p < 0.05, **p < 0.01, ***p < 0.001. Exact *p* values are reported in Results.

The underlying intuition of this approach is simple: If groups use the deliberation stage to generate a new collective estimate, they are likely to use words that are semantically related to those in the estimation question. For example, consider a group tasked with estimating the number of steps in the internal staircase of the Buenos Aires Obelisk (Fig. 3A). A group decomposing the problem might compare the Obelisk to a 20-story building, estimate that a typical staircase has around 15 steps per floor, and conclude that the Obelisk should have approximately 250 steps, a solution which is reasonably close to the actual value of 206. Such a conversation would naturally include words that either appear in the question itself (e.g., “staircase,” “steps”) or are semantically related (e.g., “building,” “floor”).

To quantify this intuition, we used pre-trained word embeddings^35^, specifically FastText embeddings trained in Spanish^36^. Word embeddings (vector representations of words) capture semantic relationships, such that semantically related words are represented by similar vectors. We computed the similarity between all words in the question and all words in the corresponding conversation, excluding stop words and short words. This yielded a distribution of similarity values, from which we extracted the mean to create a “Similarity index” (right panel in Fig. 3A).

We found that this Similarity index was positively correlated with Fermi ratings (Pearson *r* = 0.21, 95% CI [0.12, 0.30], *p* < 0.001) and negatively correlated with Numbers ratings (Pearson *r* = –0.16, 95% CI [−0.25, −0.07], *p* < 0.001). Moreover, the Similarity index was negatively associated with estimation error (Fig. 3B; Pearson *r* = –0.15, 95% CI [−0.25, −0.04], *p* = 0.006), indicating that conversations more semantically related to the estimation problem tended to produce more accurate collective estimates. A mediation analysis further showed that this relationship was partially explained by Fermi ratings (Fig. 3C; total effect = −0.11 ± 0.03, *p* = 0.006; direct effect = −0.08 ± 0.04, *p* = 0.021; indirect effect = −0.03 ± 0.01, *p* = 0.009).

To assess whether simpler alternatives could reproduce the findings obtained with the Similarity index, we also examined two baseline methods: the total number of words in each conversation, and the number of words from the corresponding question (excluding stop words and short words) that appeared in the conversation. The former could reflect the complexity of the group’s reasoning process, while the latter offers a crude approximation of semantic overlap. However, neither measure was significantly associated with normalized group error (Pearson correlations: *r* = −0.067, 95% CI [−0.18, 0.03], *p* = 0.228; and *r* = −0.031, 95% CI [−0.19, 0.03], *p* = 0.581, respectively). These results suggest that simply counting words or matching lexical items from the question is insufficient to capture the underlying reasoning dynamics. Instead, the semantic similarity captured by word embeddings appears necessary to detect high-performing groups.

The results presented so far provide evidence for an association between engaging in problem decomposition and producing collective estimates with higher estimation accuracy. To study if the implementation of this strategy, as opposed to just combining initial estimates, causally drives the observed reduction in collective error, we performed an experiment.

### Study 2: Collective Fermi estimation causally increases collective accuracy

A total of 240 participants (148 female, mean age 30.6 years, s.d. 9.8 years), organized in 60 groups of four individuals, participated in Study 2. Participants had monetary incentives to estimate these variables as accurately as possible (for details, see “Methods”). The protocol, hypotheses, and analysis plans were preregistered at https://aspredicted.org/TL2_Q1Q.

**Fig. 4:**
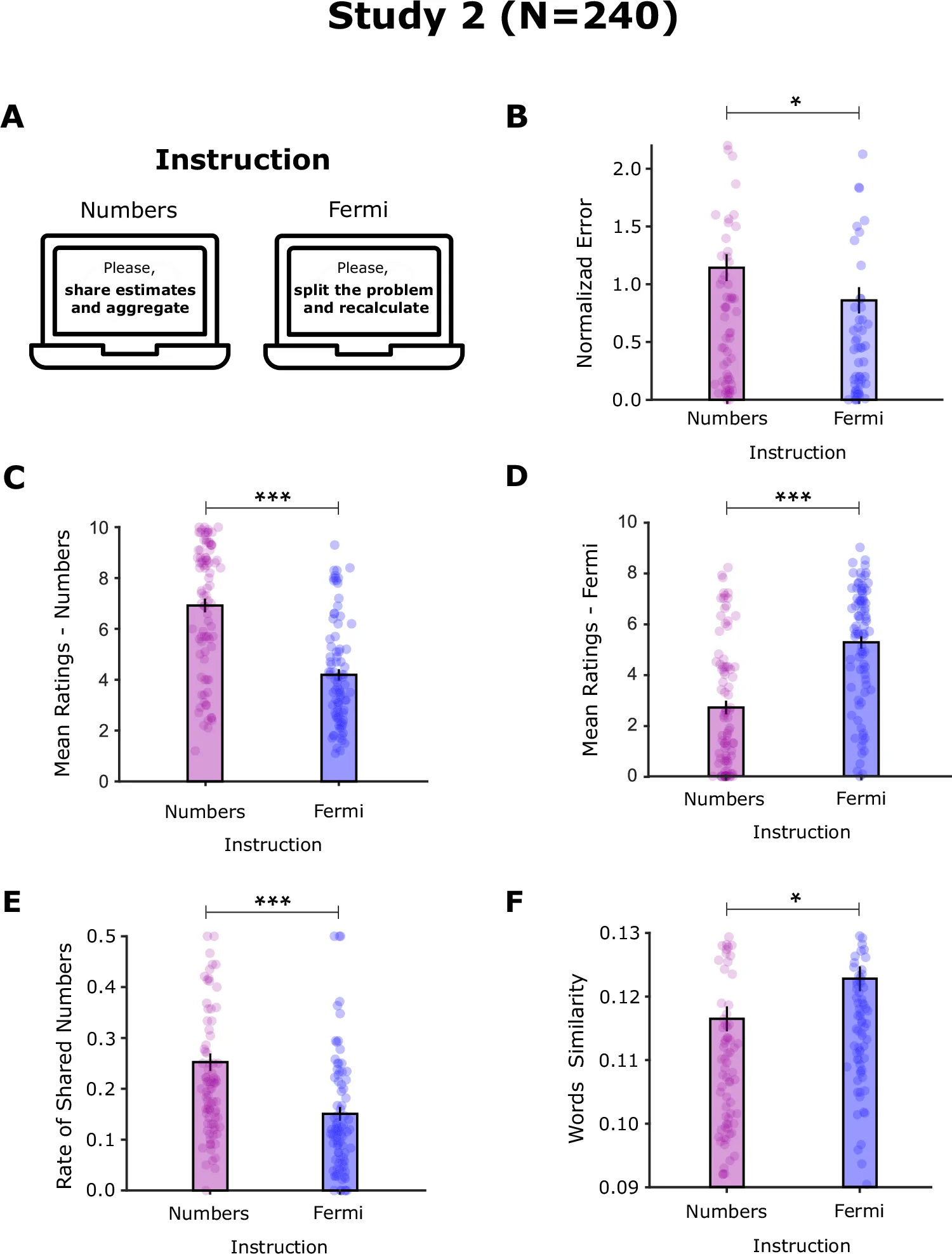
Results of Experiment 2. **A** The experiment had the same procedure as Study 1, except that before the collective deliberation stage, participants received an instruction either to share numbers or to perform approximations and recompute the value. Laptop icon made by Good Ware from www.flaticon.com. **B** Normalized error by instruction condition (Numbers: *n* = 69; Fermi: *n* = 60 biologically independent conversations). **C** Numbers ratings provided by human raters by condition (Numbers: *n* = 89; Fermi: *n* = 90). **D** Fermi ratings provided by human raters by condition (Numbers: *n* = 89; Fermi: *n* = 90). **E** Proportion of shared numbers from the individual stage present in discussions, by condition (Numbers: *n* = 89; Fermi: *n* = 90). **F** Similarity index by instruction condition (Numbers: *n* = 89; Fermi: *n* = 90). In all cases, bars show the mean value, error bars depict s.e.m., and dots show data from individuals. Statistical comparisons are two-sided Mann–Whitney *U* tests with exact *p* values reported in Results. Asterisks denote statistical significance (*p < 0.05, **p < 0.01, ***p < 0.001).

The experiment (Fig. 4A) followed the same procedure as the previous study, with one key difference: after providing their initial estimates and before beginning the deliberation stage, participants watched a short instructional video on how to reach consensus (see “Methods” for full instructions). Half of the groups were randomly assigned to the Fermi condition, where they were instructed to break the problem into smaller estimation steps, make approximations, and recalculate the final answer. The other half were assigned to the Numbers condition, where they were instructed to share their initial estimates and combine them in any way they liked.

As predicted, we observed that groups in the Fermi condition produced collective estimates with lower error than those in the Numbers condition (Fig. 4B; Mann–Whitney *U* test: *z* = 2.1, *p* = 0.039; Cohen’s *d* = 0.26, 95% CI [−0.09, 0.61]). To confirm that this difference reflected the implementation of different strategies, we trained ten naïve raters (4 female; mean age = 18.2 years, s.d. = 0.4), who had not participated in Study 1 and were blind to condition assignments, to evaluate the extent to which each conversation reflected the Fermi or Numbers strategy. As expected, conversations in the Numbers condition received higher Numbers ratings than those in the Fermi condition (Fig. 4C; Mann–Whitney *U* test: *z* = 6.8, *p* < 0.001; Cohen’s *d* = 1.19, 95% CI [0.87, 1.50]). Conversely, conversations in the Fermi condition received higher Fermi ratings than those in the Numbers condition (Fig. 4D; Mann–Whitney *U* test: *z* = 6.3, *p* < 0.001; Cohen’s *d* = 1.06, 95% CI [0.75, 1.38]).

Similarly, following our pre-registration, we found that instructing groups to share numbers led to a higher proportion of interventions containing numbers from the individual stage (Fig. 4E; Mann–Whitney *U* test: *z* = 5.0, *p* < 0.001; Cohen’s *d* = 0.70, 95% CI [0.40, 1.00]). Finally, the Similarity index (previously correlated with Fermi ratings in Study 1) was also higher for groups in the Fermi condition than in the Numbers condition (Fig. 4F; Mann–Whitney *U* test: *z* = 2.5, *p* = 0.012; Cohen’s *d* = 0.34, 95% CI [0.05, 0.64]). We also conducted an additional exploratory analysis to replicate Fig. 2D, E using the data of Study 2 (Fig. S3). We observed that, combining data from both conditions, collective error correlated negatively with Femi ratings (*r* = −0.24, 95% CI [−0.40, −0.07], *p* = 0.005) and positively with Number ratings (*r* = 0.22, 95% CI [0.05, 0.38], *p* = 0.011). Together, these results provide evidence that the instructions effectively modulated the strategies participants used to reach consensus.

We then conducted an additional exploratory analysis examining whether the collective estimate outperformed the individual estimates within each group. For each conversation, we ranked the four individual estimates according to their individual error (from most to least accurate) and computed the probability that the collective estimate was more accurate than each individual estimate (Fig. S4). Collective estimates always improved upon the least accurate individual estimate, regardless of condition. However, differences between strategies emerged for more accurate individuals.

To quantify this pattern, we computed the fraction of initial individual estimates outperformed by the collective answer and compared this measure across conditions. A Wilcoxon rank-sum test indicated that collective estimates under the Fermi condition outperformed a larger fraction of initial estimates than those produced in the Numbers condition (Fermi: 0.73 ± 0.03, Numbers: 0.63 ± 0.03, *z* = 2.33, *p* = 0.029, Cohen’s *d* = 0.44, 95% CI [0.06, 0.82]). This suggests that the advantage of the Fermi condition lies not only in correcting poor initial estimates but also in more often surpassing the most accurate individual judgments in the group.

### Discarding artefactual effects of instructions

To examine whether the observed advantage of the Fermi condition could be explained by mechanisms other than problem decomposition itself, we conducted additional exploratory analyses evaluating two alternative hypotheses. One possibility is that the Fermi instructions simply increased the diversity of information exchanged during discussion, for example, by encouraging participants to articulate the reasons underlying their estimates. If this were the case, the observed gains in the Fermi condition might reflect greater informational diversity rather than problem decomposition per se.

To test this possibility, we implemented an information density metric previously proposed as a proxy for conversational informational diversity^37^. However, we observed that information density did not significantly predict collective error (Pearson correlation: *r* = −0.08, 95% CI [−0.25, 0.10], *p* = 0.384). A Bayesian analysis provided moderate evidence for the null hypothesis (BF_01_ = 6.16, posterior median *ρ* = −0.08, 95% CrI [−0.25, 0.10]), and the conclusion was robust across alternative prior scales, with BF_01_ values between 5.03 and 7.63. These results suggest that the improved performance observed in the Fermi condition cannot be explained simply by increased informational diversity during discussion.

A second possibility is that the Fermi instructions improved performance by increasing coordination or semantic alignment among participants during deliberation. Following this hypothesis, we tested whether conversations in the Fermi condition exhibit stronger semantic convergence over time than those in the Numbers condition. Using the same FastText-based semantic similarity pipeline we previously presented (Fig. 3), we estimated the slope of message-to-message semantic similarity within each conversation. Both results fail to support the coordination hypothesis: We do not observe a statistically significant increase in semantic convergence over time in the Fermi condition (distribution of slopes, mean ± S.D. = −0.005 ± 0.03, *t*-test: *t*(89) = −1.5, *p* = 0.134, Cohen’s *d* = −0.16, 95% CI [−0.37, 0.05]) and the best-fitting slopes do not significantly predict collective error (Pearson *r* = 0.02, 95% CI [−0.15, 0.19], *p* = 0.821). These results remain qualitatively unchanged when applying a 5-message sliding-window approach to compute local convergence patterns (distribution of slopes, mean ± S.D. = −0.0007 ± 0.009, *t*-test: *t*(89) = −0.8, *p* = 0.423, Cohen’s *d* = −0.09, 95% CI [−0.29, 0.12]; correlation with collective error: Pearson *r* = 0.07, 95% CI [−0.11, 0.24], *p* = 0.453). Overall, we did not find evidence supporting the hypothesis that the performance gains observed in the Fermi condition are driven by semantic alignment between individuals during deliberation.

### Linguistic signatures of Fermi ratings

To examine whether conversations identified as Fermi indeed reflect the reasoning processes described in its definition, we developed linguistic indicators capturing the two components of the method: problem decomposition and guesstimation. Problem decomposition was measured through expressions that break the target quantity into component structures (e.g., multiplicative or per-unit reasoning such as “per”, “each”, “every”, or “times”). Guesstimation was measured through linguistic markers reflecting approximate numerical reasoning under uncertainty, including expressions proposing numerical ranges (e.g., “between N and M”, where N and M are numbers) and approximate quantities (e.g., “approximately N”, “around N”, or “about N”, where N is a number).

**Fig. 5:**
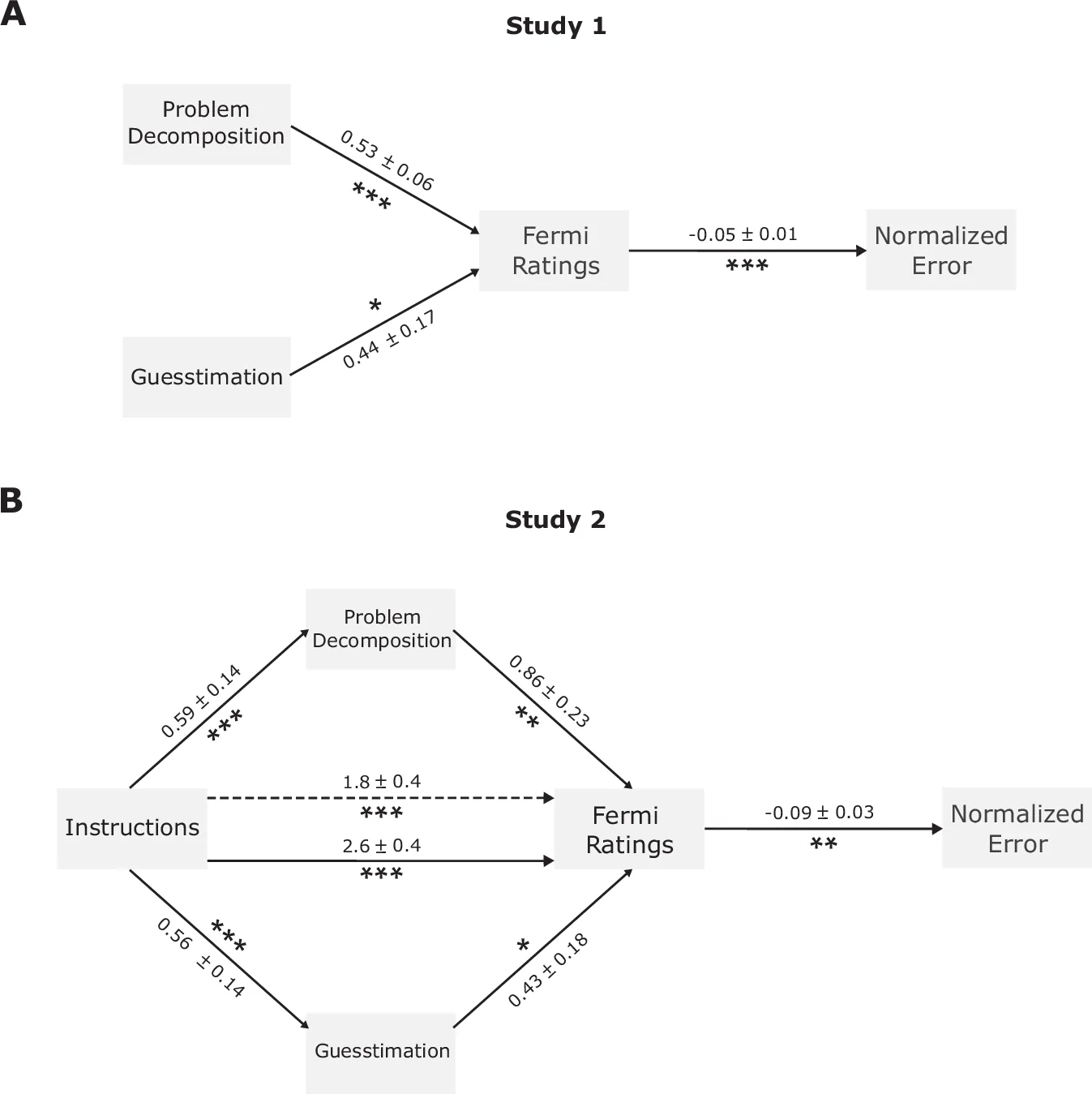
Linguistic mechanisms underlying Fermi ratings. **A** Linguistic markers of problem decomposition and guesstimation in Study 1, derived from the formal definition of the Fermi method, predict human-annotated Fermi ratings. In turn, higher Fermi ratings are associated with lower normalized estimation error. Coefficients correspond to regression estimates (± s.e.m.) from two-sided *t*-tests on multiple linear regression coefficients, and asterisks indicate statistical significance (*p < 0.05, **p < 0.01, ***p < 0.001). Exact *p* values are reported in Results. **B** Mediation analysis for Study 2. Fermi instructions increase the presence of both decomposition and guesstimation markers in the conversations, which in turn predict higher Fermi ratings. Fermi ratings are negatively associated with normalized collective error. The dashed arrow indicates the direct effect of the experimental manipulation on Fermi ratings after accounting for the linguistic mediators. Asterisks denote significance from bootstrap-based two-sided tests on each path coefficient: *p < 0.05, **p < 0.01, ***p < 0.001. Exact *p* values are reported in Results.

Across studies, these linguistic indicators predict the extent to which conversations exhibit Fermi-style reasoning. In Study 1, both decomposition and guesstimation markers significantly predict human-annotated Fermi ratings (Fig. 5A, *β*_PD_ = 0.54, *p* < 0.001; *β*_GE_ = 0.43, *p* = 0.011). In Study 2, the effect of the experimental manipulation (Fermi vs. Numbers instructions) on Fermi ratings is mediated by the increased presence of these markers in the conversations (Fig. 5B, total effect: 2.56 ± 0.36, *p* < 0.001, direct effect: 1.86 ± 0.37, *p* < 0.001, indirect effect of PD: 0.46 ± 0.12, *p* < 0.001; indirect effect of GE: 0.25 ± 0.11, *p* = 0.012).

Taken together, these results indicate that conversations identified as exhibiting Fermi-style reasoning systematically contain the linguistic signatures predicted by the formal definition of the method. This provides converging evidence that the strategy implemented in the experiments reflects the combination of problem decomposition and rough approximation that characterizes the Fermi method.

While Study 2 offers insights into the drivers of collective accuracy and supports the idea that groups using the Fermi method achieve better estimates, another explanation is possible. The observed reduction in error in the Fermi condition may not be due to collective problem decomposition itself, but rather to individual participants applying the Fermi method within the group, without necessarily engaging in a group-level reasoning process^38^. In other words, the strategy might improve accuracy simply by being used, whether individually or collectively. To test this possibility, we conducted another experiment designed to disentangle individual and collective contributions to the effectiveness of the Fermi method.

### Study 3: Fermi method is more accurate in groups than alone

**Fig. 6:**
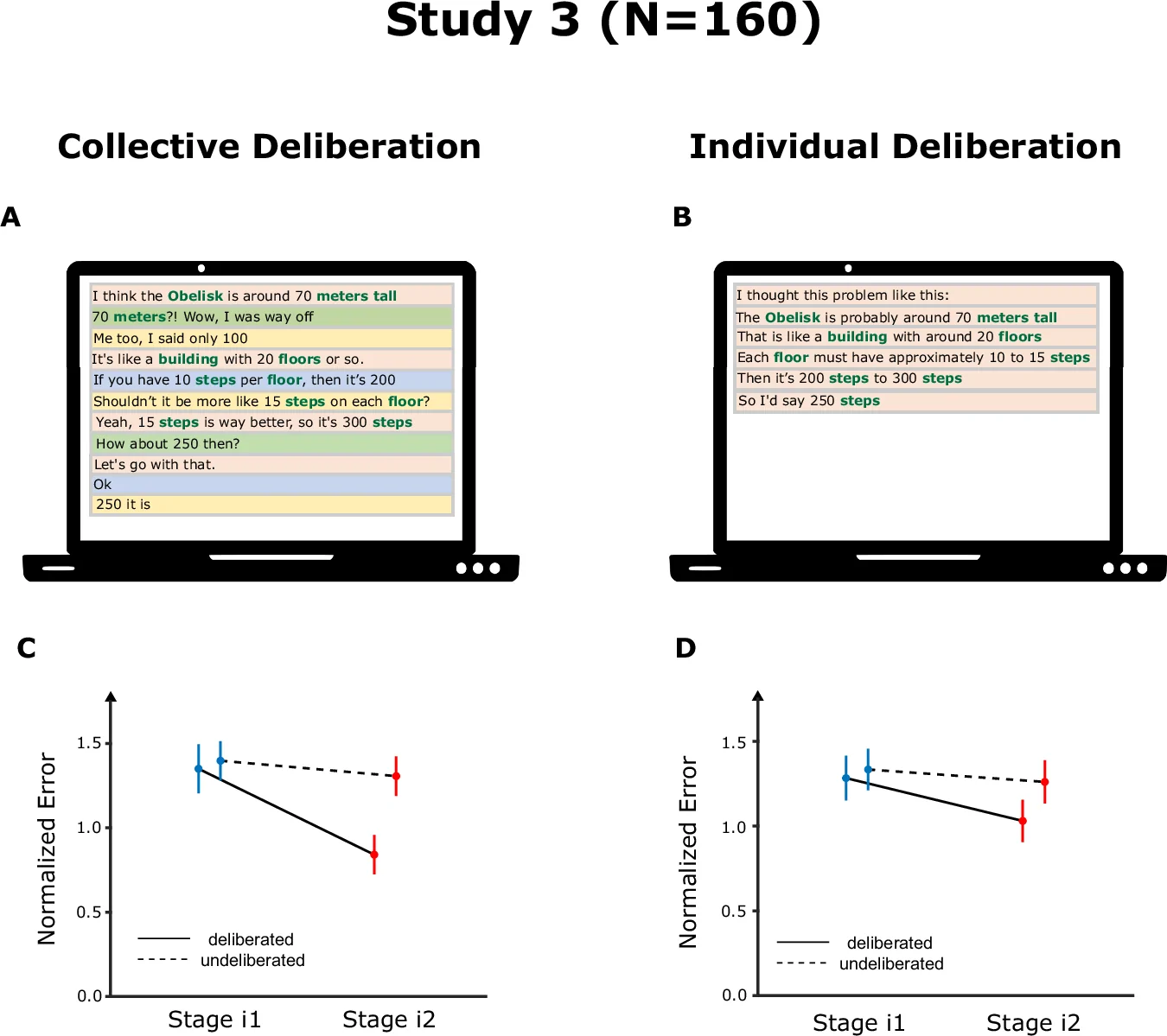
Empirical results for Experiment 3. The procedure was the same as that of the previous experiment, with two exceptions: **A** half of the participants were divided into groups and received the original Fermi instruction, applying the Fermi method as a group. **B** The remaining half proceeded individually, and received a modified Fermi instruction prompting them to also apply the Fermi method individually (writing down their procedure). In both **A** and **B**, the laptop icon was made by Maxim Basinski Premium from www.flaticon.com. **C** Mean normalized error for the individual stages, for both deliberated (stage i1: *n* = 212; stage i2: *n* = 199 participant-question observations from biologically independent participants) and undeliberated (stage i1: *n* = 291; stage i2: *n* = 283) questions, for participants in the collective condition. The error bars correspond to the 95% confidence intervals. **D** Mean normalized error for the individual stages for participants that deliberated individually, for both deliberated (stage i1: *n* = 231; stage i2: *n* = 220 participant-question observations from biologically independent participants) and undeliberated (stage i1: *n* = 275; stage i2: *n* = 258) questions. Error bars represent 95% confidence intervals. We followed the same procedure as for the previous panel.

*N* = 160 participants (95 female, mean age 29.8 years, s.d. 10.9 years) participated in Study 3 (pre-registered at https://aspredicted.org/SZ5_MNP). A random half of the participants were divided into 20 groups of 4 individuals, and the remaining 80 performed the study individually. For half of the participants, the experiment followed exactly the same procedure as the Fermi condition in the previous study: Participants received instructions to apply the Fermi method and deliberated in groups to reach a collective estimate (Fig. 6A). The key difference in this experiment was the addition of an individual condition, which applied to the other half of participants, who worked alone rather than in groups. These participants were instructed to apply the Fermi method individually and to write their reasoning and calculations in a chat window, without interacting with others (Fig. 6B). All written responses across both conditions were recorded (see “Methods” for details).

This design allowed us to directly compare the effectiveness of applying the Fermi method individually versus in groups as a strategy for improving accuracy (Fig. 6C, D). Following our pre-registration, we first tested whether the final individual error at stage i2 differed between the Collective and Individual conditions. A mixed-effects linear regression with normalized individual error as the dependent variable, a dummy variable coding for the Collective condition, and random intercepts for the eight questions showed that participants in the Collective condition produced significantly lower errors at stage i2 than those in the Individual condition (*β* = −0.24 ± 0.07, 95% CI [−0.38, −0.10], *p* < 0.001). A Wilcoxon rank-sum test comparing the distributions of individual errors across conditions yielded the same conclusion (*z* = 3.12, *p* = 0.002, Cohen’s *d* = 0.26, 95% CI [0.07, 0.44]).

To facilitate interpretation of how deliberation affected performance relative to each participant’s initial estimate, we also report an exploratory analysis examining the reduction in error between the initial individual estimates (stage i1) and the final individual estimates (stage i2). First, we focused on the collective condition (Fig. 6C). We observed that, while participants showed a modest improvement for non-deliberated questions (paired Cohen’s *d* = 0.16, 95% CI [0.04, 0.29]), the improvement was greater for collectively deliberated questions (paired Cohen’s *d* = 0.40, 95% CI [0.26, 0.54], *β* = −0.45 [−0.59, −0.31], SE = 0.07, *t*(469) = −6.36, *p* < 0.001, *R*² = 0.10).

We then examined the individual deliberation condition (Fig. 6D), where participants worked alone but were instructed to apply the Fermi method. As in the collective condition, participants showed a modest improvement for non-deliberated questions (paired Cohen’s *d* = 0.17, 95% CI [0.05, 0.29]) and a larger improvement for the ones that underwent individual deliberation (paired Cohen’s *d* = 0.56, 95% CI [0.41, 0.71], *β* = −0.17 [−0.28, −0.07], SE = 0.05, *t*(469) = −3.28, *p* = 0.001, *R*² = 0.02). This suggests that applying the Fermi method individually may result in greater accuracy.

To formally compare conditions, we examined the reduction in error in the collective and individual conditions separately for deliberated and undeliberated items. For deliberated items, error reduction was larger in the collective condition than in the individual condition (Mann–Whitney *U* test: *z* = 6.3, *p* < 0.001; Cohen’s *d* = 0.58, 95% CI [0.39, 0.78]). No such difference was observed for undeliberated items (Mann–Whitney *U* test: *z* = 1.0, *p* = 0.309; Cohen’s *d* = 0.05, 95% CI [−0.12, 0.22]). These results suggest that, although the Fermi method improves accuracy when applied individually, collective deliberation provides an additional benefit beyond individual reasoning alone.

### Fermi estimation benefits groups more than individuals

Study 3 allowed us to examine whether the benefits of the Fermi method differ depending on whether the strategy is applied individually or collectively. In this experiment, all participants were instructed to apply the Fermi method, but some deliberated individually while others deliberated in groups. In both cases, participants improved their estimates relative to their initial responses but the magnitude of this improvement was larger in the collective condition (Fig. 6C, D).

One possible explanation for this effect is that collective deliberation enabled metacognitive processes that are difficult to reproduce in isolation. Prior work in collective cognition suggests that interactive reasoning can benefit from supra-personal monitoring processes, such as detecting and correcting errors in others’ reasoning^18,39,40^. In the context of Fermi estimation, this mechanism is plausible because decomposing a problem generates multiple intermediate assumptions that can be re-evaluated during deliberation.

To test this possibility, we conducted an exploratory analysis of the language produced by participants in Study 3. We constructed a linguistic indicator capturing error-correction dynamics, based on expressions typically associated with evaluating or adjusting magnitudes (e.g., phrases like “too little” or “too much”, or evaluative corrections such as “better”). These expressions occurred more frequently in collective deliberation than in individual reasoning (Fig. S5, unpaired *t*-test: *t*(360) = −6.7, *p* < 0.001, Cohen’s *d* = −0.91, 95% CI [−1.19, −0.64]), and higher levels of such language were associated with lower estimation error in the subsequent individual stage (Pearson *r* = −0.18, *p* < 0.001, 95% CI [−0.28, −0.09]). Importantly, these markers did not significantly differ between the Fermi and Numbers conditions in Study 2 (Fig. S5, unpaired *t*-test: *t*(177) = 0.9, *p* = 0.363, Cohen’s *d* = −0.14, 95% CI [−0.43, 0.16]), and did not predict collective error in Study 1 (Pearson *r* = 0.03, 95% CI [−0.09, 0.12], *p* = 0.579). A Bayesian analysis provided strong evidence for the null hypothesis (BF_01_ = 13.80, posterior median *ρ* = 0.02, 95% CrI [−0.09, 0.12]), and the conclusion was robust across alternative prior scales, with BF_01_ values between 11.23 and 17.14. This pattern suggests that error-correction dynamics help explain why collective deliberation amplifies the benefits of the Fermi method, rather than why the Fermi method itself improves performance relative to number sharing.

### Can large language models detect Fermi reasoning?

Because human annotation procedures can, in principle, be influenced by the instructions provided to raters, we conducted an additional analysis to evaluate whether Fermi ratings could be reproduced using an independent procedure based solely on the formal definition of the method (presented in the Introduction). Specifically, we generated AI-based Fermi ratings using large language models (LLMs)^41^. For each conversation, the model was provided with the theoretical definition of the Fermi method and asked to rate, on a 0–10 scale, the extent to which the procedure in the conversation matched that definition.

To ensure that the results were not dependent on the idiosyncrasies of a particular model, we repeated this procedure using five different LLMs (gpt-4.1-mini, gpt-5-mini, gpt-5.2, claude-haiku-4.5, and claude-sonnet-4.6). Across models, the resulting ratings show strong correlations with the mean human annotations (Pearson *r* > 0.7, *p* < 0.001 for all comparisons, Table S4) as well as strong correlations among models themselves (Pearson *r* > 0.8, *p* < 0.001 for all comparisons, Table S4), indicating a high level of agreement across independent rating procedures.

Importantly, the main empirical relationships reported in this paper replicate when using AI-based ratings in place of the human annotations. Across all three studies, higher Fermi ratings generated by the language models are associated with lower estimation error (Table 1). In addition, these ratings show the same expected relationships with the linguistic indicators introduced above: they are positively associated with both problem decomposition and guesstimation markers (Table 1).

**Table 1:** Replicating main results using LLM-based Fermi ratings

	Study 1 (*m* = 426)			Study 2 (*m* = 179)			Study 3 (*m* = 362)		
	Error	PD	GE	Error	PD	GE	Error	PD	GE
GPT 4.1 mini	−0.20	0.38	0.13	−0.21	0.39	0.33	−0.24	0.48	0.13
GPT 5.1 mini	−0.22	0.39	0.19	−0.28	0.44	0.32	−0.30	0.45	0.21
GPT 5.2	−0.20	0.45	0.12	−0.27	0.45	0.30	−0.27	0.50	0.11
Claude Haiku 4.5	−0.25	0.35	0.12	−0.31	0.40	0.35	−0.31	0.35	0.15
Claude Sonnet 4.6	−0.22	0.39	0.15	−0.29	0.46	0.34	−0.32	0.30	0.05

For each of five large language models (rows), the table reports Pearson correlation coefficients between LLM-generated Fermi ratings and key variables across the three studies (*m* is the number of conversations). Columns show correlations with normalized estimation error (Error), problem decomposition markers (PD), and guesstimation markers (GE). Full statistics, including exact *p* values and 95% confidence intervals, are reported in Table S6.

These results provide converging evidence that the Fermi ratings capture a stable and identifiable reasoning pattern rather than an artifact of the human annotation procedure. Conversations rated as exhibiting stronger Fermi-style reasoning (whether by human raters or LLMs) systematically display the linguistic signatures predicted by the formal definition of the method and are associated with improved collective accuracy.

## Discussion

Across three studies, we provide converging evidence that small groups can reach more accurate estimates than large, independent crowds when they engage in problem decomposition, a strategy we term “Collective Fermi Estimation”. This approach involves making rough calculations and computing an approximate solution, rather than merely aggregating initial signals. In Study 1, we found that groups exhibiting higher use of this strategy, as determined by trained human raters, produced significantly more accurate estimates. Study 2 established a causal link: when groups were explicitly instructed to use the Collective Fermi Estimation strategy, their performance improved relative to groups prompted to aggregate their individual estimates. Finally, in Study 3, we demonstrated that the advantage of this strategy lies in its collective application: Aggregating the estimates of individuals who each applied the strategy independently yielded poorer performance than when the reasoning occurred through group deliberation^38^.

Another contribution of this work is the development of a variety of computational methods to detect whether a group employed the Collective Fermi strategy based on the content of its conversation. Using word embeddings to measure the semantic similarity between words in the deliberation and those in the original estimation question, we constructed a low-cost index that correlates with human Fermi ratings and, critically, predicts group accuracy. This method outperformed simpler proxies such as word count or lexical overlap, suggesting that capturing the semantic structure of a group’s reasoning is essential for detecting meaningful deliberation. We also developed a pipeline to detect Fermi-style reasoning using LLMs and show that our results replicate using this fully automated approach. These results have practical implications as they provide ways of extracting information from crowdsourced estimates under social influence.

These findings also contribute to the broader literature on collective intelligence and the wisdom of crowds by helping to clarify when and how social interaction can improve collective accuracy. Prior work has shown that deliberation can either impair or enhance group performance, depending on the context and the nature of the interaction^12,15,18,42^. Here, we provide evidence in support of the hypothesis that deliberation grounded in reasoning (rather than information exchange) is a key mechanism driving superior group performance.

However, several limitations remain. As with much of the crowd wisdom literature, it is unclear whether our findings extend to domains where correct answers are categorical rather than continuous (for a notable exception, see Becker et al.^43^). Moreover, many real-world group decisions involve not only categorical judgments, but also explanations, advice, planning, or the analysis of complex situations, for which numerical aggregation procedures do not directly apply. Our findings, therefore, speak most directly to a relatively narrow class of estimation problems. However, the mechanism we study (i.e., collective decomposition of a complex problem into simpler components) may generalize beyond numerical estimation. In principle, many complex judgments can be structured as a set of simpler sub-questions whose answers can then be integrated into a final decision. Future work should examine whether collective Fermi-style reasoning can provide similar benefits in domains where the outcome is not a numerical estimate but rather a categorical judgment, explanation, recommendation, or plan of action.

Another limitation of the present design is that, in Studies 1 and 2, questions that were deliberated differed from those that were not deliberated not only because they involved group interaction, but also because participants could devote more attention to them. Because deliberated questions were discussed collectively for up to 5 min, whereas undeliberated questions were answered individually within the overall task time, the two types of questions may differ in the amount of individual attention allocated to them. As a result, comparisons between deliberated and undeliberated questions in those studies (which we present only in Fig. 1D) cannot cleanly isolate the effect of group interaction from differences in attention per question. Importantly, however, the central comparisons of the present work do not rely on this contrast. In Study 1, the key analyses focus on variation in how groups reason about deliberated questions. In Study 2, the main comparison is between two instruction conditions applied to deliberating groups. Finally, Study 3 provides a cleaner test of the role of social interaction by comparing individuals deliberating alone with groups deliberating together, holding the opportunity for deliberation constant. Together, these analyses suggest that while differences in attention may contribute to some contrasts, the central findings regarding collective Fermi-style reasoning are not driven by this feature of the design.

Beyond hypothesis testing, we believe our findings carry broader implications for the design of deliberative environments. The wisdom of crowds has long been interpreted as a model for the epistemic benefits of democratic judgment^2,3,44^. Within this framework, a central theoretical distinction has been drawn between “voting mechanisms,” which aggregate individual preferences, and “deliberative mechanisms,” which emphasize shared reasoning and mutual justification^38^. While deliberative democracy rests on the premise that genuine deliberation can improve collective decisions over information sharing^45^, empirical demonstrations of this principle have remained limited. Our findings help fill this gap by showing not only that groups naturally engage in collective reasoning under certain conditions, but also that they can be prompted to adopt such a mindset, yielding measurable epistemic benefits.

Overall, this work shows that collective reasoning, and in particular reasoning by approximation, underlies enhanced collective accuracy during deliberation, and offers tools to detect and foster such processes, contributing to a basic understanding of human group decision-making and informing practical applications aimed at improving crowdsourcing strategies.

## Methods

### Participants

All research complied with relevant ethical regulations. All study protocols were approved by the ethics committee at Universidad Abierta Interamericana (Buenos Aires, Argentina; protocol 0-1108). All participants provided informed consent prior to participation.

Participants were recruited using non-probabilistic convenience and snowball sampling, through three channels: (1) emails sent by Universidad Torcuato Di Tella to its students, (2) the participant database at the Laboratorio de Neurociencia, Universidad Torcuato Di Tella, and (3) invitations shared through researchers’ personal social media accounts. Participation was voluntary and could be withdrawn at any time. All data were anonymized. Gender was self-reported (male, female, or other); sex was not assessed. Sex and gender were not considered in the study design, as our research questions did not concern sex- or gender-related hypotheses. Overall, we collected data from 900 individuals, all residents of Argentina, across three samples: Study 1 involved 500 participants (288 female, mean age 25.4 years, s.d. 7.7 years), in Study 2 we recruited 240 participants (148 female, mean age 30.6 years, s.d. 9.8 years), and Study 3 involved 160 participants (95 female, mean age 29.8 years, s.d. 10.9 years).

In all cases, participants were entered in a draw for 5 prizes of 100 USD each. This procedure led to a participation fee with an expected value of roughly 6 USD per hour of experiment, which is a fair rate based on the guidelines of subject participation in Buenos Aires, Argentina.

### Statistics and reproducibility

Sample sizes for Studies 2 and 3 were determined by power analyses based on Study 1 data; Study 1 was exploratory, and no a priori sample size calculation was performed. Following the procedure implemented in previous research^12,46^, data were excluded using two preregistered criteria: estimates that deviated more than 2.5 median absolute deviations from the median distribution in the initial individual stage, and groups with at least two participants leaving the chatroom before the end of the deliberation stage. In all studies, allocation of participants into groups, the order of questions, and the subset of questions selected for the deliberation stage were randomized. In Studies 2 and 3, assignment to experimental conditions was also randomized. All assignments were performed automatically by the data collection platform. Participants were blind to the study hypotheses, and platform-based automatic assignment to groups and conditions also kept investigators blind to allocation during data collection. There were no deviations from the pre-registrations.

Unless otherwise stated, all statistical tests in the paper were two-sided.

### Study 1

The study consisted of three stages. In the first stage, participants independently responded to a series of eight numerical estimation questions within a 5-min time limit. Alongside their answers, they indicated their level of confidence using a slider bar ranging from low (0) to high (100). Moving on to the second stage, participants communicated via chat to try to reach a consensus on values for four of the eight questions from the first stage. Participants were instructed to try to reach consensus with a 5-min time limit. If consensus was achieved earlier, participants had to wait until the 5 min elapsed before moving on to the next question. During this stage, participants were not asked to report their confidence levels. The third stage of the study mirrored the first stage, wherein participants individually tackled the same numerical estimation questions within a time limit of 5 min.

### Study 2

Study 2 was identical to Study 1, except that they were given instructions on how to reach consensus. In addition to the previous procedure, participants were allocated to one of two conditions. In the *Numbers Condition*, they were explicitly instructed to “share their initial numerical estimates”. In the *Fermi Condition* they were explicitly instructed to “divide the problem into smaller estimation problems and perform all relevant computations to reach their final answer”. All hypotheses and data analysis plans were preregistered on 17 March 2023 at https://aspredicted.org/TL2_Q1Q.

### Study 3

In Study 3, all participants received the same instructions as in the Fermi Condition from the previous study. However, a random half were assigned to groups of four, while the other half worked individually. Those in the individual condition were asked to apply the Fermi Method on their own, documenting their procedure in the same chatroom used by the groups, though they remained alone for the entire experiment. This study was preregistered on 20 March 2023 at https://aspredicted.org/SZ5_MNP.

### Estimation questions

The eight questions were presented in random order in the first stage. A random half of them were selected for stage 2. For the third stage, the questions were presented in the same order as the first stage. The complete set of questions and correct answers is available at Table S2.

### Supervised metrics

For this approach, we trained ten raters (8 female; mean age = 22.8 years, s.d. = 5.4), who were naïve to the hypotheses of the study, on identifying conversations that used the Fermi and Numbers strategies. Then, they individually read each conversation and determined how much of the Fermi method was actually used on a scale from 0 (completely absent) to 10 (completely present). They were also asked to rate how much the groups implemented the Numbers strategy, consisting of exchanging their answers and combining their initial numerical estimates to obtain a final group answer. The order of presentation of the explanation of the two strategies and the conversations to be rated was randomized across raters. The detailed instructions provided to the raters, including the alternative definition of the Fermi method, are available in Supplementary Note 1.

### Similarity measures

We computed the semantic similarity between the conversation and the task using natural language processing^46,47^. To this aim, we leverage pre-trained word embeddings, specifically FastText trained in Spanish^36^. Word embeddings are vector representations of words in a way that semantically related words have similar vectors. We employed FastText to measure the similarity between the question asked and the entire conversation. Specifically, our method calculates the similarity between all words in the question and all words in the conversation, with the exclusion of stop words and small words. This process generates a distribution of similarity values from which we take its mean, given by Eq. (1):

1 $$S=\frac{1}{n\cdot m}{\sum }_{i=1}^{n}{\sum }_{j=1}^{m}\left(\frac{{{{\bf{V}}}}_{{{\boldsymbol{i}}}}\cdot {{{\bf{W}}}}_{{{\boldsymbol{j}}}}}{\Vert {{{\bf{V}}}}_{{{\boldsymbol{i}}}}\Vert \Vert {{{\bf{W}}}}_{{{\boldsymbol{j}}}}\Vert }\right)$$

### Non-parametric normalization

The distributions of estimates for each question were centered on different values. To normalize these distributions, we used a non-parametric approach, originally developed in the outlier detection literature, and used in previous studies examining the wisdom of crowds^12,48^. This procedure involves subtracting the median value of the distribution and then dividing by the median absolute deviation, leading to a non-parametric z-score value. The rationale for normalizing our data was twofold. First, we used this procedure to reject outliers in the distribution of responses. Following previous studies, we discarded all responses that deviated from the median by more than 2.5 times the median absolute deviation. The second purpose of normalization was to average our results across different questions. This helps the visualization of our data, but our main findings can be replicated without any normalization (Table S5).

### Robustness checks against answer look-up

All studies were conducted online. Although participants were instructed not to search for answers during the task, the online setting does not technically prevent such behavior. We therefore conducted additional robustness checks to evaluate whether the results could be driven by participants looking up the correct answers.

First, we examined how often group conversations produced the exact true value of the target quantity. If participants had systematically searched for the answers online, we would expect exact matches to occur frequently. In practice, only a small fraction of conversations produced answers that matched the true value exactly (between 0.6% and 2.8% across studies). For some questions, such matches could plausibly without looking up the answer because the correct value falls within commonly guessed ranges (e.g., “How many kilograms of ice cream does a person in Argentina eat per year?”). Therefore, to address this concern conservatively, we repeated the main analyses after excluding all conversations that produced the exact correct answer. The results remain qualitatively unchanged (e.g., Mann–Whitney *U* test between Fermi and Numbers conditions in Study 2: *z* = 1.97, *p* = 0.036; Cohen’s *d* = 0.25, 95% CI [−0.05, 0.55]).

### Evaluating variability between human raters

We studied whether the relationship between Fermi ratings and collective accuracy could reflect variability in how annotators interpreted the coding scheme. To evaluate this possibility, we examined the variance of Fermi ratings across annotators at the level of individual conversations. Disagreement among raters was generally small (mean SD ≈ 0.16 Likert points), indicating high consistency in the evaluations. Moreover, between-rater variance did not differ significantly across experimental conditions (Mann–Whitney *U* test: *z* = 1.8, *p* = 0.066; Cohen’s *d* = 0.29, 95% CI [0.00, 0.59]) and did not correlate with either the linguistic markers of Fermi reasoning or collective estimation error (problem decomposition: *r* = 0.07, 95% CI [−0.08, 0.21], *p* = 0.364; guesstimation: *r* = 0.09, 95% CI [−0.05, 0.24], *p* = 0.217; collective error: *r* = 0.02, 95% CI [−0.15, 0.20], *p* = 0.781). As an additional robustness check, we repeated the main analyses after excluding conversations whose between-rater variance fell within the highest quartile of the distribution. The results remain unchanged under this restriction (Pearson correlation between Fermi ratings and collective error: *r* = −0.20, p = 0.041, 95% CI [−0.385, −0.008]), indicating that the observed relationship between Fermi ratings and estimation accuracy is not driven by conversations for which annotators showed greater disagreement.

### Linguistic markers

To quantify the extent to which conversations implemented specific reasoning processes, we developed a set of theory-driven linguistic indicators derived from the formal definition of the Fermi method and from the hypothesis of error-correction dynamics introduced in the main text. In all cases, the indicators were computed at the level of the conversation by quantifying the prevalence of stylized expressions associated with each target process. Text was preprocessed in a case-insensitive manner, and accent marks did not affect matching.

To capture problem decomposition, we identified expressions typically used to break a problem into multiplicative or per-unit subcomponents. These included constructions involving the Spanish preposition “*por*” (which means “*by*”, “*per*”, or “*times*”, depending on context) followed by a noun or number, multiplicative operators (e.g., “*x*”, “*”) followed by a noun or number, and expressions based on the word “*cada*” (which translates to “*each*” or “*every*”) followed by a noun or number.

To capture guesstimation (i.e., approximate numerical reasoning under uncertainty), we identified expressions typically used to propose rough estimates. These included numerical ranges introduced by “*entre”* (meaning *“between”*) followed by a number, or modifiers indicating numerical uncertainty, such as “*aproximadamente*” (or the colloquial “*aprox*”, meaning “*approximately*”) or “*alrededor*” (“*about*”) followed by a number.

To capture the explicit evaluation or revision of quantities, we identified expressions associated with magnitude adjustment and correction. These included forms of the verb *ser* (“to be”) combined with *mucho* (“a lot” or “too much”), *poco* (“a little” or “too little”), or *demasiado* (“too much”), as well as evaluative correction terms such as *mejor* (“better”).

### LLM-based ratings

To obtain AI-based Fermi ratings, we queried each conversation using the application programming interface (API) of five large language models: gpt-4.1-mini, gpt-5-mini, gpt-5.2, claude-haiku-4.5, and claude-sonnet-4.6. Each model received the same prompt, which presented the formal definition of the Fermi method and asked the model to evaluate the extent to which the conversation implemented that reasoning procedure on a 0–10 scale. Responses were constrained to return a single numerical score only.

The exact prompt was:

*The Fermi Method consists of breaking the problem into simpler components, making rough estimations for each component, and calculating the final answer based on those estimations. You are an expert on the Fermi Method. Based on the definition above, evaluate the extent to which the participants in the following conversation used the Fermi Method. Rate the use of the Fermi Method on a scale from 0 to 10, where:*

*− 0 means the method was not used at all*,

*− 10 means the method was fully and explicitly used throughout*.

*Provide a single numerical score (0–10)*.

*Return only the score (from 0–10), do not include any additional text*.

*Conversation: [conversation text]*

All conversations were rated independently by each model using the same prompt and input format. The resulting scores were then used as AI-based Fermi ratings in the analyses reported in the main text.

### Bayes factor analyses

To complement frequentist tests with evidence regarding the presence or absence of effects, we computed Bayes factors using the default JZS prior specification of Rouder et al.^49^: a Cauchy distribution on the standardized effect size *δ* centered at zero with scale parameter r= $\sqrt{2}/2$. Bayes factors were computed with the bayesFactor MATLAB toolbox. For each Bayes factor, we additionally summarize the posterior distribution of *δ* by its median and 95% credibility interval, obtained by numerical integration of the JZS posterior. To assess sensitivity to the prior specification, we recomputed each Bayes factor with a wide (r= 1) and an ultrawide (r= $\sqrt{2}$) Cauchy prior.

For Bayesian inference on Pearson correlations, we computed Bayes factors under a stretched-beta prior on the population correlation *ρ* ∈ [−1, 1], with the default scale parameter *κ* = 1 corresponding to a uniform prior on *ρ*^50,51^. The sampling distribution of the Fisher-transformed correlation was modeled as Normal with variance 1/(n−3), a virtually exact approximation for our sample sizes (*n* > 100). For each correlation, we additionally report the posterior median and 95% credibility interval of *ρ*, obtained by numerical integration. To assess sensitivity to the prior specification, we recomputed each Bayes factor under a wider (${{\rm{\kappa }}}=\sqrt{2}$) and a narrower (${{\rm{\kappa }}}=$ $\sqrt{2}/2$) scale. We follow the conventional interpretation where 3 < BF_01_ ≤ 10 denotes moderate and 10 < BF_01_ ≤ 30 denotes strong evidence for the null hypothesis^52^.

### Mixed-effects models

To compare participants’ estimates from the first and third stages in Study 1 (Fig. 1D), we used a generalized linear mixed-effects model, with random intercepts for each question, and fixed effects for each group and question.

The generalized linear model was given by Eq. (2):

2 $$\Delta {E}_{{ij}}={\beta }_{0}+{\beta }_{1}\,{{{\rm{deliberated}}}}_{{ij}}+{\mu }_{j}+{\varepsilon }_{{ij}}$$

where $\Delta {E}_{{ij}}$ denotes the change in error for observation i associated with question j and ${{deliberated}}_{{ij}}$ is a binary predictor indicating whether the question was deliberated. The model includes a fixed intercept and fixed effect for deliberation, as well as a random intercept ${\mu }_{j}$ to account for repeated observations across questions. The same type of generalized linear mixed-effects models was used in Study 3 (Fig. 6C, D) with a dummy coding for the collective condition.

### Reporting summary

Further information on research design is available in the Nature Portfolio Reporting Summary linked to this article.

## Supplementary information

## Data Availability

The data (both raw and processed) supporting our analyses are available at the Open Science Framework (https://doi.org/10.17605/OSF.IO/BRU7K)^53^.

## Code availability

The code supporting our analyses is available at the Open Science Framework (https://doi.org/10.17605/OSF.IO/BRU7K)^53^. The chatroom platform code is available at https://github.com/facuzeta/chat-room-platform.
