# Supplementary material for: Collective problem decomposition improves the wisdom of deliberative crowds
Source: Nat Commun. 2026 Aug 5;17:9431. doi: 10.1038/s41467-026-76365-y (PMC13538491; doi:10.1038/s41467-026-76365-y)
Supplement: Supplementary file 1 [file 41467_2026_76365_MOESM1_ESM.pdf]

Supplementary Information

Albarracín, L., & Gorgorió, N. (2014).	(The Fermi method is) "…based on designing a problem-solving strategy adapted to the problem which consists of breaking it into subproblems that may be solved separately by means of their respective estimations."
Albarracín, L., & Gorgorió, N. (2019).	"The procedure proposed by Fermi to his students was to decompose the original problem into simpler sub-problems to reach a solution of the original question by means of reasonable estimates or educated guesses"
Abay, S., & Filiz, S. B. (2020).	"Fermi problems are open-ended, non-routine problems that require students to make systematic guesses by making assumptions before starting on a solution using simple calculations."
Gomilsek et al. (2024)	"Fermian strategies prescribe decomposing an estimation problem into subtasks, solving the subtasks separately, and ultimately integrating those solutions into a final estimate."
Albarracín, L., & Ärlebäck, J. B. (2025)	"The Fermi estimation method is the classic approach to solving Fermi problems, in which assumptions are made to decompose the original problem into simpler subproblems that are solved separately using reasoned estimates based on knowledge of the real world."

Table S1: Definitions of Fermi problems or procedures in previous literature. Despite differences in terminology, these definitions converge on two core reasoning steps: problem decomposition and "guesstimation" of intermediate values which are then recombined to produce a final estimate.

Question	Correct Answer
How many goals were scored in total at the 2014 FIFA World Cup played in Brazil?	171
How many steps are there on the internal staircase of The Obelisk of Buenos Aires?	206
How many people (in millions) live in South America?	373
What is the total length (in meters) of line A of the Buenos Aires underground network?	10800
Until 2021, in how many Copa Libertadores finals has an Argentine team participated in?	37
How many kilograms of ice cream does a person in Argentina eat per year?	7
How many episodes are there in the American television series "Friends"?	236
How many countries have territory in the southern hemisphere, excluding the Antarctic continent?	48

Table S2: Questions tested in all experiments and their correct answers

Ind.	Accuracy			Confidence		
	TOST (d_1 = 20%, d_2 = 30%)		Bayes Factor	TOST (d_1 = 20%, d_2 = 30%)		Bayes Factor
1	p_1 ($x < d_1$)	< 0.001	15.0	p_1 ($x < d_1$)	< 0.001	0.005
	p_2 ($x > d_2$)	< 0.001		p_2 ($x > d_2$)	< 0.001	
	C.I.	[0,0.022]		C.I.	[-0.036,-0.016]	
2	p_1 ($x < d_1$)	< 0.001	52.7	p_1 ($x < d_1$)	< 0.001	18.5
	p_2 ($x > d_2$)	< 0.001		p_2 ($x > d_2$)	< 0.001	
	C.I.	[-0.008,0.013]		C.I.	[-0.019,0.001]	
3	p_1 ($x < d_1$)	< 0.001	48.7	p_1 ($x < d_1$)	< 0.001	3.90
	p_2 ($x > d_2$)	< 0.001		p_2 ($x > d_2$)	< 0.001	
	C.I.	[-0.014,0.007]		C.I.	[0.004,0.024]	
4	p_1 ($x < d_1$)	< 0.001	12.2	p_1 ($x < d_1$)	< 0.001	0.34
	p_2 ($x > d_2$)	< 0.001		p_2 ($x > d_2$)	< 0.001	
	C.I.	[-0.020,0.001]		C.I.	[0.010,0.032]	

Table S3: Results from two one-sided tests (TOST) of equivalence and Bayes factor analyses for the proportion of interventions of each individual (1-4), ordered by accuracy (first columns) and confidence (last columns). All TOSTs indicate that all proportions of interventions are within the 20-30% interval.

	Humans	GPT 4.1 mini	GPT 5.1 mini	GPT 5.2	Claude Haiku 4.5
GPT 4.1 mini	0.80				
GPT 5.1 mini	0.70	0.85			
GPT 5.2	0.78	0.89	0.84		
Claude Haiku 4.5	0.84	0.86	0.81	0.86	
Claude Sonnet 4.6	0.83	0.88	0.80	0.86	0.86

Table S4: Pearson correlation coefficient between Fermi ratings produced by five different large language models (LLMs). All comparisons are statistically significant with $p<0.001$.

	β	SE	t	p-val	95% C.I.
Intercept	0.20	0.02	8.00	< 0.001	[0.15 ; 0.24]
Fermi Ratings	-0.01	0.003	-2.98	0.003	[-0.02 ; -0.003]
DF	323				
Log-likelihood	107				
AIC	-206				
BIC	-191				

Table S5: Replication of the main result of Study 1 without normalization. Generalized linear mixed-effect model of Collective Error, with the Fermi Ratings as a fixed effect, and random intercepts for each question.

	Study 1			Study 2			Study 3		
	Error	PD	GE	Error	PD	GE	Error	PD	GE
GPT 4.1 mini	-0.20 [-0.30, -0.09]	0.38 [0.30, 0.46]	0.13 [0.04, 0.23] p = 0.005	-0.21 [-0.37, -0.04] p = 0.017	0.39 [0.26, 0.51]	0.33 [0.20, 0.46]	-0.24 [-0.35, -0.13]	0.48 [0.40, 0.56]	0.13 [0.03, 0.23] p = 0.011
GPT 5.1 mini	-0.22 [-0.32, -0.11]	0.39 [0.30, 0.46]	0.19 [0.09, 0.28]	-0.28 [-0.43, -0.11] p = 0.001	0.44 [0.31, 0.55]	0.32 [0.18, 0.45]	-0.30 [-0.40, -0.19]	0.45 [0.36, 0.53]	0.21 [0.11, 0.31]
GPT 5.2	-0.20 [-0.30, -0.09]	0.45 [0.37, 0.52]	0.12 [0.02, 0.21] p = 0.017	-0.28 [-0.43, -0.11] p = 0.002	0.45 [0.32, 0.56]	0.30 [0.16, 0.43]	-0.27 [-0.37, -0.16]	0.50 [0.42, 0.57]	0.11 [0.00, 0.21] p = 0.045
Claude Haiku 4.5	-0.25 [-0.35, -0.15]	0.35 [0.27, 0.43]	0.12 [0.03, 0.22] p = 0.011	-0.31 [-0.46, -0.15]	0.40 [0.27, 0.52]	0.35 [0.22, 0.48]	-0.31 [-0.41, -0.20]	0.35 [0.26, 0.44]	0.15 [0.05, 0.25] p = 0.004
Claude Sonnet 4.6	-0.22 [-0.32, -0.11]	0.39 [0.31, 0.47]	0.15 [0.05, 0.24] p = 0.003	-0.29 [-0.44, -0.12]	0.46 [0.33, 0.57]	0.34 [0.20, 0.46]	-0.32 [-0.42, -0.21]	0.30 [0.21, 0.39]	0.05 [−0.06,0.15] p = 0.359

Table S6: Full statistics for LLM-based Fermi ratings (Table 1, main text). For each LLM (rows) per variable per study, we report the Pearson correlation coefficient r, the 95% confidence interval (Fisher z-transform), and the exact two-sided p value. All $p < 0.001$ unless reported otherwise.

Study 1 (N=500)

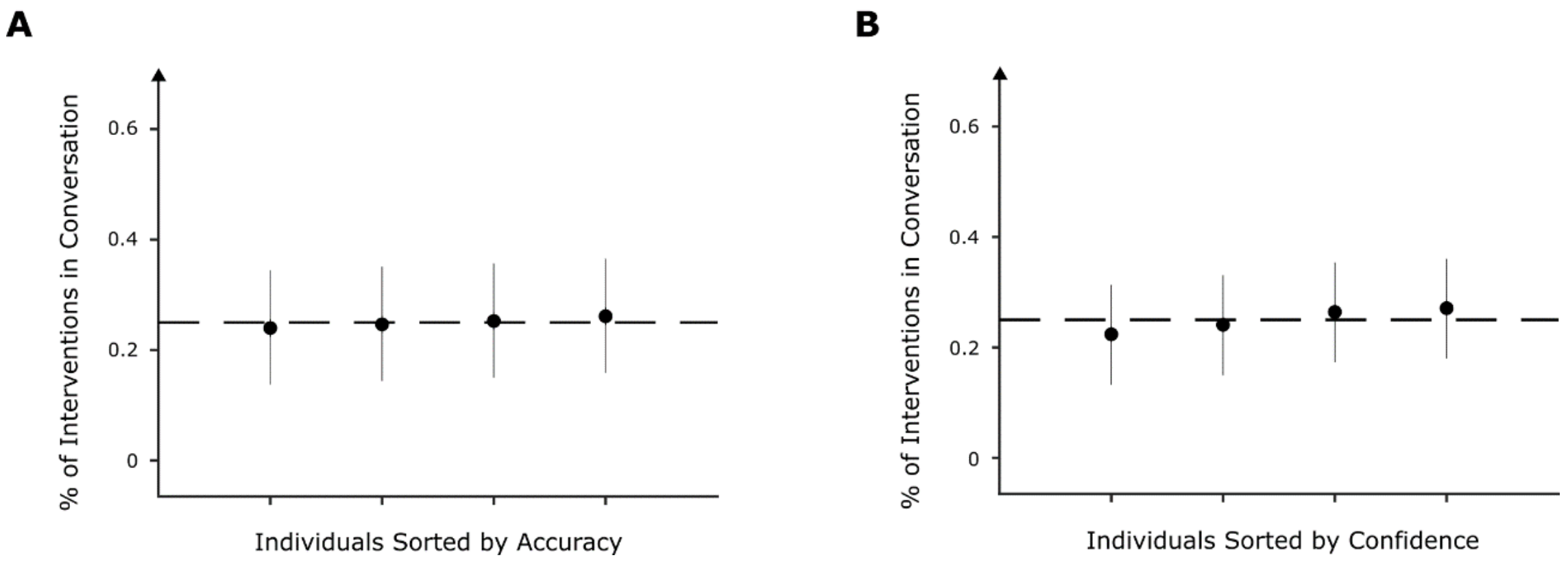

Fig. S1. Accuracy and confidence for each individual for Experiment 1. (A) Percentage of interventions (messages) of each individual for each conversation. Individuals are sorted by accuracy. The full lines represent the standard deviation of the mean, and the dotted line represents 25% **(B)** Percentage of interventions (messages) of each individual for each conversation. Individuals are sorted by confidence. The full lines represent the standard deviation of the mean, and the dotted line represents 25%.

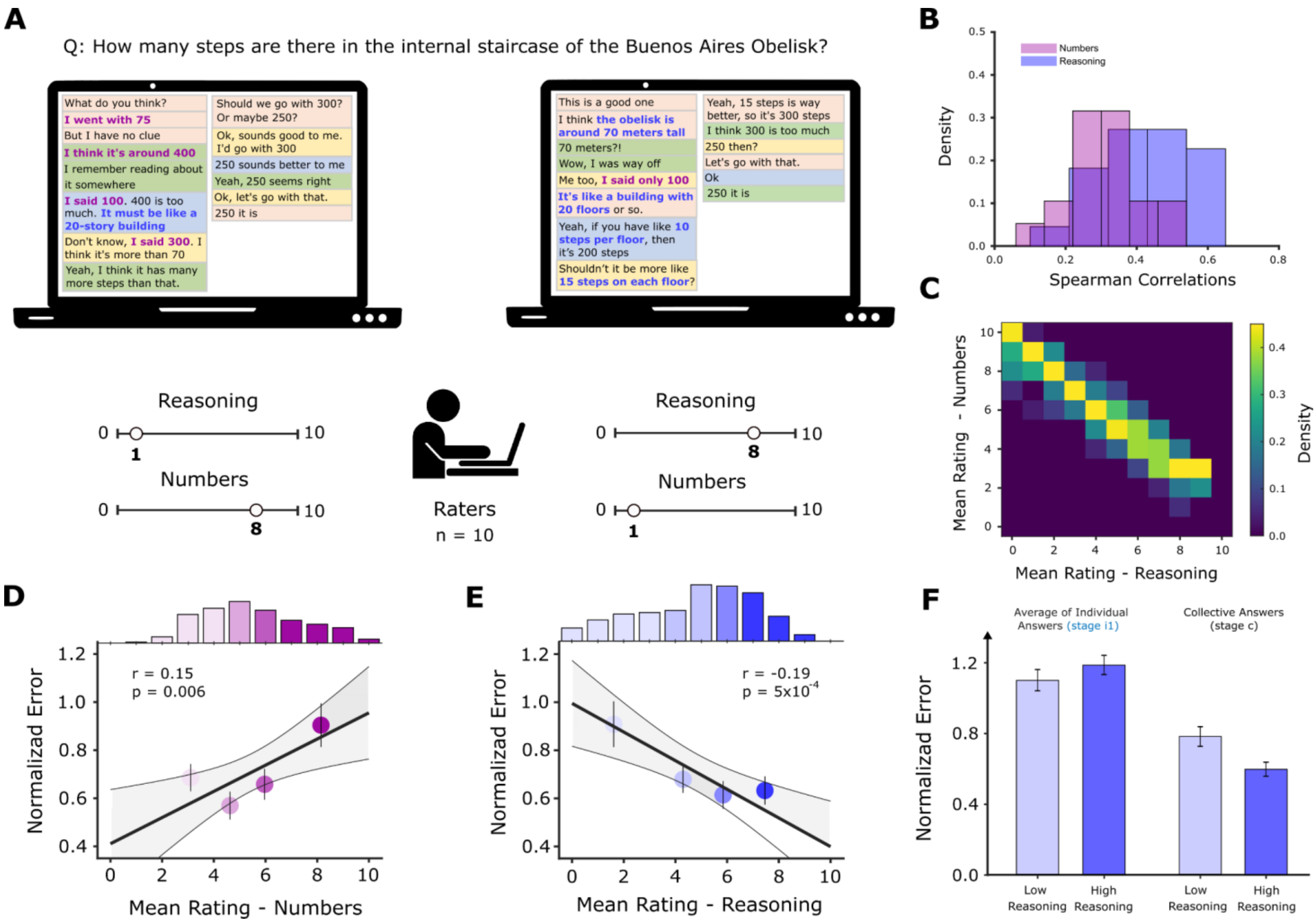

Fig. S2. Analysis of Conversations. Replication of Figure 2 under a different way of coding conversations, (A) Example of conversations that lead to opposing ratings of the strategies used in them. 6 raters were trained on how to classify conversations in terms of whether participants pooled their previous individual answers (Numbers strategy) or reached a new collective answer by employing a reasoning procedure (Reasoning strategy). Additional icons made by Freepik and Maxim Basinski Premium from www.flaticon.com. **(B)** Distribution of correlations between the values provided by each rater with each other, for each strategy. The correlation of the values provided by different raters is generally high, for both strategies. **(C)** Density of ratings for the Reasoning and Numbers Strategies. Ratings for both strategies were negatively correlated: whenever raters on average assigned a high value for the Reasoning strategy in a conversation, they also tended to assign on average a very low value for the Numbers strategy to that conversation. **(D)** Normalized error as a function of the Numbers strategy ratings (averaged across raters). We plot the quartiles of the distribution of normalized errors, along with the best linear fit of the data, and the 95% confidence intervals. The bars above the main graph show the actual distribution of normalized errors. We also show the Pearson correlation coefficient, and the corresponding p-value **(E)** Normalized error as a function of the Reasoning strategy ratings

(averaged across raters). We plot the quartiles of the distribution of normalized errors, along with the best linear fit of the data, and the 95% confidence intervals. The bars above the main graph show the actual distribution of normalized errors. We also show the Pearson correlation coefficient, and the corresponding p-value. **(F)** Variation of Normalized Error between groups and averages of the individual answers. We categorize a group for each conversation as either low Reasoning (when the Reasoning rating was lower than the Numbers rating) or high Reasoning (when the Reasoning rating was higher than the Numbers rating).

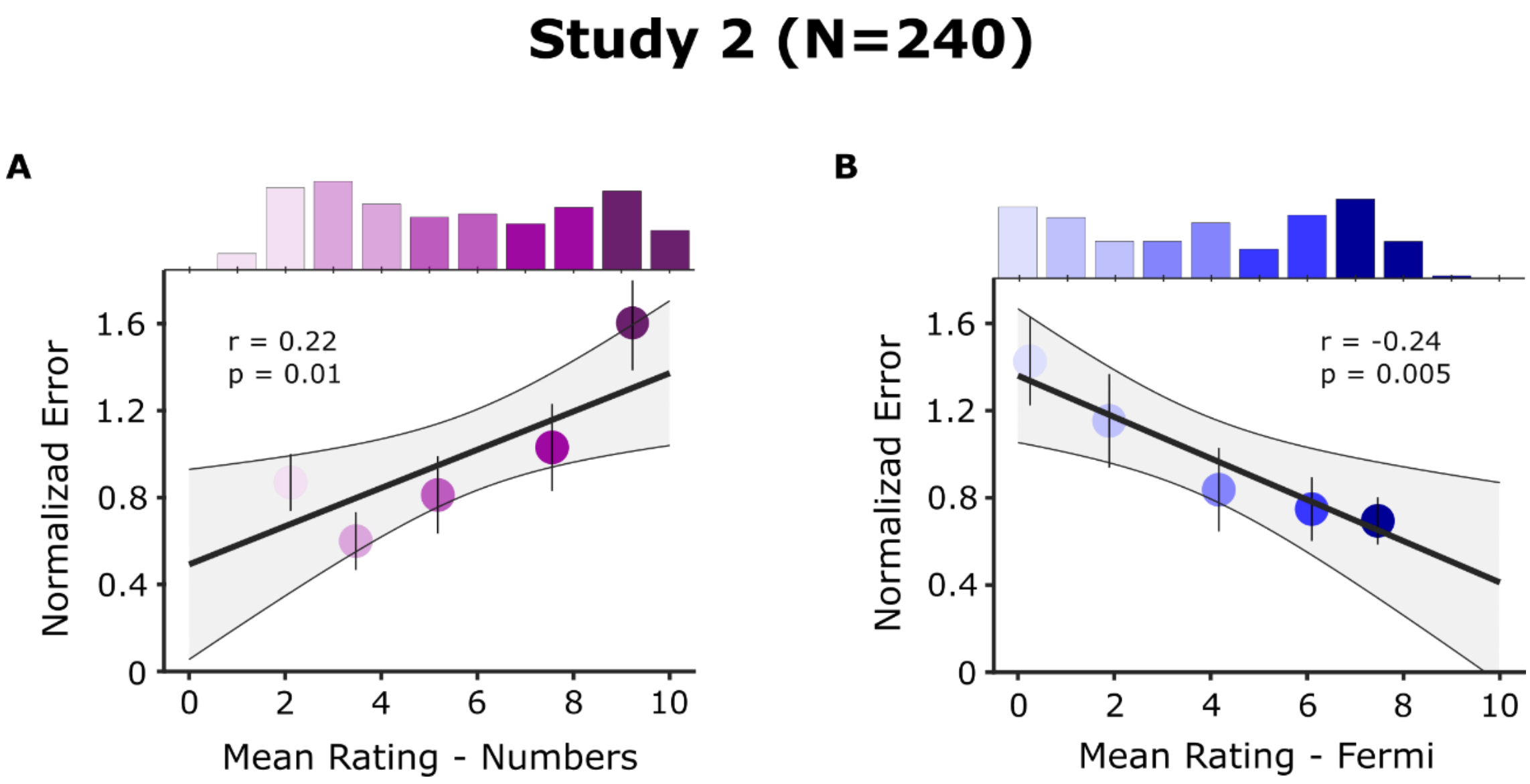

Fig. S3. Normalized error as a function of human ratings. Replication of Figs 2D and 2E (Study 1) in Study 2. (A) Normalized error as a function of the Numbers strategy ratings (averaged across raters). We plot the quintiles of the distribution of normalized errors, along with the best linear fit of the data, and the 95% confidence intervals. The bars above the main graph show the actual distribution of normalized errors. We also show the Pearson correlation coefficient, and the corresponding p-value **(B)** Normalized error as a function of the Fermi strategy ratings (averaged across raters). We plot the quintiles of the distribution of normalized errors, along with the best linear fit of the data, and the 95% confidence intervals. The bars above the main graph show the actual distribution of normalized errors. We also show the Pearson correlation coefficient, and the corresponding p-value

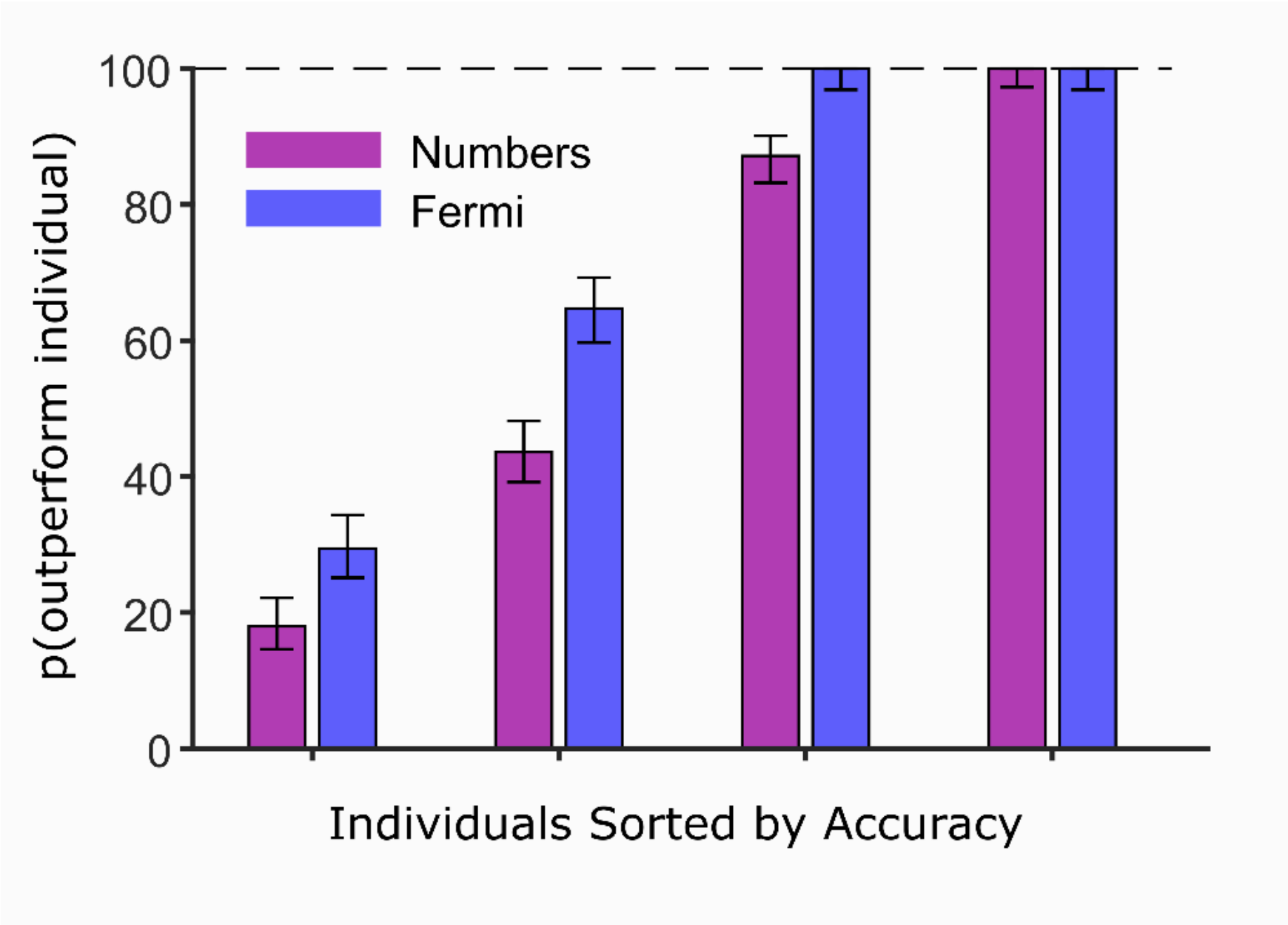

Fig. S4. Probability that the collective estimate outperforms individual estimates. In Study 2, **i**ndividuals within each group were ranked according to their individual estimation error (from most to least accurate). The figure shows the probability that the collective estimate outperforms the estimate of each individual in the group under the two experimental conditions (Numbers and Fermi). Error bars represent ±1 s.e.p.

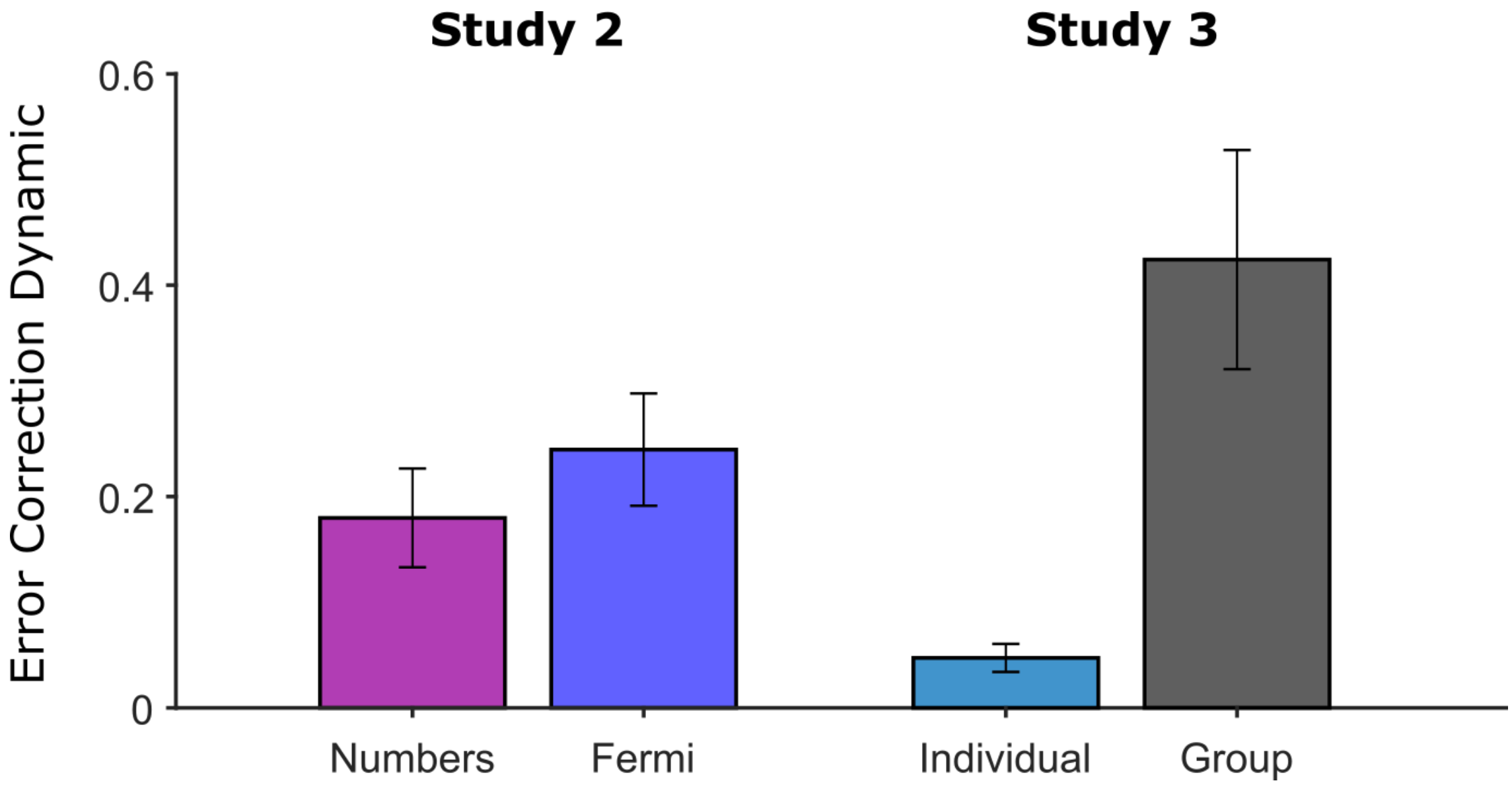

Fig. S5. Error correction dynamics in Studies 2 and 3. In Study 2, the presence of linguistic markers reflecting error correction dynamics (e.g., "too little", "too much", "better") was not statistically different between conditions involving different instructions. In Study 3, the same linguistic maker led to a pronounced and significant different between the individual and collective condition. Bars show the average value of the metric for each study and condition ±1 s.e.m.

Supplementary Note 1

The instructions provided to the raters were the following:
Your task is to read conversations between groups of four individuals discussing factual topics (e.g., "What is the height of the Eiffel Tower?"). Each group was instructed to "try to reach consensus" on these topics. There are eight different questions in total.

All participants first completed an individual stage, where they provided answers to the eight questions on their own. Afterward, they were divided into groups and asked to discuss 4 of the 8 questions, with five minutes allotted per question (they could see their own previous answers). Most groups did reach consensus, but this was not required.

As you read each conversation, you will be asked to rate, on a scale from 0 (completely absent) to 10 (completely present), the extent to which two possible strategies for reaching consensus were used.

The first strategy involves combining the estimates from the initial individual stage. This could mean calculating an average, weighting responses by confidence, discarding some answers in favor of others, or any other approach that makes use of their original estimates. We call this the "Numbers Strategy".

The second strategy involves breaking down the problem into smaller parts, estimating those quantities, and then combining them to arrive at a final answer. For example, for the question "What is the height of the Eiffel Tower?", one might assume the Tower is similar to a 100-story building (first estimate), and if each story is 3 meters tall (second estimate), then the Tower would be about 300 meters high (which in this case happens to be correct, though this is not always so). We call this the "Fermi Strategy".

Thus, for each conversation, you will be asked to answer two questions:

a) Did they reach consensus by combining their initial estimates from the first stage? (Numbers Strategy) / 0 = completely absent, 10 = completely present

b) Did they reach consensus by breaking the problem into smaller parts? (Fermi Strategy) / 0 = completely absent, 10 = completely present

Note that the ratings for a) and b) do not need to sum to 10. A group may have used both strategies, or neither. Also, be sure to use the full scale rather than only the extremes (0 and 10).
