# Supplementary material for: Collective problem decomposition improves the wisdom of deliberative crowds
Source: Nat Commun. 2026 Aug 5;17:9431. doi: 10.1038/s41467-026-76365-y (PMC13538491; doi:10.1038/s41467-026-76365-y)
Supplement: Supplementary file 2 [file 41467_2026_76365_MOESM2_ESM.pdf]

nature portfolio

Corresponding author(s): Joaquín Navajas
Last updated by author(s): May 22, 2026

Reporting Summary

Nature Portfolio wishes to improve the reproducibility of the work that we publish. This form provides structure for consistency and transparency in reporting. For further information on Nature Portfolio policies, see our Editorial Policies and the Editorial Policy Checklist.

Statistics

For all statistical analyses, confirm that the following items are present in the figure legend, table legend, main text, or Methods section.

n/a	Confirmed	
☐	☒	The exact sample size (*n*) for each experimental group/condition, given as a discrete number and unit of measurement
☐	☒	A statement on whether measurements were taken from distinct samples or whether the same sample was measured repeatedly
☐	☒	The statistical test(s) used AND whether they are one- or two-sided<br>*Only common tests should be described solely by name; describe more complex techniques in the Methods section.*
☐	☒	A description of all covariates tested
☐	☒	A description of any assumptions or corrections, such as tests of normality and adjustment for multiple comparisons
☐	☒	A full description of the statistical parameters including central tendency (e.g. means) or other basic estimates (e.g. regression coefficient) AND variation (e.g. standard deviation) or associated estimates of uncertainty (e.g. confidence intervals)
☐	☒	For null hypothesis testing, the test statistic (e.g. *F*, *t*, *r*) with confidence intervals, effect sizes, degrees of freedom and *P* value noted<br>*Give P values as exact values whenever suitable.*
☐	☒	For Bayesian analysis, information on the choice of priors and Markov chain Monte Carlo settings
☐	☒	For hierarchical and complex designs, identification of the appropriate level for tests and full reporting of outcomes
☐	☒	Estimates of effect sizes (e.g. Cohen's *d*, Pearson's *r*), indicating how they were calculated

Our web collection on statistics for biologists contains articles on many of the points above.

Software and code

Policy information about availability of computer code

Data collection	The code for the web platform we used for data collection can be found at: https://github.com/facuzeta/chat-room-platform
Data analysis	The data (both raw and processed) supporting our analyses are available at the Open Science Framework (https://doi.org/10.17605/OSF.IO/BRU7K). We used the MATLAB programming language for all data analyses, and the Python programming language for some aspects of data processing.

For manuscripts utilizing custom algorithms or software that are central to the research but not yet described in published literature, software must be made available to editors and reviewers. We strongly encourage code deposition in a community repository (e.g. GitHub). See the Nature Portfolio guidelines for submitting code & software for further information.

Data

Policy information about availability of data

All manuscripts must include a data availability statement. This statement should provide the following information, where applicable:

- Accession codes, unique identifiers, or web links for publicly available datasets
- A description of any restrictions on data availability
- For clinical datasets or third party data, please ensure that the statement adheres to our policy

The data (both raw and processed) and code supporting our analyses are available at the Open Science Framework (https://doi.org/10.17605/OSF.IO/BRU7K). The chatroom platform code is available at https://github.com/facuzeta/chat-room-platform.

Research involving human participants, their data, or biological material

Policy information about studies with human participants or human data. See also policy information about sex, gender (identity/presentation), and sexual orientation and race, ethnicity and racism.

Reporting on sex and gender	We asked participants to report their gender (male, female, or other). Throughout our studies and analyses, we did not find any significant differences between genders on any task. We report the proportion of female participants for every study. Data is anonymized, so each participant has a specific code, and we have the answer for the self-reported gender associated with each code (available at the Open Science Framework: https://osf.io/bru7k/?view_only=20b5fd8f315b4cb083186aeba294b632). We did not expect to find any differences related to this. We did not ask participants to report their sex.
Reporting on race, ethnicity, or other socially relevant groupings	We have not used these constructs or classified our participants in any such way, nor have we asked participants any question related to these constructs.
Population characteristics	Study 1: 500 participants (288 female, mean age 25.4 yr, s.d. 7.7 yr)<br>Study 2: 240 participants (148 female, mean age 30.6 yr, s.d. 9.8 yr)<br>Study 3: 160 participants (95 female, mean age 29.8 yr, s.d. 10.9 yr)
Recruitment	Participants for our studies were recruited using non-probabilistic convenience and snowball sampling. Recruitment was conducted through three strategies: (1) emails sent by Universidad Torcuato Di Tella (UTDT) to its own students, (2) a subject database at the Laboratorio de Neurociencia at UTDT, and (3) invitations shared on social media (e.g., Instagram, Twitter, Facebook) through the private accounts of the researchers in charge of this project
Ethics oversight	All protocols were approved by the ethics committee at Universidad Abierta Interamericana (Buenos Aires, Argentina), protocol 0-1108

Note that full information on the approval of the study protocol must also be provided in the manuscript.

Field-specific reporting

Please select the one below that is the best fit for your research. If you are not sure, read the appropriate sections before making your selection.

☐ Life sciences ☒ Behavioural & social sciences ☐ Ecological, evolutionary & environmental sciences

For a reference copy of the document with all sections, see nature.com/documents/nr-reporting-summary-flat.pdf

Behavioural & social sciences study design

All studies must disclose on these points even when the disclosure is negative.

Study description	We present three quantitative studies. The first one was an observational study, and the other two were preregistered, experimental studies.
Research sample	We collected data from 900 individuals, all residents of Argentina, across three samples: Study 1 involved 500 participants (288 female, mean age 25.4 yr, s.d. 7.7 yr), in Study 2 we recruited 240 participants (148 female, mean age 30.6 yr, s.d. 9.8 yr), and Study 3 involved 160 participants (95 female, mean age 29.8 yr, s.d. 10.9 yr). Participants for our studies were recruited using non-probabilistic convenience and snowball sampling. The sample is a convenience sample and is not statistically representative of the general population of Argentina.
Sampling strategy	Participants for our studies were recruited using non-probabilistic convenience and snowball sampling. Recruitment was conducted through three strategies: (1) emails sent by Universidad Torcuato Di Tella (UTDT) to its own students, (2) a subject database at the Laboratorio de Neurociencia at UTDT, and (3) invitations shared on social media (e.g., Instagram, Twitter, Facebook) through the private accounts of the researchers in charge of this project The first study was aimed at replicating the results from Navajas et al., Nat. Hum. Behav., 2018 in chatrooms (a very different setting). The second and third studies were preregistered, and power-analysis employing the data from the first study was used to assess their required sample sizes. Our research questions concern general mechanisms of collective deliberation and reasoning rather than population-level prevalence estimates, and therefore do not require a population-representative sample. The chosen sample is consistent with prior work in this research line (Navajas et al., Nat. Hum. Behav., 2018; Navajas et al., Roy. Soc. Op. Sci., 2022), and the recruitment channels were selected for feasibility of large-scale online data collection. Self-selection bias from our sampling method is unlikely to qualitatively alter the direction or relative magnitude of the within-participant or between-condition effects reported here, which depend on general cognitive mechanisms of collective deliberation. Consistent with this, our key findings replicate patterns reported in prior work using overlapping but non-identical samples (Navajas et al., 2018).
Data collection	All studies in this paper were online studies, performed through a platform designed specifically for this purpose. Participants were divided into groups, and could interact with other group members through an online chatroom. We previously contacted participants through email, and provided them with instructions. Participant assignment into each group was handled by the platform, which was used by people hired for that task (and who were blind to the hypotheses of the studies). For studies 2 and 3 (experimental), the assignment to each condition was random and automatic.

Timing	Study 1: Start date: 18/11/2023. Stop date: 18/12/2023.<br>Study 2: Start date: 10/02/2024. Stop date: 10/03/2024.<br>Study 3: Start date: 15/04/2024. Stop date: 15/05/2024.
Data exclusions	Exclusion criteria were pre-registered for Studies 2 and 3 (aspredicted.org/TL2_Q1Q and aspredicted.org/SZ5_MNP); for Study 1, we followed the procedure implemented in previous research (Navajas et al., Nat. Hum. Behav., 2018; Navajas et al., Roy. Soc. Op. Sci., 2022). All studies exclude data using two criteria: 1) estimates that deviate more than 2.5 MADs from the median distribution in stage 1, and 2) groups with at least two participants leaving the chatroom before the end of stage 2. This did not lead to participant dropout, given that it happened on less than 1% of all estimation questions.
Non-participation	No participants declined invitation/participation. 19 participants in Study 1 dropped out during the study (due to connection failure or platform error). No participants dropped out in the other studies.
Randomization	Allocation into groups was randomized, and automatic (performed by the platform). Questions were presented in random order. Which questions were discussed in groups was also randomized for each group. For Studies 2 and 3, which group would belong to which condition was also randomized.

Reporting for specific materials, systems and methods

We require information from authors about some types of materials, experimental systems and methods used in many studies. Here, indicate whether each material, system or method listed is relevant to your study. If you are not sure if a list item applies to your research, read the appropriate section before selecting a response.

Materials & experimental systems

n/a	Involved in the study
☒	☐ Antibodies
☒	☐ Eukaryotic cell lines
☒	☐ Palaeontology and archaeology
☒	☐ Animals and other organisms
☒	☐ Clinical data
☒	☐ Dual use research of concern
☒	☐ Plants

Methods

n/a	Involved in the study
☒	☐ ChIP-seq
☒	☐ Flow cytometry
☒	☐ MRI-based neuroimaging

Plants

Seed stocks	*Report on the source of all seed stocks or other plant material used. If applicable, state the seed stock centre and catalogue number. If plant specimens were collected from the field, describe the collection location, date and sampling procedures.*
Novel plant genotypes	*Describe the methods by which all novel plant genotypes were produced. This includes those generated by transgenic approaches, gene editing, chemical/radiation-based mutagenesis and hybridization. For transgenic lines, describe the transformation method, the number of independent lines analyzed and the generation upon which experiments were performed. For gene-edited lines, describe the editor used, the endogenous sequence targeted for editing, the targeting guide RNA sequence (if applicable) and how the editor was applied.*
Authentication	*Describe any authentication procedures for each seed stock used or novel genotype generated. Describe any experiments used to assess the effect of a mutation and, where applicable, how potential secondary effects (e.g. second site T-DNA insertions, mosiacism, off-target gene editing) were examined.*
