# Supplementary material for: Collective problem decomposition improves the wisdom of deliberative crowds
Source: Nat Commun. 2026 Aug 5;17:9431. doi: 10.1038/s41467-026-76365-y (PMC13538491; doi:10.1038/s41467-026-76365-y)
Supplement: Supplementary file 3 [file 41467_2026_76365_MOESM3_ESM.pdf]

nature portfolio

Peer Review File

Collective problem decomposition improves the wisdom of deliberative crowds

Corresponding Author: Professor Joaquin Navajas

This file contains all reviewer reports in order by version, followed by all author rebuttals in order by version.

Version 0:

Reviewer comments:

Reviewer #1

(Remarks to the Author)
In this ms., the Authors study the mechanisms by which groups discussing with each other reach answers that improve on other aggregation procedures such as averaging. In particular, the Authors argue that during discussion, some groups unpack the problem at hand (here, providing numerical estimates) into various dimensions that they estimate independently, before combining them to yield a better estimate. The Authors show that this process is important in three studies, which show (i) that groups that happen to use that decomposition technique more improve more (ii) that teaching groups to do it allows them to improve more and (iii) that teaching individuals to do it doesn't yield the same improvement in performance.

This is a very strong ms., with a solid introduction, good experiments, a sensible discussion, so I have no major comments, and I would recommend accepting with (very) minor revisions.

Minor comments:

How would the process described here extend to other problems, besides numerical problems? The Authors mention categorical problems as the other category, but they are not (by far) the only two possibilities. People might have to come up with a plan, with an explanation, with a piece of advice, with the analysis of a situation, etc. The fact that mathematical aggregation techniques don't really apply there doesn't mean that this isn't most of what groups are asked to do. The Authors should, I think, (i) mention this as a limitation of their work (i.e. they focus on a narrow type of problems); (ii) speculate as to how decomposition could apply in other cases.

Question: do the groups that use decomposition take more time? It should be the case, in that people just telling each other what they think the answer is is very quick, but is it? If yes, is that a drawback (e.g. could the Authors measure improvement as a function of time spent)? If not, why not? (e.g. because the instructions or the experimental set up make it natural for all groups to spend about as much time on the task?)

I don't really get that bit "Moreover, the Similarity index was negatively associated with estimation error (Fig. 3B; Pearson $r = –0.15$, $p = 0.007$), and this effect was significantly mediated by "Fermi" ratings (Fig. 3C; total effect = $–0.11 \pm 0.03$, $p = 0.006$; direct effect = $–0.08 \pm 0.04$, $p = 0.02$; indirect effect = $–0.03 \pm 0.01$, $p = 0.009$; see Methods for details)."

I find it weird that the Authors talk about 'discussed questions' in the individual condition of Study 3, since these people didn't discuss with anyone, right? Do the Authors mean something like 'reflected on'? Should they use 'deliberated' (instead of 'discussed') throughout, as it can apply both to group and to individual contexts?

Have the Authors analyzed how exactly groups who use decomposition improve? Does it tend to improve on the best estimate already present in the group, or does it mostly bring the outliers towards the average? (and which of these two does it do more than just sharing one's opinion?)

Finally, could the Authors say a bit more about why they think decomposition works better in groups? Is it because different people are better at estimating different elements? Or because groups just do more decomposition than individuals, even

when instructed?

(Remarks on code availability)

Reviewer #2

(Remarks to the Author)
Review of “Collective problem decomposition drives the wisdom of deliberative crowds”

There is much to like in this paper. The core idea is interesting. However, I found the NLP related methods to be considerably limited and lacking, and ultimately, I did not find that a mechanistic analysis of the main effect was clearly articulated or supported. This stems, in my view, from a lack of a clear definition and operationalization of “problem decomposition”. That said, I do think it may be possible to address these concerns in a revision. At this stage, I'll focus my comments on my major concerns.

1. Unclear/Absence of Mechanism.

The authors argue that problem decomposition mostly only benefits collective deliberation but not independent, individual reasoning, though no mechanistic explanation of why problem decomposition only benefits collectives is provided, despite it being the main finding. If problem decomposition is a superior reasoning strategy, it should benefit individuals as well (which they only find weak support for in study 2). This leads me to wonder whether PD is really driving the effect or some other known factor in collective intelligence that PD indirectly activates but is not directly responsible for.

For example, consider the robust finding that the wisdom of the crowds and collective estimation benefits from cognitive diversity (e.g., the diversity-prediction theorem and Page's work). It could simply be the priming people to only share numbers restricts available diversity only to numbers, whereas priming them to PD introduces more conceptual diversity by getting people to share the differing perspectives (reasons) underlying their numbers. People with the same estimates, after all, can arrive at these estimates for different reasons. Encouraging the exchange of reasons (or, in other words, allowing conversation to not be arbitrarily restricted to number exchanges) creates more surface area for conceptual diversity to link into the conversation beyond just the numbers. Measuring the raw number of words in the chat is not a good measure for this because it's incredibly coarse. What matters are more substantive differences in the breadth of reasoning engaged. This is where a measure of information density at the language level is likely to be much more appropriate (see reference from Aceves & Evans 2024 below, which provides one among several possible measures of information density). But note that if this argument is supported, it's not clear that the effect is driven by PD specifically, but rather than the PD condition simply allows more information diversity on top of number exchange. This concern connects with my doubts that the linguistic measures of PD proposed are actually measuring what the authors think they are measuring.

• Aceves, P., Evans, J.A. Human languages with greater information density have higher communication speed but lower conversation breadth. Nat Hum Behav 8, 644–656 (2024). https://doi.org/10.1038/s41562-024-01815-w

Another possible explanation is that the effects of PD depend on deliberation not because of PD specifically, but rather because priming for the exchange of reasons increases coordination. It's not simply that people share reasons (on top of numbers), but that they are tasked with coordinating their reasons and their numbers. It could be that inducing coordination of reasons alone improves performance, regardless of whether these reasons engage in a specific strategy called PD. This is important because the linguistic measures of the authors do not, in my opinion, clearly establish that their treatment condition induced PD specifically, due in part to the vagueness of their measure of PD (described in more detail below). One possible way to measure this would be to test whether, over time, participants in the PD condition exhibit greater increases in the similarity of their language/framings, in comparison to just those in the numbers condition. It could be that PD simply increases available information and induces more coordination/alignment over time, both of which are known vectors for improving collective intelligence. While this would still be interesting as a finding for establishing the value of deliberation, it's less novel and wouldn't necessarily support PD specifically as the mechanism (since, as factors, they are not explanations of why PD works, but rather mechanisms that are more general than PD and do not depend on PD specifically). More needs to be done to confirm that PD specifically is at a play and to explain why PD's benefits yield mainly in collective rather than individual contexts.

2. Unclear definition/operationalization/measurement of problem decomposition (PD).

The authors' definition of collective fermi estimation is vague and includes several different processes, explicitly referencing both ‘rough approximations’ and ‘problem decomposition’ (which are quite distinct and can exist without each other), while also implicitly describing this process as increasing ‘reasoning’ (which is much more general than PD specifically and can involve all kinds of processes, from argumentation to counterfactual analysis). This vagueness makes it really unclear how it can be effectively measured. This is more concerning when considering that the main measures the authors provide are very basic and not directly related to any of the processes above, but instead are a loose proxy that is supported by correlations of

varying strength. Specifically, they attempt to proxy PD by measuring the semantic relatedness of language in the conversation to the initial estimation task phase. Not only is this measure unrelated to reasoning directly (all kinds of language can satisfy this measure but be unrelated to reasoning, let alone PD specifically), but this measure is also likely confounded with the task design, because those in the number sharing condition are asked to focus on sharing numbers, which will reduce the overall language used and impact this similarity metric but for trivial reasons.

In sum, this is not a convincing measure of reasoning, let alone a specific reasoning process like PD. It can and should be significantly improved. The human annotation classifications (and their correlations with this measure) are unsatisfying. This is partly because the framing of the classification is loaded to begin with. Annotators are told to assume chats fall along the framings of the conditions (i.e., as either being focused on numbers or on PD), but there are certainly many other possible classifications here, such that these distinctions may not capture the underlying variation of interest. The authors report a robustness test that their annotation results hold if people compare chats as being either number focused or focused on reasoning generally, but this actually reinforces my concern – the fact that this rating is highly correlated with the rating focusing on PD suggests it's by no means capturing PD specifically, and reasoning is a catch-all-term for such a wide variety of things. As a result, I'm not clear on what the ratings of a dozen or so students using is capturing and how this relates to a clear mechanistic picture.

This concern was reinforced when I saw the figure 2. The example of PD given by the authors is a case where a participant breaks down the problem by talking about how many floors are in the building and estimating these components. Yet, in both of the examples shown in figure 2, someone mentions/inquiries into how many floors are in the building. The difference is that in the PD case, people focus on this and elaborate, whereas in the numbers case, they ignore this and focus on numbers. But some form of PD is happening in both cases (i.e., someone has shared an argument along these lines). It's not clear to me why the first case should be listed as PD = 1 whereas the latter as PD = 8 where in both cases one of the messages (out of only a dozen) mentions exactly the strategy described as PD. While I appreciate the correlations the authors provided to support the annotator judgments, this led me to wonder about variance. How much variance is there at the individual chat level, and what are the chats associated with the highest level of variance in people's use of this coding scheme? Or, in other words, if we look only at the chats in the by number condition, is there a correlation with the presence of PD and performance? It seems that clearly some of the chats in this condition have higher PD than others. As well, a valuable robustness test would be to test whether the main results hold when only comparing the number and PD ratings with the lowest variance cut off by some threshold (like quartile ranges etc.) to make sure the comparisons are really driven by the clear cut cases that capture the differences that are supposed to make a difference here.

I will say though that as much as these robustness tests and clarifications will be helpful, I don't think these alone will address the first order problem of collective fermi estimation being vaguely defined and operationalized in an unclear way with respect to the linguistic measures.

(Remarks on code availability)

Reviewer #3

(Remarks to the Author)
This paper reports a series of 3 experiments to test whether problem decomposition ("fermi estimates") improve estimate accuracy on numeric trivia questions. Each experiment follows a similar protocol: (1) individuals answer 8 numeric trivia questions, (2) individuals discuss a random subset of 4 questions in groups of four, (3) individuals answer all 8 questions again. The primary focus of interest is the extent to which individuals decompose the numeric estimates into sub-estimates to be recombined into a final answer.

The first experiment is exploratory (not pre-registered) and analyzes endogenous variation in decomposition by rating each conversation on the extent to which decomposition occurs. The second experiment is a pre-registered confirmatory analysis in which the experimenters randomly allocate groups to either a condition which explicitly instructs them to decompose the estimates, or a control condition.

Besides my comments below, this study reports clear, strong results with replicable and straightforward methods. Study 3 in particular provides a clean comparison of independent individuals versus collaborating groups. If the authors can clarify my concern about the possibility of looking answers up online, it will represent a very strong contribution. Were it not for that (presumably easily addressed) concern, I would say this paper could be published as-is. Nonetheless, I offer a number of major and minor comments aimed at improving the manuscript, in no particular order.

Major Comments
- Generalizability. I realise that you don't have data to address the question of "when will fermi estimates improve outcomes" but perhaps you can offer some brief discussion on this point. Presumably, you picked questions for their decomposibility—perhaps you could tell us how you chose the questions.
- Mechanisms. Your deliberation condition confounds collective deliberation with individual deliberation, i.e., you can't tell whether the improved accuracy from group interaction is due to the group interaction per se, or the fact that individuals are improving of their own accord. Although you randomize groups to discuss 4 questions and leave 4 questions undiscussed (a nice design) this still yields huge differences in attention-per-question for discussed and undiscussed. On average, each

person can give attention worth about 75 seconds to each undiscussed question (5+5=10 minutes, divided by 8 questions) versus about 150 seconds for discussed questions (75 seconds + 5 minutes divided by 4 questions). And so, your primary design conflates interaction between people with individual attention. As your primary focus is the comparison of fermi with non-Fermi conditions I don't see this as a fatal flaw of the paper, but you should be more circumscribed about your results. Your results indeed replicate prior findings that deliberation in small groups does not HARM the wisdom of crowds, but you can't say that deliberation groups yield more accurate answers than people working alone, for a comparable amount of time. The only place you really do get this comparison is in the very last result reported, comparing Figs 5C and 5D—which clearly shows that people interacting with other people produces more improvement than people working alone.
- Exogeneous improvements. Please tell us more about the setting in which participants conducted the study, and what information you have about their ability to look up answers to these questions online. Notably, these questions are particularly easy to find on google—the number of episodes of 'Friends' is a fact about one of the most popular television shows of all time. If they were able to search for answers, this possibility interacts with group deliberation in ways that are uninteresting: a group collaborating can divide-and-conquer (i.e. assign different questions to search) whereas a collection of independent individuals faces some redundancy. E.g., was this study conducted in a physical lab setting that prevented participants from searching online for answers?
- Title. Although it's not a breaking issue, I take some issue with the title. You don't show that collective problem decomposition "drives" the wisdom of deliberative crowds, you show that it improves the wisdom of crowds. You also show that collaborative decomposition is more effective than individual decomposition. Clearly, it is true also both that the wisdom of crowds still occurs without decomposition and that decomposition improves outcomes for individuals working alone (5D).

Minor Comments
- p.18, you don't report statistical significance or effect size for the improvements that result from individual/independent fermi decomposition.
- Some revision of terminology might help. I recognize these terms are used ambiguously in lay language, so you'll need to figure out a consistent but clear parlance. You use the term "deliberation" to refer to group discussion, which is confusing in this case because you also allow for individual deliberation i.e. careful thought. You use the term "discussed" to refer to individual deliberation (e.g. p.18) which is just confusing. I realize that the individuals are "discussing" with the empty chat interface but it's not really quite the same, I think. Perhaps you can find words that are explicitly group-restrictive to refer to peer-to-peer discussion, and explicitly intelligence-restrictive to refer to careful thinking.
- For study 1, please explicitly state your prior for the Bayesian analysis, so we can follow your analysis without downloading the code. For study 3, you refer us to the methods for explanation of the generalized linear effects model, but then we have to look online to find out the model. If word limit allows, please give us basic details of the analysis right in the paper.
- There are some minor deviations from the pre-registration. I don't see these as problematic (e.g. swapping a non-parametric test for a parametric test) but it is worth explaining why you made these decisions. If I'm interpreting correctly, it looks like you switched from reporting Stage i2/stage 3 error as DV, to change-in-error as a DV, which is a more powerful test. Please report all pre-registered analyses or deviations therefrom, with explanation. You also introduce some additional analyses—please label non-pre-registered analyses as "exploratory."
- When you report confidence intervals, please indicate that they are (presumably) 95% intervals.

(Remarks on code availability)
I have reviewed the pre-registration. I do not have matlab so I am not equipped to review the code.

Version 1:

Reviewer comments:

Reviewer #1

(Remarks to the Author)
I already thought the ms. was strong, but the Authors' revisions have strengthened it further, so no notes, well done!

(Remarks on code availability)

Reviewer #2

(Remarks to the Author)
The authors have taken my concerns seriously and have addressed them through significant revisions that have considerably improved the paper. I commend the authors for their hard work and careful efforts. I am satisfied with the changes, and I view this as a valuable contribution to the collective intelligence literature.

-Douglas Guilbeault

(Remarks on code availability)

Reviewer #3

(Remarks to the Author)
The authors have satisfactorily addressed all of my comments.

I also considered the comments shared by Reviewer 2, which were thorough and had not occurred to me. I think Reviewer 2 is correct that we can't be totally sure about mechanisms, and that alternate explanations remain.

This possibility is not a huge concern for me---it is a feature of nearly any causal analysis. Moreover, the evidence provide makes clear that interventions instructing people to use decomposiiton help, and some evidence they're doing that---I think we can punt to future research to verify exactly what mechanism is between the treatment and the outcome. The process and intervention studied here are key to numeric estimation, but have not really been studied in the crowd/group context so it's natural that we will have to settle for incomplete insight.

(Remarks on code availability)

Open Access This Peer Review File is licensed under a Creative Commons Attribution 4.0 International License, which permits use, sharing, adaptation, distribution and reproduction in any medium or format, as long as you give appropriate credit to the original author(s) and the source, provide a link to the Creative Commons license, and indicate if changes were made.
In cases where reviewers are anonymous, credit should be given to 'Anonymous Referee' and the source.
The images or other third party material in this Peer Review File are included in the article's Creative Commons license, unless indicated otherwise in a credit line to the material. If material is not included in the article's Creative Commons license and your intended use is not permitted by statutory regulation or exceeds the permitted use, you will need to obtain permission directly from the copyright holder.
To view a copy of this license, visit https://creativecommons.org/licenses/by/4.0/

Reviewer #1:

In this ms., the Authors study the mechanisms by which groups discussing with each other reach answers that improve on other aggregation procedures such as averaging. In particular, the Authors argue that during discussion, some groups unpack the problem at hand (here, providing numerical estimates) into various dimensions that they estimate independently, before combining them to yield a better estimate. The Authors show that this process is important in three studies, which show (i) that groups that happen to use that decomposition technique more improve more (ii) that teaching groups to do it allows them to improve more and (iii) that teaching individuals to do it doesn't yield the same improvement in performance.

This is a very strong ms., with a solid introduction, good experiments, a sensible discussion, so I have no major comments, and I would recommend accepting with (very) minor revisions.

We thank the reviewer for the positive evaluation of our manuscript and for the encouraging comments. We are glad that the motivation, experimental design, and contribution of the work were clear. We have carefully addressed the points raised below and believe the manuscript has benefited from the reviewer's suggestions.

Minor comments:

1. How would the process described here extend to other problems, besides numerical problems? The Authors mention categorical problems as the other category, but they are not (by far) the only two possibilities. People might have to come up with a plan, with an explanation, with a piece of advice, with the analysis of a situation, etc. The fact that mathematical aggregation techniques don't really apply there doesn't mean that this isn't most of what groups are asked to do. The Authors should, I think, (i) mention this as a limitation of their work (i.e. they focus on a narrow type of problems); (ii) speculate as to how decomposition could apply in other cases.

We agree with the reviewer about this point. In the present work, our experimental setup follows the standard paradigm used to study the wisdom-of-crowds phenomenon, which typically relies on numerical estimation problems with positive quantities. This design allows for clear performance benchmarks and facilitates comparison with prior work on collective estimation.

However, as the reviewer correctly notes, this framework does not capture the full range of problems that groups are asked to solve. Many real-world decisions involve not only categorical judgments, but also explanations, advice, planning, or situation analysis, for which numerical aggregation procedures do not directly apply. We therefore agree that the scope of the present study is limited to a relatively narrow class of tasks.

Following the reviewer's suggestion, we have added a paragraph in the Discussion explicitly acknowledging this limitation and discussing the potential generalizability of collective Fermi-style reasoning to other domains. In particular, we speculate that decomposition-based reasoning may also be useful in problems where complex judgments can be broken down into simpler sub-questions, even when the final outcome is not a numerical estimate. We also highlight this as an important direction for future research.

This paragraph is now on page 30:

However, several limitations remain. As with much of the crowd wisdom literature, it is unclear whether our findings extend to domains where correct answers are categorical rather than continuous (for a notable exception, see Becker et al., (2022)). Moreover, many real-world group decisions involve not only categorical judgments, but also explanations, advice, planning, or the analysis of complex situations, for which numerical aggregation procedures do not directly apply. Our findings therefore speak most directly to a relatively narrow class of estimation problems. However, the mechanism we study (i.e., collective decomposition of a complex problem into simpler components) may generalize beyond numerical estimation. In principle, many complex judgments can be structured as a set of simpler sub-questions whose answers can then be integrated into a final decision. Future work should examine whether collective Fermi-style reasoning can provide similar benefits in domains where the outcome is not a numerical estimate but rather a categorical judgment, explanation, recommendation, or plan of action.

2. Question: do the groups that use decomposition take more time? It should be the case, in that people just telling each other what they think the answer is is very quick, but is it? If yes, is that a drawback (e.g. could the Authors measure improvement as a function of time spent)? If not, why not? (e.g. because the instructions or the experimental set up make it natural for all groups to spend about as much time on the task?)

This is an interesting question. In our experimental setup, participants were instructed to reach consensus within a 5-minute time limit for each question. If consensus was achieved earlier, participants had to remain in the chat until the 5 minutes elapsed before moving on to the next question. As a result, the total time available for discussion was effectively fixed across groups.

Although we did not record the exact time at which consensus was reached within the 5-minute window, we examined conversation length as a proxy for deliberation time. Consistent with the reviewer's intuition, conversations with higher Fermi ratings tended to have more messages (Pearson r = 0.42, p = 6x10-19) and more words (Pearson r = 0.52, p = 2×10^{-30}). However, conversation length itself, measured either as the number of messages exchanged or the total number of words in the conversation, does not predict estimation error (Pearson r = -0.07, p = 0.2, and Pearson r = -0.03, p = 0.6, respectively). This suggests that the improved performance observed in the Fermi condition is not simply a consequence of groups spending more time discussing the problem.

We now report this analysis in the revised manuscript (page 11):

Another potential concern is that Fermi-based discussions may simply take longer than conversations focused on sharing numbers and this alone may drive performance. However, while conversations with higher Fermi ratings indeed tended to be longer (Pearson r = 0.42, p = 6×10^{-19}), conversation length itself did not predict estimation error (Pearson r = -0.07, p = 0.2), suggesting that the observed performance gains are not simply driven by groups spending more time discussing the problem.

3. I don't really get that bit "Moreover, the Similarity index was negatively associated with estimation error (Fig. 3B; Pearson r = –0.15, p = 0.007), and this effect was significantly mediated by "Fermi" ratings (Fig. 3C; total effect = –0.11 ± 0.03, p = 0.006; direct effect = –0.08 ± 0.04, p = 0.02; indirect effect = –0.03 ± 0.01, p = 0.009; see Methods for details)."

The reviewer is right that this sentence was difficult to parse in the original version. To improve clarity, we have rewritten this passage in the manuscript. The revised text now reads (page 14):

Moreover, the Similarity index was negatively associated with estimation error (Fig. 3B; Pearson r = –0.15, p = 0.007), indicating that conversations more semantically related to the estimation problem tended to produce more accurate collective estimates. A mediation analysis further showed that this relationship was partially explained by Fermi ratings (Fig. 3C; total effect = –0.11 ± 0.03, p = 0.006; direct effect = –0.08 ± 0.04, p = 0.02; indirect effect = –0.03 ± 0.01, p = 0.009).

We hope this revised wording makes the interpretation of this analysis clearer.

4. I find it weird that the Authors talk about 'discussed questions' in the individual condition of Study 3, since these people didn't discuss with anyone, right? Do the Authors mean something like 'reflected on'? Should they use 'deliberated' (instead of 'discussed') throughout, as it can apply both to group and to individual contexts?

The reviewer is correct that the term "discussed" is misleading in the individual condition of Study 3, since participants in that condition did not interact with others. Following the reviewer's suggestion, we have replaced "discussed" with "deliberated" throughout the manuscript where appropriate, as this term applies more naturally to both individual and group contexts.

5. Have the Authors analyzed how exactly groups who use decomposition improve? Does it tend to improve on the best estimate already present in the group, or does it mostly bring the outliers towards the average? (and which of these two does it do more than just sharing one's opinion?)

This is an interesting question. Because the design of the experiment yields four individual estimates and one collective estimate per group, we cannot directly assess whether decomposition primarily moves outliers toward the group average. However, following the reviewer's suggestion, we examined whether the two procedures differ in their ability to produce collective estimates that outperform the best individual estimate in the group.

For each conversation, we ranked the four individual estimates according to their individual error (from most to least accurate) and computed the probability that the collective estimate was more accurate than each of the individual estimates. As expected, the collective estimate always improves upon the least accurate individual estimate regardless of condition. However, differences between conditions emerge for more accurate individuals. To quantify this pattern, we computed for each conversation the fraction of initial individual estimates outperformed by the collective answer and compared this measure across conditions. A Wilcoxon rank sum test indicated that collective estimates under the Fermi condition outperformed a larger fraction of initial estimates than those produced in the Numbers condition (Fermi: 0.73±0.03, Numbers: 0.63±0.03, z=2.33, p=0.02, Cohen's d = 0.44)

Following the reviewer's suggestion, we now report this analysis in the revised manuscript (page 17 and Fig. S4), which provides additional insight into how decomposition-based reasoning improves collective estimates:

We then conducted an additional exploratory analysis examining whether the collective estimate outperformed the individual estimates within each group. For each conversation, we ranked the four individual estimates according to their individual error (from most to least accurate) and computed the probability that the collective estimate was more accurate than each individual estimate (Fig. S4). Collective estimates always improved upon the least accurate individual estimate regardless of condition. However, differences between strategies emerged for more accurate individuals.

To quantify this pattern, we computed the fraction of initial individual estimates outperformed by the collective answer and compared this measure across conditions. A Wilcoxon rank-sum test indicated that collective estimates under the Fermi condition outperformed a larger fraction of initial estimates than those produced in the Numbers condition (Fermi: 0.73±0.03, Numbers: 0.63±0.03, $z=2.33$, $p=0.02$, Cohen's $d = 0.44$). This suggests that the advantage of the Fermi condition lies not only in correcting poor initial estimates, but also in more often surpassing the most accurate individual judgments in the group.

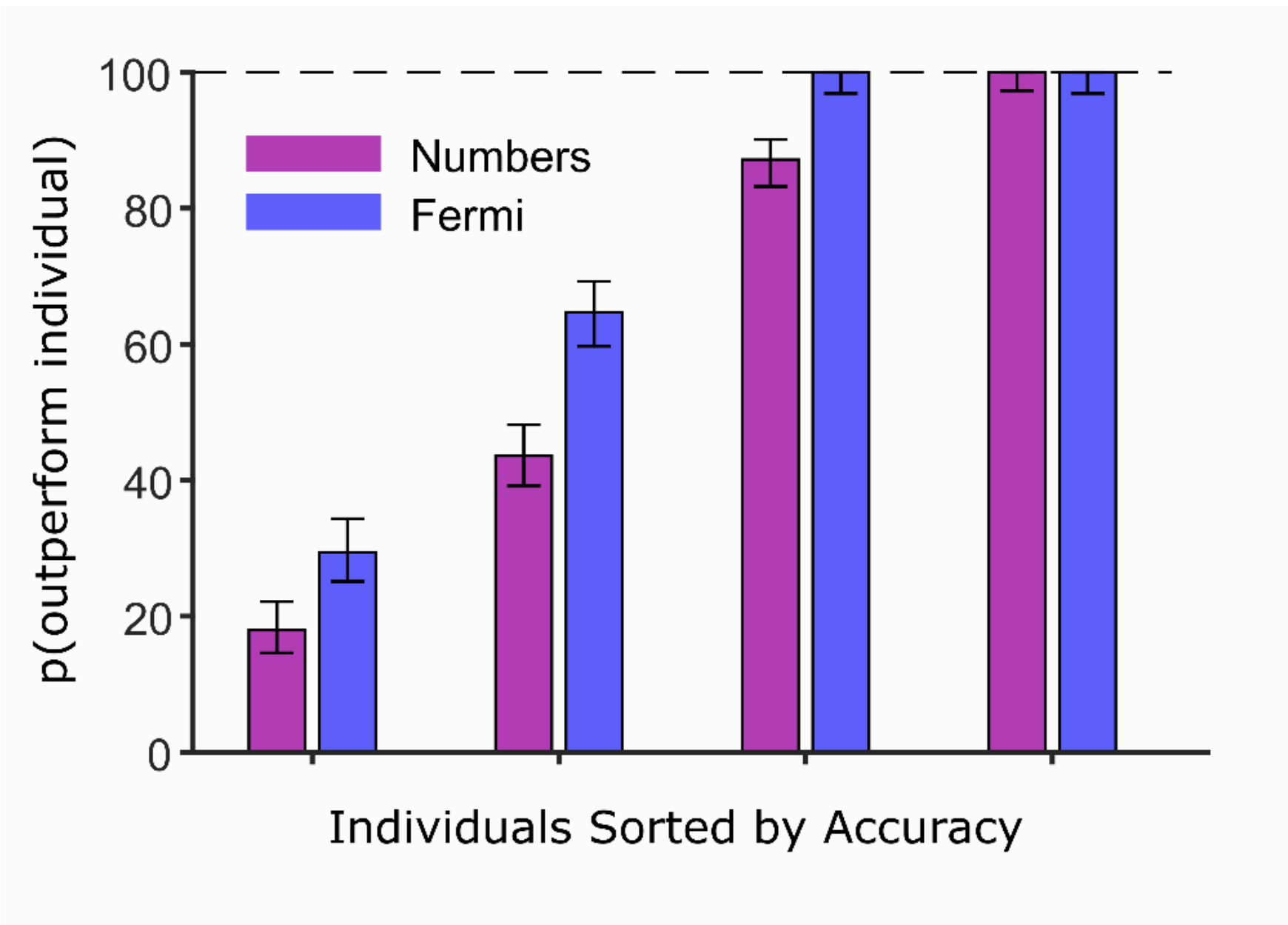

Fig. S4. Probability that the collective estimate outperforms individual estimates. In Study 2, individuals within each group were ranked according to their individual estimation error (from most to least accurate). The figure shows the probability that the collective estimate outperforms the estimate of each individual in the group under the two experimental conditions ("Numbers" and "Fermi"). Error bars represent ±1 s.e.p.

6. Finally, could the Authors say a bit more about why they think decomposition works better in groups? Is it because different people are better at estimating different elements? Or because groups just do more decomposition than individuals, even when instructed?

This is an important question. In the revised manuscript we now expand the Discussion to address this point more explicitly. As shown in Study 3, the Fermi method improves performance both for individuals and for groups when participants are instructed to apply it, but the improvement is substantially larger in groups. This raises the question of why the same reasoning strategy yields stronger benefits in collective settings.

Drawing on prior work on collective cognition, we suggest that this difference may arise because group deliberation enables supra-personal metacognitive processes, such as monitoring and correcting errors in others' reasoning (e.g., Bahrami et al., 2010; Frith, 2012; Shea et al., 2014). In the context of Fermi-style estimation, this is particularly plausible since decomposing a problem generates multiple intermediate assumptions and sub-estimates that can potentially be evaluated and revised during discussion.

To examine this possibility, we analyzed linguistic markers associated with error-correction dynamics in Study 3, where participants explained their reasoning. Specifically, we developed a linguistic indicator capturing error-correction dynamics, based on expressions typically associated with identifying, evaluating, or correcting intermediate estimates. These include expressions that signal explicit calibration of quantities, such as phrases indicating magnitude adjustment (e.g., "too little", "too much", "too many") or evaluative corrections (e.g., "better", "more reasonable").

Using this measure, we find that these linguistic markers occur substantially more frequently in group deliberation than in individual reasoning (unpaired t-test: $t(360)=-6.7$, $p=8x10-11$). Moreover, higher levels of these error-correction dynamics are associated with lower estimation error in the subsequent individual phase (Pearson $r = -0.18$, $p = 0.0001$).

Importantly, these markers do not differ between the Fermi and Numbers conditions in Study 2 (unpaired t-test: $t(177)=0.9$, $p=0.4$), suggesting that they help explain why collective deliberation outperforms individual reasoning, but not why the Fermi method itself improves performance relative to simple number sharing.

Following the reviewer's suggestion, we now report these analyses in a new section of the Results and added a new supplementary figure (Fig S5), which further strengthens the mechanistic interpretation of our findings (page 26):

Why does the Fermi method benefit groups more than individuals?

Study 3 allowed us to examine whether the benefits of the Fermi method differ depending on whether the strategy is applied individually or collectively. In this experiment, all participants were instructed to apply the Fermi method, but some deliberated individually while others deliberated in groups. In both cases, participants improved their estimates relative to their initial responses but the magnitude of this improvement was substantially larger in the collective condition (Figs. 5C and 5D).

One possible explanation to this effect is that collective deliberation enabled metacognitive processes that are difficult to reproduce in isolation. Prior work in collective cognition suggests that interactive reasoning can benefit from supra-personal monitoring processes, such as detecting and correcting errors in others' reasoning (e.g., Bahrami et al., 2010; Frith, 2012; Shea et al., 2014). In the context of Fermi estimation, this mechanism is plausible because decomposing a problem generates multiple intermediate assumptions that can be re-evaluated during deliberation.

To test this possibility, we conducted an exploratory analysis of the language produced by participants in Study 3. We constructed a linguistic indicator capturing error-correction dynamics, based on expressions typically associated with evaluating or adjusting magnitudes (e.g., phrases like "too little" or "too much", or evaluative corrections such as "better"). These expressions occurred substantially more frequently in collective deliberation than in individual reasoning (Fig. S5, unpaired t-test: t(360)=-6.7, p=8x10-11, Cohen's d = 0.91), and higher levels of such language were associated with lower estimation error in the subsequent individual stage (Pearson r = −0.18, p = 0.0001). Importantly, these markers did not differ between the Fermi and Numbers conditions in Study 2 (Fig. S5, unpaired t-test: unpaired t-test: t(177)=0.9, p=0.4, Cohen's d = 0.14), and did not predict collective error in Study 1 (Pearson r = 0.31, p = 0.58). This pattern suggests that error-correction dynamics help explain why collective deliberation amplifies the benefits of the Fermi method, rather than why the Fermi method itself improves performance relative to number sharing.

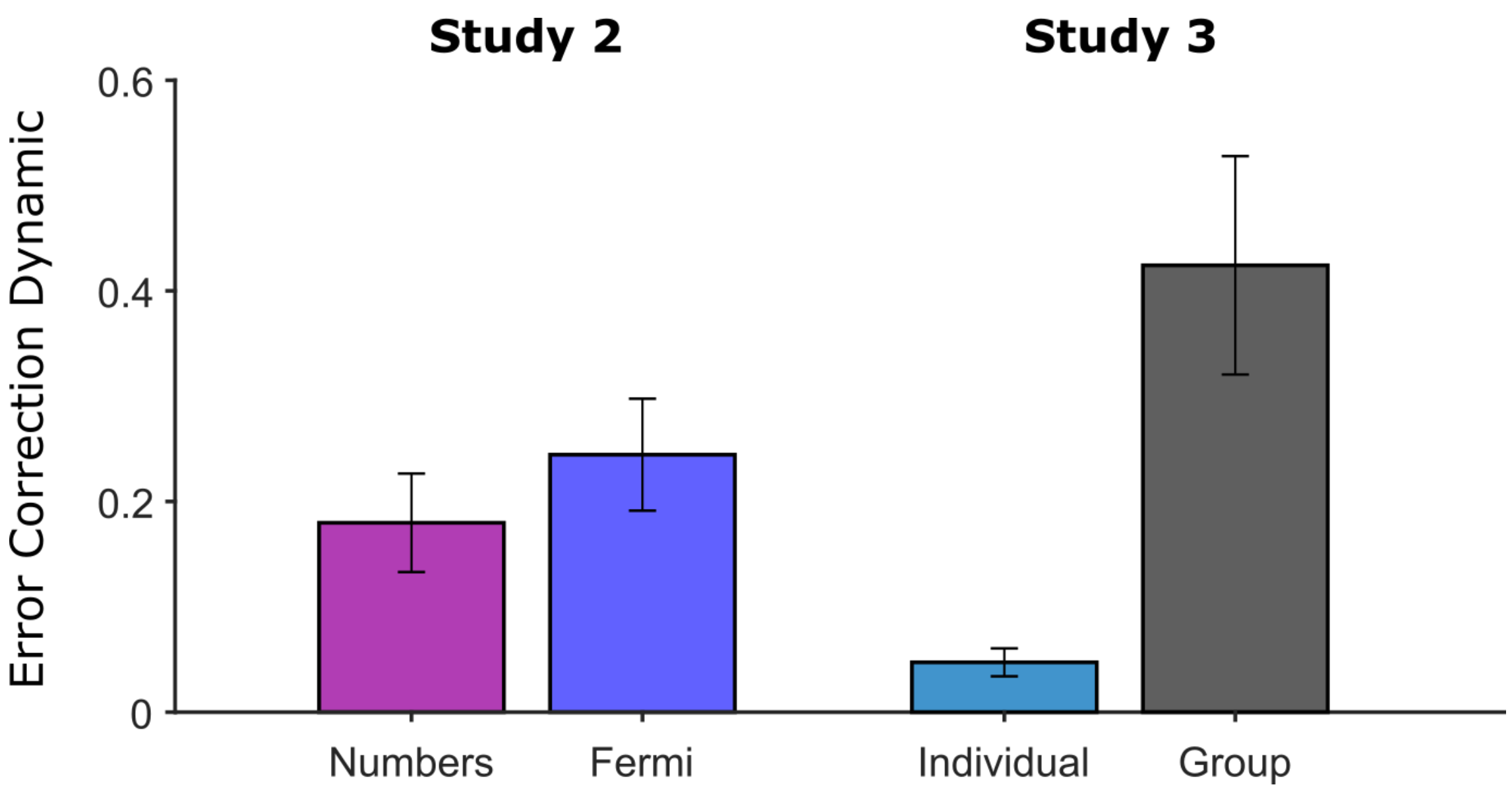

Fig. S5. Error correction dynamics in Studies 2 and 3. In Study 2, the presence of linguistic markers reflecting error correction dynamics (e.g., "too little", "too much", "better") was not statistically different between conditions involving different instructions. In Study 3, the same linguistic maker led to a pronounced and significant different between the individual and collective condition. Bars show the average value of the metric for each study and condition ±1 s.e.m.

Reviewer #2:

Review of “Collective problem decomposition drives the wisdom of deliberative crowds”

There is much to like in this paper. The core idea is interesting. However, I found the NLP related methods to be considerably limited and lacking, and ultimately, I did not find that a mechanistic analysis of the main effect was clearly articulated or supported. This stems, in my view, from a lack of a clear definition and operationalization of “problem decomposition”. That said, I do think it may be possible to address these concerns in a revision. At this stage, I’ll focus my comments on my major concerns.

We thank the reviewer for the thoughtful and constructive evaluation of our manuscript. We appreciate the positive assessment of the core idea of the paper, as well as the issues raised regarding the clarity of the mechanism and the operationalization of problem decomposition. We took these concerns very seriously and substantially revised the manuscript to address them.

First, we improved the NLP analyses by incorporating new linguistic measures that more directly capture problem decomposition, addressing the reviewer’s concern that our original semantic similarity metric was too coarse to capture the relevant reasoning processes. These new measures allow us to identify linguistic markers associated with the decomposition of estimation problems into subcomponents.

Second, we now provide a clearer mechanistic account of why problem decomposition benefits collective deliberation more than individual reasoning. In the revised manuscript, we present evidence that the advantage of groups emerges from error-correction dynamics that unfold during discussion. These dynamics can be identified through specific linguistic markers that appear systematically in group conversations but are largely absent in individual reasoning.

Third, following the reviewer’s suggestion, we now provide a clearer conceptual definition and operationalization of the Fermi method. In the revised manuscript, we explicitly define it as the combination of two processes: problem decomposition and rough approximations (i.e., *guesstimation*) of intermediate variables. This clarification allows us to more precisely characterize the reasoning strategy implemented by participants.

Fourth, we evaluated the alternative mechanisms proposed by the reviewer, including explanations based on information density and linguistic convergence during discussion. The results of these analyses suggest that these factors cannot account for the observed effects.

Finally, we carefully considered the reviewer’s concern that the human annotation procedure might have been influenced by the instructions given to raters. To address this possibility, we implemented an additional validation procedure in which the formal definition of the Fermi method (introduced in the revised manuscript) was provided to five independent large language models (LLMs). These models produced Fermi ratings that closely matched those obtained from human raters, and all main results of the paper replicate when using the LLM-based ratings instead of the human annotations.

We believe that these revisions substantially strengthened both the conceptual clarity and the empirical evidence presented in the paper, and wholeheartedly thank the reviewer for the constructive feedback.

Below, we provide point-by-point replies to each of the points raised by the reviewer:

1. Unclear/Absence of Mechanism.

1.1. The authors argue that problem decomposition mostly only benefits collective deliberation but not independent, individual reasoning, though no mechanistic explanation of why problem decomposition only benefits collectives is provided, despite it being the main finding. If problem decomposition is a superior reasoning strategy, it should benefit individuals as well (which they only find weak support for in study 2). This leads me to wonder whether PD is really driving the effect or some other known factor in collective intelligence that PD indirectly activates but is not directly responsible for.

The reviewer raises an important question regarding the mechanism underlying the stronger benefits of PD in collective settings. Before addressing this point, it is helpful to clarify what each experiment in the paper was designed to test.

Study 2 directly examines whether Fermi estimation improves collective accuracy relative to number sharing. In this experiment, groups were randomly assigned to receive instructions promoting either the Fermi method or a procedure based on exchanging numerical estimates. We observe that groups instructed to use the Fermi method produce significantly more accurate collective estimates than those instructed to share numbers.

Study 3 addresses a different question: whether the Fermi strategy yields different gains when applied individually versus collectively. In this experiment, all participants were instructed to apply the Fermi method, but some deliberated individually while others deliberated in groups. In both conditions, participants improved their estimates relative to their initial answers, showing that the Fermi method benefits reasoning in general. However, the magnitude of the improvement was substantially larger in the collective condition ($z = 6.3$, $p = 3\times10^{-10}$; Cohen's $d = 0.58$).

The primary mechanism examined in the revised manuscript concerns why the Fermi method improves collective estimates relative to number sharing, which we address through new linguistic analyses described in our responses to points 2.1–2.3 below. However, the reviewer raises an additional and important question: why does the same reasoning strategy produce larger gains in groups than in individuals? This is a mechanistic question about collective-versus-individual gains for a fixed strategy, and not about why one strategy is better than other.

To investigate this question, we drew on a large body of work in collective cognition showing that group deliberation can benefit from supra-personal metacognitive processes, such as monitoring and correcting errors in others' reasoning (e.g., Bahrami et al., 2010; Frith, 2012; Shea et al., 2014). In the context of Fermi-style reasoning, this mechanism is particularly plausible because decomposing a problem generates multiple intermediate assumptions and sub-estimates that can potentially be evaluated and corrected during discussion.

To test this mechanism, we analyzed the language produced by participants in Study 3, where both individuals and groups were instructed to explain their reasoning. Specifically, we developed a linguistic indicator capturing error-correction dynamics, based on expressions typically associated with identifying, evaluating, or correcting intermediate estimates. These include expressions that signal explicit calibration of quantities, such as phrases indicating magnitude adjustment (e.g., "too little", "too much", "too many") or evaluative corrections (e.g., "better").

Using this measure, we find that these linguistic markers occur substantially more frequently in group deliberation than in individual reasoning (unpaired t-test: $t(360)=-6.7$, $p=8\times10^{-11}$). Moreover, higher levels of these error-correction dynamics are associated with lower estimation error in the subsequent individual phase (Pearson $r = -0.18$, $p = 0.0001$).

At the same time, we took seriously the reviewer's concern that this mechanism might itself be the primary driver of the observed performance gains, rather than the Fermi method per se. Importantly, the design of Study 3 cannot directly address this question, because all participants were explicitly instructed to apply the Fermi method and the only manipulation concerns whether it is applied individually or collectively.

To evaluate this possibility, we therefore turned to Study 2, where groups were randomly assigned either to apply the Fermi method or to exchange numerical estimates. If the performance advantage of the Fermi condition were driven primarily by increased error-correction dynamics, one would expect these dynamics to occur more frequently in the Fermi condition. However, we do not observe such differences between conditions (unpaired t-test: $t(177)=0.9$, $p=0.4$) nor a correlation between these linguistic markers and collective error (Pearson $r = -0.05$, $p = 0.58$). A similar pattern is observed when, in Study 1, they weren't asked to apply any specific method (Pearson $r = 0.31$, $p = 0.58$). This suggests that error-correction dynamics help explain why collective reasoning outperforms individual reasoning, but do not account for the additional advantage of applying the Fermi method itself.

To examine the mechanism underlying this latter effect, we developed new NLP measures that capture linguistic markers specifically associated with the Fermi method, based on its formal definition introduced in the revised manuscript. These analyses are described in detail in our response to points 2.1 to 2.3 below.

Taken together, these new analyses help clarify the mechanism underlying our findings: The Fermi method improves reasoning in general, but collective deliberation amplifies its benefits because interactive settings enable error detection and correction processes that are difficult to reproduce in isolation.

We thank again the reviewer for raising this very important point. Based on these comments, we have now included a new section in page 26 and Fig. S5:

Why does the Fermi method benefit groups more than individuals?

Study 3 allowed us to examine whether the benefits of the Fermi method differ depending on whether the strategy is applied individually or collectively. In this experiment, all participants were instructed to apply the Fermi method, but some deliberated individually while others deliberated in groups. In both cases, participants improved their estimates relative to their initial responses but the magnitude of this improvement was substantially larger in the collective condition (Figs. 5C and 5D).

One possible explanation to this effect is that collective deliberation enabled metacognitive processes that are difficult to reproduce in isolation. Prior work in collective cognition suggests that interactive reasoning can benefit from supra-personal monitoring processes, such as detecting and correcting errors in others' reasoning (e.g., Bahrami et al., 2010; Frith, 2012; Shea et al., 2014). In the context of Fermi estimation, this mechanism is plausible because decomposing a problem generates multiple intermediate assumptions that can be re-evaluated during deliberation.

To test this possibility, we conducted an exploratory analysis of the language produced by participants in Study 3. We constructed a linguistic indicator capturing error-correction dynamics, based on expressions typically associated with evaluating or adjusting magnitudes (e.g., phrases like "too little" or "too much", or evaluative corrections such as "better"). These expressions occurred substantially more frequently in collective deliberation than in individual reasoning (Fig. S5, unpaired t-test: t(360)=-6.7, p=8x10-11, Cohen's d = 0.91), and higher levels of such language were associated with lower estimation error in the subsequent individual stage (Pearson r = −0.18, p = 0.0001). Importantly, these markers did not differ between the Fermi and Numbers conditions in Study 2 (Fig. S5, unpaired t-test: unpaired t-test: t(177)=0.9, p=0.4, Cohen's d = 0.14), and did not predict collective error in Study 1 (Pearson r = 0.31, p = 0.58). This pattern suggests that error-correction dynamics help explain why collective deliberation amplifies the benefits of the Fermi method, rather than why the Fermi method itself improves performance relative to number sharing.

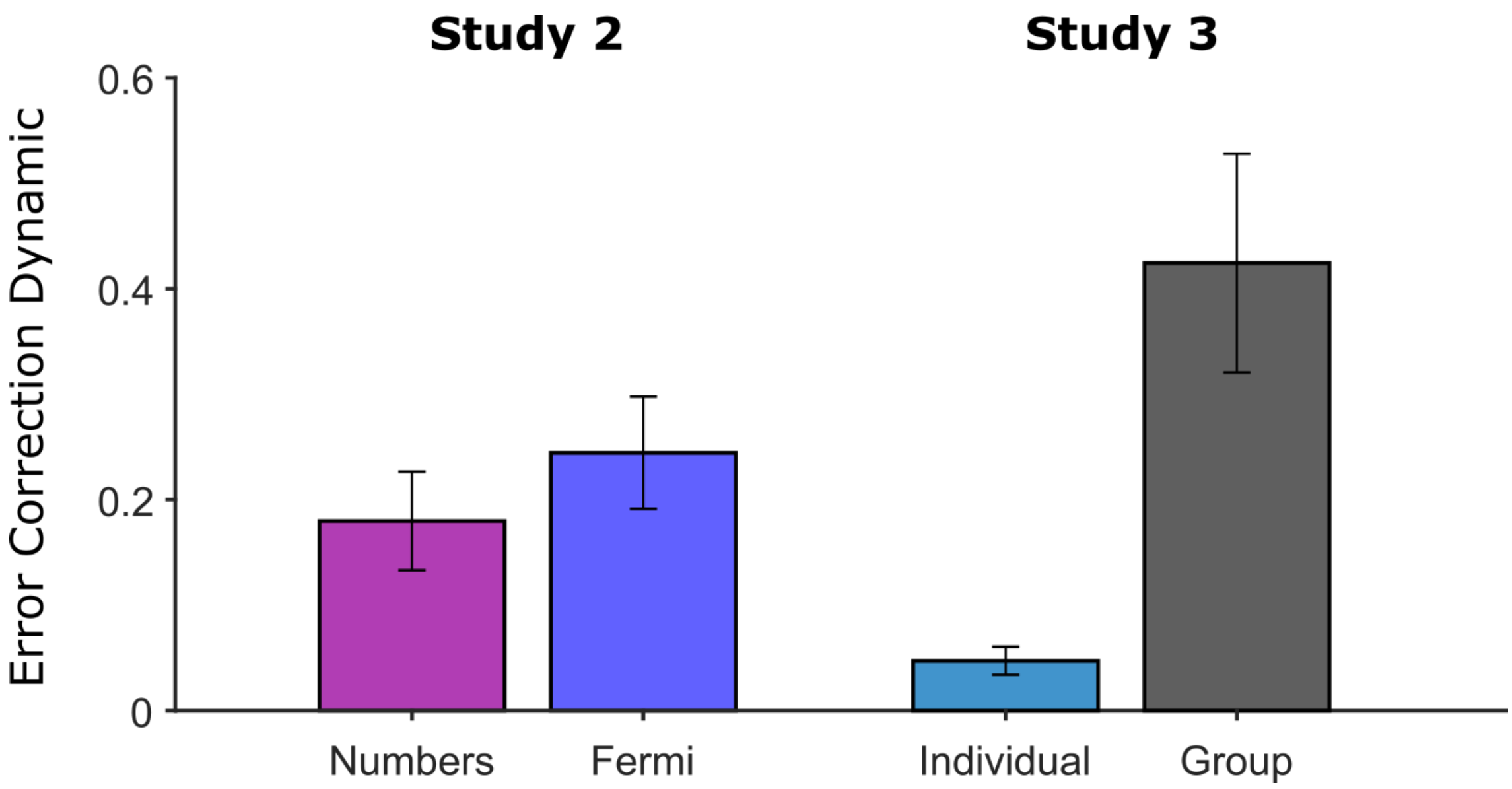

Fig. S5. Error correction dynamics in Studies 2 and 3. In Study 2, the presence of linguistic markers reflecting error correction dynamics (e.g., "too little", "too much", "better") was not statistically different between conditions involving different instructions. In Study 3, the same linguistic maker led to a pronounced and significant different between the individual and collective condition. Bars show the average value of the metric for each study and condition ±1 s.e.m.

1.2. For example, consider the robust finding that the wisdom of the crowds and collective estimation benefits from cognitive diversity (e.g., the diversity-prediction theorem and Page's work). It could simply be the priming people to only share numbers restricts available diversity only to numbers, whereas priming them to PD introduces more conceptual diversity by getting people to share the differing perspectives (reasons) underlying their numbers. People with the same estimates, after all, can arrive at these estimates for different reasons. Encouraging the exchange of reasons (or, in other words, allowing conversation to not be arbitrarily restricted to number exchanges) creates more surface area for conceptual diversity to link into the conversation beyond just the numbers. Measuring the raw number of words in the chat is not a good measure for this because it's incredibly coarse. What matters are more substantive differences in the breadth of reasoning engaged. This is where a measure of information density at the language level is likely to be much more appropriate (see reference from Aceves & Evans 2024 below, which provides one among several possible measures of information density). But note that if this argument is supported, it's not clear that the effect is driven by PD specifically, but rather than the PD condition simply allows more information diversity on top of number exchange. This concern connects with my doubts that the linguistic measures of PD proposed are actually measuring what the authors think they are measuring.

• Aceves, P., Evans, J.A. Human languages with greater information density have higher communication speed but lower conversation breadth. Nat Hum Behav 8, 644–656 (2024). https://doi.org/10.1038/s41562-024-01815-w

We thank the reviewer for raising this interesting alternative explanation and for pointing us to the work of Aceves & Evans (2024). We agree that an increase in conceptual or informational diversity during discussion could, in principle, contribute to improved collective estimates. Indeed, if the Fermi instruction simply expanded the diversity of information exchanged (by encouraging participants to articulate the reasons underlying their estimates) this could have potentially accounted for the observed performance gains without requiring problem decomposition to be the key mechanism. We therefore agree with the reviewer that evaluating this possibility is important.

To address this concern, we implemented the information density measure proposed by Aceves & Evans (2024) and tested whether it could account for the observed effects. While information density is only one possible proxy for conversational diversity, it provides a principled way to test the reviewer's hypothesis within our data.

Across studies, we find that the effect of Fermi decomposition on performance remains statistically significant after controlling for information density. In fact, information density itself does not significantly predict group error in Study 1 (Pearson correlation: $r = -0.06$, $p = 0.32$) or Study 2 (Pearson correlation: $r = -0.08$, $p = 0.38$). In Study 3, all participants were explicitly instructed to apply the Fermi method. Because of this design, the study does not allow us to directly evaluate whether differences in information density can explain the performance advantage associated with problem decomposition.

Finally, it is also unlikely that the observed effects are driven solely by differences in instructions restricting conversational diversity. In Study 1, participants received no instructions regarding how to deliberate, and groups naturally varied in the extent to which they employed Fermi-style reasoning. In this setting, greater use of the Fermi method (originally assessed through human annotations and now replicated using both LLM-based ratings) consistently predicts lower estimation

error (Pearson correlation: r = –0.20, p = 2x10^{-4}). This pattern suggests that the observed relationship between Fermi reasoning and improved performance cannot be explained simply by instruction-induced constraints on information exchange.

We thank the reviewer for suggesting this important alternative mechanism. In the revised manuscript (page 18), we now explicitly discuss this hypothesis and report the corresponding analyses:

Discarding artefactual effects of instructions

To examine whether the observed advantage of the Fermi condition could be explained by mechanisms other than problem decomposition itself, we conducted additional exploratory analyses evaluating two alternative hypotheses. One possibility is that the Fermi instructions simply increased the diversity of information exchanged during discussion, for example by encouraging participants to articulate the reasons underlying their estimates. If this were the case, the observed gains in the Fermi condition might reflect greater informational diversity rather than problem decomposition per se.

To test this possibility, we implemented an information density metric previously proposed as a proxy for conversational informational diversity (Aceves & Evans, 2024). However, we observed that information density did not significantly predict collective error (Pearson correlation: r = –0.08, p = 0.38). These results suggest that the improved performance observed in the Fermi condition cannot be explained simply by increased informational diversity during discussion.

1.3. Another possible explanation is that the effects of PD depend on deliberation not because of PD specifically, but rather because priming for the exchange of reasons increases coordination. It's not simply that people share reasons (on top of numbers), but that they are tasked with coordinating their reasons and their numbers. It could be that inducing coordination of reasons alone improves performance, regardless of whether these reasons engage in a specific strategy called PD. This is important because the linguistic measures of the authors do not, in my opinion, clearly establish that their treatment condition induced PD specifically, due in part to the vagueness of their measure of PD (described in more detail below). One possible way to measure this would be to test whether, over time, participants in the PD condition exhibit greater increases in the similarity of their language/framings, in comparison to just those in the numbers condition. It could be that PD simply increases available information and induces more coordination/alignment over time, both of which are known vectors for improving collective intelligence. While this would still be interesting as a finding for establishing the value of deliberation, it's less novel and wouldn't necessarily support PD specifically as the mechanism (since, as factors, they are not explanations of why PD works, but rather mechanisms that are more general than PD and do not depend on PD specifically). More needs to be done to confirm that PD specifically is at a play and to explain why PD's benefits yield mainly in collective rather than individual contexts.

The reviewer raised an interesting alternative explanation to our findings. We agree that increased coordination during discussion could, in principle, contribute to improved collective performance. In particular, if the Fermi condition simply encouraged participants to align their reasoning and coordinate their assumptions over time, this could potentially account for the observed performance gains without requiring Fermi estimation to be the key mechanism.

To evaluate this possibility, we followed the reviewer's suggestion and tested whether conversations in the Fermi condition exhibit stronger semantic convergence over time than those in the Numbers condition. Using the same FastText-based semantic similarity pipeline employed in our Similarity analyses, we computed message-to-message semantic similarity throughout each conversation and estimated a temporal slope for each discussion.

Contrary to the coordination hypothesis, we do not observe evidence of increasing semantic convergence over time in the Fermi condition. The estimated slopes are close to zero in both conditions (mean slope = −0.02 in the Numbers condition and −0.005 in the Fermi condition), indicating that conversations do not become more semantically aligned as they progress. Indeed, the distribution of best-fitting slopes in the Fermi condition are not significantly different from zero (t-test: t(89) = -1.5, p = 0.1) and the best-fitting slopes do not predict collective error (Pearson r = 0.02, p = 0.82).. These results remain qualitatively unchanged when applying a sliding-window approach to compute local convergence patterns (distribution of slopes, mean ± S.D. = −0.0007 ± 0.009, t-test: t(89) = -0.8, p = 0.4; correlation with collective error: Pearson r = 0.07, p = 0.45).

Taken together, these analyses do not support the hypothesis that the Fermi condition improves performance by increasing semantic alignment or coordination during discussion. We thank the reviewer for suggesting this robustness test. The corresponding analyses and discussion have now been added to the revised manuscript (page 18):

A second possibility is that the Fermi instructions improved performance by increasing coordination or semantic alignment among participants during deliberation. Following this hypothesis, we tested whether conversations in the Fermi condition exhibit stronger semantic convergence over time than those in the Numbers condition. Using the same FastText-based semantic similarity pipeline we previously presented (Fig. 3), we estimated the slope of message-to-message semantic similarity within each conversation. Contrary to the coordination hypothesis, we do not observe increasing semantic convergence over time in the Fermi condition (distribution of slopes, mean ± S.D. = −0.005 ± 0.03, t-test: t(89) = -1.5, p = 0.1) and the best-fitting slopes do not predict collective error (Pearson r = 0.02, p = 0.82). These results remain qualitatively unchanged when applying a 5-message sliding-window approach to compute local convergence patterns (distribution of slopes, mean ± S.D. = −0.0007 ± 0.009, t-test: t(89) = -0.8, p = 0.4; correlation with collective error: Pearson r = 0.07, p = 0.45). Overall, these findings do not support the hypothesis that the performance gains observed in the Fermi condition are driven by semantic alignment between individuals during deliberation.

2. Unclear definition/operationalization/measurement of problem decomposition (PD).

2.1. The authors' definition of collective fermi estimation is vague and includes several different processes, explicitly referencing both 'rough approximations' and 'problem decomposition' (which are quite distinct and can exist without each other), while also implicitly describing this process as increasing 'reasoning' (which is much more general than PD specifically and can involve all kinds of processes, from argumentation to counterfactual analysis). This vagueness makes it really unclear how it can be effectively measured. This is more concerning when considering that the main measures the authors provide are very basic and not directly related to any of the processes above, but instead are a loose proxy that is supported by correlations of varying strength. Specifically, they attempt to proxy PD by measuring the semantic relatedness of language in the conversation to the initial estimation task phase. Not only is this measure unrelated to reasoning directly (all kinds of language can satisfy this measure but be unrelated to reasoning, let alone PD specifically), but this measure is also likely confounded with the task design, because those in the number sharing condition are asked to focus on sharing numbers, which will reduce the overall language used and impact this similarity metric but for trivial reasons.

We thank the reviewer for this important comment. We agree that the original manuscript did not provide a sufficiently precise operationalization of problem decomposition within the Fermi method. In response to this point, we substantially revised the manuscript to clarify the conceptual definition of the Fermi method and to develop more targeted linguistic measures that directly capture its underlying reasoning processes. As we explain below, these changes led to a clearer conceptual framework and new analyses that more directly test the mechanisms underlying our findings.

First, following the reviewer's suggestion, we now provide a formal definition of the Fermi method in the Introduction. To do this, we first searched for previous works providing definitions of the Fermi method (Table S1):

Albarracín, L., & Gorgorió, N. (2014).	(The Fermi method is) "…based on designing a problem-solving strategy adapted to the problem which consists of breaking it into subproblems that may be solved separately by means of their respective estimations."
Albarracín, L., & Gorgorió, N. (2019).	"The procedure proposed by Fermi to his students was to decompose the original problem into simpler sub-problems to reach a solution of the original question by means of reasonable estimates or educated guesses"
Abay, S., & Filiz, S. B. (2020).	"Fermi problems are open-ended, non-routine problems that require students to make systematic guesses by making assumptions before starting on a solution using simple calculations."
Gomilsek et al. (2024)	"Fermian strategies prescribe decomposing an estimation problem into subtasks, solving the subtasks separately, and ultimately integrating those solutions into a final estimate."
Albarracín, L., & Ärlebäck, J. B. (2025)	"The Fermi estimation method is the classic approach to solving Fermi problems, in which assumptions are made to decompose the original problem into simpler subproblems that are solved separately using reasoned estimates based on knowledge of the real world."

We then noted that these defnitions combine two key steps: (i) problem decomposition, in which the target quantity is broken into simpler components, and (ii) rough approximation ("guesstimation"), in which approximate values are proposed for those components and subsequently recombined to produce a final estimate. Based on this observation, we provide the following definition (page 5):

"*The Fermi Method consists of breaking the problem into simpler components, making rough estimations for each component, and calculating the final answer based on those estimations*."

In other words, we agree with the reviewer that Fermi estimation involves two distinct but complementary processes, and the revised manuscript now makes this distinction explicit. In the revised manuscript we now explicitly anchor our definition in this prior work and clarify how these two elements jointly characterize the reasoning strategy implemented in our experiments.

Second, regarding the Similarity measure used in the original manuscript, we agree with the reviewer that this metric is relatively coarse. Our intention in introducing this analysis was not to provide a mechanistic test of Fermi reasoning, but rather to demonstrate that a simple and low-cost NLP proxy could identify conversational patterns associated with lower collective error. This motivation is relevant because extracting the wisdom of crowds from deliberative settings has numerous potential applications (e.g., prediction markets, financial forecasting, health decision-making, etc.) and scalable, low-cost methods for identifying high-performing conversations could therefore be practically valuable. We now explicitly state so in the manuscript (page 13):

Predicting collective accuracy through automated language analysis

Because identifying groups that implemented the Collective Fermi Estimation strategy relies on human ratings, the process remains costly, time-consuming, and difficult to scale. To address this, we developed a computational method to automatically identify high-performing conversations. The objective of this analysis is not to provide a mechanistic test of Fermi reasoning, but rather to examine if a simple and low-cost linguistic proxy could identify conversational patterns associated with higher Fermi ratings and lower collective error. This motivation is relevant because extracting the wisdom of crowds from deliberative settings has numerous potential applications (e.g., prediction markets, financial forecasting, health decision-making, etc.) and scalable methods for identifying high-performing conversations could therefore be practically valuable.

However, we agree with the reviewer that this measure alone cannot establish that groups perform better specifically because they engage in Fermi-style reasoning. To address this concern, we developed new linguistic indicators that directly operationalize the two components of the Fermi method. Specifically, we constructed separate measures capturing (i) problem decomposition, reflected in expressions that break the target quantity into multiplicative or component-based structures (e.g., "per", "each", "every", "times"), and (ii) guesstimation, reflected in linguistic markers indicating approximate numerical reasoning under uncertainty. These include expressions proposing numerical ranges (e.g., "between N and M") and approximate quantities (e.g., "approximately N", "around N", "about N). Importantly, these markers were derived directly from the formal definition of the Fermi method introduced above.

Using these indicators, we find that both problem decomposition and guesstimation strongly predict Fermi ratings across studies. In Study 1, both types of linguistic markers significantly predict human-annotated Fermi ratings (β_{PD} = 0.54, p = 5×10^{-19}; β_{GE} = 0.43, p = 0.01). In Study 2, the effect of the experimental manipulation (Numbers vs. Fermi instructions) on Fermi ratings is significantly mediated by the increased presence of these markers in the conversations (total effect: 2.56±0.36, p=0.0006, direct effect: 1.86±0.37, p=0.0005, indirect effect of PD: 0.46±0.12, p=0.0004; indirect effect of GE: 0.25±0.11, p=0.01). These patterns hold for both human-annotated ratings and AI-based ratings (see point 2.2 below).

Taken together, these analyses suggest that conversations receiving higher Fermi ratings systematically exhibit the linguistic signatures predicted by the formal definition of the method. We thank the reviewer for raising this important point. In response, we have added a new section (pages 19-22) and figure (Figure 5) in the revised manuscript examining the linguistic mechanisms underlying Fermi ratings, which in turn predict lower collective estimation error across all three experiments.

The new section now reads:

Linguistic signatures of Fermi ratings

To examine whether conversations identified as "Fermi" indeed reflect the reasoning processes described in its definition, we developed linguistic indicators capturing the two components of the method: problem decomposition and guesstimation. Problem decomposition was measured through expressions that break the target quantity into component structures (e.g., multiplicative or per-unit reasoning such as "per", "each", "every", or "times"). Guesstimation was measured through linguistic markers reflecting approximate numerical reasoning under uncertainty, including expressions proposing numerical ranges (e.g., "between N and M", where N and M are numbers) and approximate quantities (e.g., "approximately N", "around N", or "about N", where N is a number).

Across studies, these linguistic indicators strongly predict the extent to which conversations exhibit Fermi-style reasoning. In Study 1, both decomposition and guesstimation markers significantly predict human-annotated Fermi ratings (Fig. 6A, βPD = 0.54, p = 5×10-19; βGE = 0.43, p = 0.01). In Study 2, the effect of the experimental manipulation (Fermi vs. Numbers instructions) on Fermi ratings is mediated by the increased presence of these markers in the conversations (Fig. 6B, total effect: 2.56±0.36, p=0.0006, direct effect: 1.86±0.37, p=0.0005, indirect effect of PD: 0.46±0.12, p=0.0004; indirect effect of GE: 0.25±0.11, p=0.01).

Taken together, these results indicate that conversations identified as exhibiting Fermi-style reasoning systematically contain the linguistic signatures predicted by the formal definition of the method. This provides converging evidence that the strategy implemented in the experiments reflects the combination of problem decomposition and rough approximation that characterizes the Fermi method.

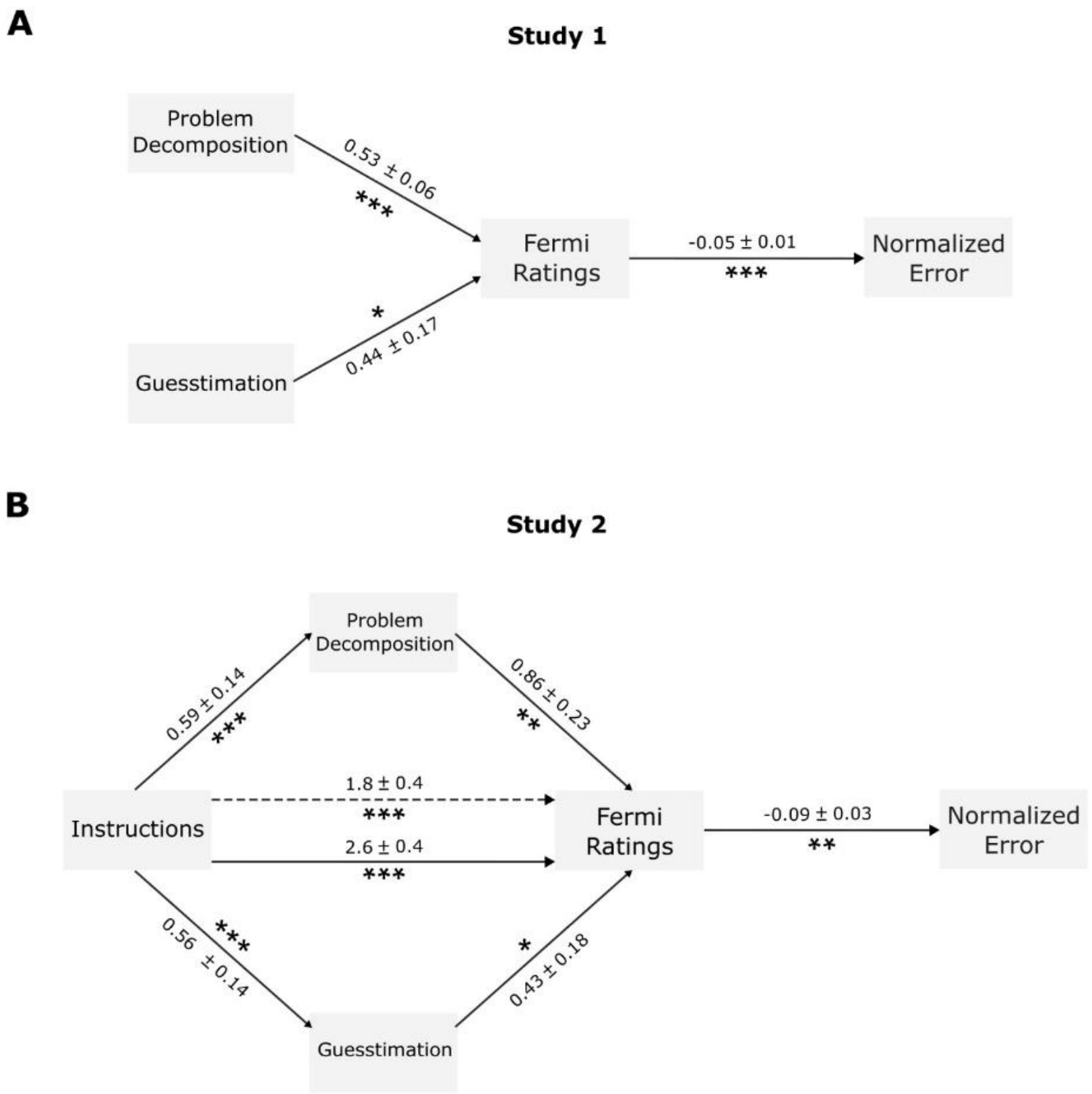

Fig. 5. Linguistic mechanisms underlying Fermi ratings. (A) *Linguistic markers of problem decomposition and guesstimation in Study 1, derived from the formal definition of the Fermi method, predict human-annotated Fermi ratings. In turn, higher Fermi ratings are associated with lower normalized estimation error. Coefficients correspond to regression estimates (± s.e.m.), and asterisks indicate statistical significance (*: p<.05, **: p<.01, ***: p<.001).* ***(B)*** *Mediation analysis for Study 2. Fermi instructions increase the presence of both decomposition and guesstimation markers in the conversations, which in turn predict higher Fermi ratings. Fermi ratings are negatively associated with normalized collective error. The dashed arrow indicates the direct effect of the experimental manipulation on Fermi ratings after accounting for the linguistic mediators.*

2.2. In sum, this is not a convincing measure of reasoning, let alone a specific reasoning process like PD. It can and should be significantly improved. The human annotation classifications (and their correlations with this measure) are unsatisfying. This is partly because the framing of the classification is loaded to begin with. Annotators are told to assume chats fall along the framings of the conditions (i.e., as either being focused on numbers or on PD), but there are certainly many other possible classifications here, such that these distinctions may not capture the underlying variation of interest. The authors report a robustness test that their annotation results hold if people compare chats as being either number focused or focused on reasoning generally, but this actually reinforces my concern – the fact that this rating is highly correlated with the rating focusing on PD suggests it's by no means capturing PD specifically, and reasoning is a catch-all-term for such a wide variety of things. As a result, I'm not clear on what the ratings of a dozen or so students using is capturing and how this relates to a clear mechanistic picture.

The reviewer raised an important point. We agree that, in principle, human annotation procedures may be influenced by the instructions provided to annotators. This limitation is not unique to the present work but applies broadly to much of the literature, where annotated datasets often rely on relatively small numbers of raters. Nonetheless, the reviewer raises a relevant concern regarding the possibility that our annotation instructions may have implicitly collapsed the classification of conversations into a "Numbers vs. Fermi" dimension. We therefore took this point very seriously and implemented two complementary approaches to address it.

First, as described in the response to point 2.1 above, we developed theory-driven linguistic indicators derived directly from the formal definition of the Fermi method introduced in the revised manuscript. Specifically, we constructed separate linguistic markers capturing the two components of Fermi estimation: problem decomposition and guesstimation. Using these indicators, we find that the frequency of these markers strongly predicts the Fermi ratings assigned by human annotators across studies (Figure 5). This result suggests that the annotations are systematically related to identifiable linguistic features corresponding to the theoretical definition of the Fermi strategy.

However, we recognize that this analysis alone does not fully address the reviewer's concern, because the annotation instructions themselves could still have biased raters toward interpreting conversations in a way that does not directly reflect the definition of Fermi reasoning. To address this possibility, we implemented a second approach designed to remove this potential source of bias.

Specifically, we generated AI-based Fermi ratings using large language models (LLMs). For each conversation, we provided the model with the theoretical definition of the Fermi method derived from the literature and asked it to rate the extent to which the reasoning procedure described in the conversation matched this definition on a 0–10 scale. To ensure that the results were not dependent on the idiosyncrasies of a particular model, we repeated this procedure using five different language models (gpt-4.1-mini, gpt-5-mini, gpt-5.2, claude-haiku-4.5, and claude-sonnet-4.6).

Across models, we observed two consistent patterns. First, the LLM-based ratings tend to be more moderate than the human ratings, with values closer to the midpoint of the scale (paired t-test: $t(419) > 5.4$, $p < 9 \times 10^{-8}$). Second, and more importantly, we observe very strong correlations between the AI-generated ratings and the mean ratings produced by the human annotators (Table S3, Pearson correlation: $r > 0.70$, $p < 7 \times 10^{-64}$).

	Humans	GPT 4.1 mini	GPT 5.1 mini	GPT 5.2	Claude Haiku 4.5
GPT 4.1 mini	0.80				
GPT 5.1 mini	0.70	0.85			
GPT 5.2	0.78	0.89	0.84		
Claude Haiku 4.5	0.84	0.86	0.81	0.86	
Claude Sonnet 4.6	0.83	0.88	0.80	0.86	0.86

Table S3: *Pearson correlation coefficient between Fermi ratings produced by five different large language models (LLMs). All comparisons are statistically significant with p<0.001.*

Moreover, all main results of the paper replicate when using the AI-based ratings in place of the human annotations (Table 1).

	Study 1			Study 2			Study 3		
	Error	PD	GE	Error	PD	GE	Error	PD	GE
GPT 4.1 mini	-0.20^{***}	0.38^{***}	0.13^{**}	-0.21^{*}	0.39^{***}	0.33^{***}	-0.24^{***}	0.48^{***}	0.13^{*}
GPT 5.1 mini	-0.22^{***}	0.39^{***}	0.19^{***}	-0.28^{**}	0.44^{***}	0.32^{***}	-0.30^{***}	0.45^{***}	0.21^{***}
GPT 5.2	-0.20^{***}	0.45^{***}	0.12^{*}	-0.27^{**}	0.45^{***}	0.30^{***}	-0.27^{***}	0.50^{***}	0.11^{*}
Claude Haiku 4.5	-0.25^{***}	0.35^{***}	0.12^{*}	-0.31^{***}	0.40^{***}	0.35^{**}	-0.31^{***}	0.35^{***}	0.15^{**}
Claude Sonnet 4.6	-0.22^{***}	0.39^{***}	0.15^{**}	-0.29^{**}	0.46^{***}	0.34^{***}	-0.32^{***}	0.30^{***}	0.05

Table 1. Replicating main results using LLM-based Fermi ratings. *For each of five large language models (rows), the table reports Pearson correlation coefficients between LLM-generated Fermi ratings and key variables across the three studies. Columns show correlations with normalized estimation error (Error), problem decomposition markers (PD), and guesstimation markers (GE). Asterisks denote statistical significance (*: p<.05, **: p<.01, ***: p<.001).*

In response to the reviewer's comment, we have added a new section, main table (Table 1), and supplementary table (Table S3) reporting these analyses in detail (page 27):

Can large language models detect Fermi reasoning?

Because human annotation procedures can, in principle, be influenced by the instructions provided to raters, we conducted an additional analysis to evaluate whether Fermi ratings could be reproduced using an independent procedure based solely on the formal definition of the method (presented in the Introduction). Specifically, we generated AI-based Fermi ratings using large language models (LLMs) (Bermejo et al., 2025). For each conversation, the model was provided with the theoretical definition of the Fermi method and asked to rate, on a 0–10 scale, the extent to which the procedure in the conversation matched that definition.

To ensure that the results were not dependent on the idiosyncrasies of a particular model, we repeated this procedure using five different LLMs (gpt-4.1-mini, gpt-5-mini, gpt-5.2, claude-haiku-4.5, and claude-sonnet-4.6). Across models, the resulting ratings show strong correlations with the mean human annotations (Pearson $r>0.7$, $p<7x10^{-64}$ for all comparisons, Table S3) as well as strong correlations among models themselves (Pearson $r>0.8$, $p<8x10^{-98}$ for all comparisons, Table S3), indicating a high level of agreement across independent rating procedures.

Importantly, the main empirical relationships reported in this paper replicate when using AI-based ratings in place of the human annotations. Across all three studies, higher Fermi ratings generated by the language models are associated with lower estimation error (Table 1). In addition, these ratings show the same expected relationships with the linguistic indicators introduced above: they are positively associated with both problem decomposition and guesstimation markers (Table 1).

These results provide converging evidence that the Fermi ratings capture a stable and identifiable reasoning pattern rather than an artefact of the human annotation procedure. Conversations rated as exhibiting stronger Fermi-style reasoning (whether by human raters or LLMs) systematically display the linguistic signatures predicted by the formal definition of the method and are associated with improved collective accuracy.

Finally, we would like to clarify a related aspect of the original submission. In one of our secondary analyses we asked annotators to rate conversations in terms of "reasoning" rather than explicitly in terms of the Fermi method. This choice was motivated by a long-standing distinction in the literature on collective intelligence between aggregation mechanisms (i.e., voting or sharing numbers) and deliberative mechanisms involving the exchange of reasons (e.g., Landemore & Page, 2014). Because the Fermi method is itself a reasoning procedure, we expected that conversations exhibiting stronger Fermi-style reasoning would also receive higher reasoning ratings. Consistent with this expectation, we observe a strong correlation between reasoning ratings and Fermi ratings (Pearson correlation: $r = 0.84$, $p = 2x10^{-115}$, see also Fig. S2).

Following the reviewer's comment, we now clarify the conceptual motivation behind this analysis in the revised manuscript (page 27):

Second, we conducted an additional annotation exercise designed to capture a broader form of deliberation. A separate group of six raters (5 female; mean age = 25.8 years, s.d. = 5.0) evaluated the conversations using more general instructions, rating the extent to which participants exchanged reasons to justify the group's consensus value. This instruction was motivated by the distinction commonly made in the collective intelligence literature between number-based aggregation and deliberation involving the exchange of reasons (e.g., Landemore & Page, 2014). Because the Fermi method is itself a reasoning procedure, we expected that conversations exhibiting stronger Fermi-style reasoning would also receive higher reasoning ratings.

Consistent with this expectation, these broader reasoning ratings were strongly correlated with the original "Fermi" ratings (Pearson $r = 0.84$, $p = 2{\times}10^{-115}$), and the main results reported in Fig. 2 remain unchanged under this alternative annotation scheme (Fig. S2). Importantly, these reasoning ratings were not intended as a mechanistic operationalization of the Fermi method itself, but to increase confidence in the robustness of the annotations: because Fermi-style estimation is one form of reason-based deliberation, similar results under this broader coding scheme indicate that the findings do not hinge on a narrowly framed annotation instruction.

Importantly, these reasoning ratings were not intended to provide a mechanistic operationalization of the Fermi method, but rather to situate our experimental manipulation within the broader literature contrasting number-based aggregation and reason-based deliberation. In the revised manuscript, the mechanistic analysis is now provided separately through the theory-driven linguistic markers which are now complemented by the LLM-based Fermi ratings described above.

Taken together, the revised manuscript now provides a clearer mechanistic picture of what the Fermi ratings capture. In particular, we show that (i) conversations receiving higher ratings exhibit the linguistic signatures predicted by the formal definition of the Fermi method, and (ii) these ratings can be independently reproduced using large language models that apply the same theoretical definition. We thank the reviewer for raising this concern, which led us to substantially strengthen the operationalization and validation of the central construct in the paper.

2.3. This concern was reinforced when I saw the figure 2. The example of PD given by the authors is a case where a participant breaks down the problem by talking about how many floors are in the building and estimating these components. Yet, in both of the examples shown in figure 2, someone mentions/inquiries into how many floors are in the building. The difference is that in the PD case, people focus on this and elaborate, whereas in the numbers case, they ignore this and focus on numbers. But some form of PD is happening in both cases (i.e., someone has shared an argument along these lines). It's not clear to me why the first case should be listed as PD = 1 whereas the latter as PD = 8 where in both cases one of the messages (out of only a dozen) mentions exactly the strategy described as PD. While I appreciate the correlations the authors provided to support the annotator judgments, this led me to wonder about variance. How much variance is there at the individual chat level, and what are the chats associated with the highest level of variance in people's use of this coding scheme? Or, in other words, if we look only at the chats in the by number condition, is there a correlation with the presence of PD and performance? It seems that clearly some of the chats in this condition have higher PD than others. As well, a valuable robustness test would be to test whether the main results hold when only comparing the number and PD ratings with the lowest variance cut off by some threshold (like quartile ranges etc.) to make sure the comparisons are really driven by the clear cut cases that capture the differences that are supposed to make a difference here.

The reviewer is correct that the two conversations illustrated in Figure 2 do not normatively imply specific Fermi ratings such as 1 or 8. In the revised manuscript (page 8) we now clarify that these examples are intended only as illustrative cases showing how raters might assign different scores depending on the extent to which problem decomposition and guesstimation are present in the conversation. They are not meant to imply that these particular values are uniquely correct.

Figure 2A illustrates two real examples: one conversation in which participants combined their initial estimates to reach consensus (left panel), and another in which participants decomposed the problem into intermediate quantities and proposed approximate values for those quantities (right panel). These examples illustrate how different conversational strategies can lead raters to assign different "Numbers" and "Fermi" scores. In particular, conversations dominated by the exchange and aggregation of initial estimates would typically receive higher "Numbers" ratings, whereas conversations involving problem decomposition and rough approximations would tend to receive higher "Fermi" ratings. These examples are intended only as illustrative cases showing how raters may interpret the conversational strategies present in the discussion, rather than implying that any specific numerical rating is uniquely correct for these conversations.

More broadly, the reviewer raises an important point regarding variance across annotators. In the original manuscript we focused primarily on the mean ratings across annotators and on the correlations between raters as a measure of consistency, but we had not explicitly examined the variance of ratings at the individual chat level. Following the reviewer's suggestion, we conducted additional analyses to address this issue.

First, we examined the typical magnitude of disagreement across annotators. We find that the standard deviation of Fermi ratings across raters at the individual chat level is relatively small (mean SD ≈ 0.19 Likert points in an 11-point scale), indicating that annotators generally produced highly consistent evaluations of the conversations (mean SD: 0.19, min SD: 0, max SD: 0.36, IQR: 0.11).

Second, we tested whether this between-rater variance was systematically related to any of the key variables in Study 2. We find that the variance of ratings at the chat level does not differ significantly across experimental conditions (Mann–Whitney U test: z = 1.8, p = 0.07; Cohen's d = 0.29), and does not correlate with the linguistic markers of Fermi reasoning introduced above (problem decomposition: r = 0.07, p = 0.36; guesstimation: r = 0.09, p = 0.22) or with the collective estimation error of the groups (r = 0.02, p = 0.78).

Finally, following the reviewer's suggestion, we conducted an additional robustness analysis focusing only on the clearest cases. Specifically, we repeated the main analyses after excluding conversations whose between-rater variance fell within the highest quartile of the distribution. The main results remain unchanged under this restriction (Pearson correlation between Fermi ratings and collective error: r = -0.08, p = 0.04), indicating that the observed relationship between Fermi ratings and estimation accuracy is not driven by conversations for which annotators showed greater disagreement.

Taken together, these analyses suggest that the reported effects are not driven by variability in how annotators interpreted the coding scheme. Moreover, in the revised manuscript we further address this concern by introducing LLM-based Fermi ratings derived directly from the theoretical definition of the method (see point 2.2 above), which replicate the main findings independently of human annotations. These additional analyses are now reported in the Methods section (page 36):

Evaluating variability between human raters

We studied if the relationship between Fermi ratings and collective accuracy could reflect variability in how annotators interpreted the coding scheme. To evaluate this possibility, we examined the variance of Fermi ratings across annotators at the level of individual conversations. Disagreement among raters was generally small (mean SD ≈ 0.16 Likert points), indicating high consistency in the evaluations. Moreover, between-rater variance did not differ significantly across experimental conditions (Mann–Whitney U test: z = 1.8, p = 0.07; Cohen's d = 0.29) and did not correlate with either the linguistic markers of Fermi reasoning or collective estimation error (problem decomposition: r = 0.07, p = 0.36; guesstimation: r = 0.09, p = 0.22; collective error: r = 0.02, p = 0.78). As an additional robustness check, we repeated the main analyses after excluding conversations whose between-rater variance fell within the highest quartile of the distribution. The results remain unchanged under this restriction (Pearson correlation between Fermi ratings and collective error: r = -0.08, p = 0.04), indicating that the observed relationship between Fermi ratings and estimation accuracy is not driven by conversations for which annotators showed greater disagreement.

I will say though that as much as these robustness tests and clarifications will be helpful, I don't think these alone will address the first order problem of collective fermi estimation being vaguely defined and operationalized in an unclear way with respect to the linguistic measures.

We appreciate the reviewer's concern. As detailed in the points above, the revised manuscript now directly addresses this issue by (i) introducing a formal definition of the Fermi method, grounded in the existing literature, and (ii) developing multiple independent operationalizations of this construct. In particular, we now show that conversations exhibiting higher Fermi ratings systematically contain the linguistic signatures predicted by this definition, and that the main results replicate using LLM-based ratings derived from the same theoretical definition, independently of the human annotations. Taken together, these additions substantially clarify both the conceptual definition and the empirical operationalization of collective Fermi estimation, and provide a clearer mechanistic link between the reasoning processes observed in the conversations and the improvements in collective estimation accuracy.

We are grateful to the reviewer for these comments, which have helped us substantially improve the clarity and robustness of the manuscript.

Reviewer #3:

This paper reports a series of 3 experiments to test whether problem decomposition ("fermi estimates") improve estimate accuracy on numeric trivia questions. Each experiment follows a similar protocol: (1) individuals answer 8 numeric trivia questions, (2) individuals discuss a random subset of 4 questions in groups of four, (3) individuals answer all 8 questions again. The primary focus of interest is the extent to which individuals decompose the numeric estimates into sub-estimates to be recombined into a final answer.

The first experiment is exploratory (not pre-registered) and analyzes endogenous variation in decomposition by rating each conversation on the extent to which decomposition occurs. The second experiment is a pre-registered confirmatory analysis in which the experimenters randomly allocate groups to either a condition which explicitly instructs them to decompose the estimates, or a control condition.

Besides my comments below, this study reports clear, strong results with replicable and straightforward methods. Study 3 in particular provides a clean comparison of independent individuals versus collaborating groups. If the authors can clarify my concern about the possibility of looking answers up online, it will represent a very strong contribution. Were it not for that (presumably easily addressed) concern, I would say this paper could be published as-is. Nonetheless, I offer a number of major and minor comments aimed at improving the manuscript, in no particular order.

We appreciate the reviewer's careful reading of the manuscript and the positive assessment of the contribution of the study. We are particularly glad that the reviewer found the experimental design and the results clear and replicable. The reviewer raises a number of thoughtful questions and suggestions aimed at improving the manuscript, which we found very helpful.

In the revised version, we have carefully addressed each of the points raised below. In particular, we clarify the issue regarding the possibility of participants looking up answers online and provide additional details where needed to strengthen the transparency of the experimental protocol. We believe that these revisions improve the clarity and robustness of the manuscript.

Major Comments

1- Generalizability. I realise that you don't have data to address the question of "when will fermi estimates improve outcomes" but perhaps you can offer some brief discussion on this point. Presumably, you picked questions for their decomposibility—perhaps you could tell us how you chose the questions.

This is an important point. In our experiments we followed the standard paradigm used in the wisdom-of-crowds literature, which typically relies on numerical estimation problems involving positive quantities. This design allows performance to be evaluated objectively and facilitates comparison with prior work on collective estimation.

Consistent with the reviewer's intuition, the questions were selected so that they could plausibly be decomposed into intermediate components (e.g., estimating sub-quantities that can be combined to produce the final answer). However, we agree that this task structure represents only a subset of problems in real-world settings.

Following the reviewer's suggestion, we have added a paragraph in the Discussion clarifying this point. In particular, we now acknowledge as a limitation that the present study focuses on a relatively narrow class of estimation problems and discuss how decomposition-based reasoning might generalize to other domains. We suggest that similar reasoning processes may apply in settings where complex judgments can be broken down into simpler sub-questions, even when the final outcome is not a numerical estimate. We also highlight this as an important direction for future research.

This paragraph now appears on page 30:

However, several limitations remain. As with much of the crowd wisdom literature, it is unclear whether our findings extend to domains where correct answers are categorical rather than continuous (for a notable exception, see Becker et al., (2022)). Moreover, many real-world group decisions involve not only categorical judgments, but also explanations, advice, planning, or the analysis of complex situations, for which numerical aggregation procedures do not directly apply. Our findings therefore speak most directly to a relatively narrow class of estimation problems. However, the mechanism we study (i.e., collective decomposition of a complex problem into simpler components) may generalize beyond numerical estimation. In principle, many complex judgments can be structured as a set of simpler sub-questions whose answers can then be integrated into a final decision. Future work should examine whether collective Fermi-style reasoning can provide similar benefits in domains where the outcome is not a numerical estimate but rather a categorical judgment, explanation, recommendation, or plan of action.

2- Mechanisms. Your deliberation condition confounds collective deliberation with individual deliberation, i.e., you can't tell whether the improved accuracy from group interaction is due to the group interaction per se, or the fact that individuals are improving of their own accord. Although you randomize groups to discuss 4 questions and leave 4 questions undiscussed (a nice design) this still yields huge differences in attention-per-question for discussed and undiscussed. On average, each person can give attention worth about 75 seconds to each undiscussed question (5+5=10 minutes, divided by 8 questions) versus about 150 seconds for discussed questions (75 seconds + 5 minutes divided by 4 questions). And so, your primary design conflates interaction between people with individual attention. As your primary focus is the comparison of fermi with non-Fermi conditions I don't see this as a fatal flaw of the paper, but you should be more circumscribed about your results. Your results indeed replicate prior findings that deliberation in small groups does not HARM the wisdom of crowds, but you can't say that deliberation groups yield more accurate answers than people working alone, for a comparable amount of time. The only place you really do get this comparison is in the very last result reported, comparing Figs 5C and 5D—which clearly shows that people interacting with other people produces more improvement than people working alone.

The reviewer is right: In Studies 1 and 2 the comparison between discussed and undiscussed questions may reflect not only the effect of collective interaction, but also differences in the amount of attention devoted to each question. Following the reviewer's suggestion, we have revised the manuscript to tone down the interpretation of these comparisons throughout the text, making it clearer that the results from these studies do not isolate the effect of group interaction from differences in individual attention. We also added a paragraph in the Discussion explicitly acknowledging this design feature.

At the same time, as the reviewer notes, this limitation does not critically affect the central comparisons of the paper. In Study 1, the key analysis concerns variation in how groups reasoned about the deliberated questions, rather than comparisons between deliberated and undeliberated questions. In Study 2, the main comparison is between two different instructions applied to deliberating groups. Finally, Study 3 provides a cleaner test of the reviewer's concern by comparing individuals who deliberate alone with groups who deliberate together, while holding the opportunity for deliberation constant, as well as the amount of individual attention spent on each question. As the reviewer correctly notes, this comparison shows that interaction with others produces larger improvements than individual reflection alone.

We have now clarified these points in the revised manuscript (pages 30-31):

Another limitation of the present design is that, in Studies 1 and 2, questions that were deliberated differed from those that were not deliberated not only because they involved group interaction, but also because participants could devote more attention to them. Because deliberated questions were discussed collectively for up to five minutes, whereas undeliberated questions were answered individually within the overall task time, the two types of questions may differ in the amount of individual attention allocated to them. As a result, comparisons between deliberated and undeliberated questions in those studies (which we present only in Fig. 1D) cannot cleanly isolate the effect of group interaction from differences in attention per question. Importantly, however, the central comparisons of the present work do not rely on this contrast. In Study 1, the key analyses focus on variation in how groups reason about deliberated questions. In Study 2, the main comparison is between two instruction conditions applied to deliberating groups. Finally, Study 3 provides a cleaner test of the role of social interaction by comparing individuals deliberating alone with groups deliberating together, holding the opportunity for deliberation constant. Together, these analyses suggest that while differences in attention may contribute to some contrasts, the central findings regarding collective Fermi-style reasoning are not driven by this feature of the design..

3- Exogeneous improvements. Please tell us more about the setting in which participants conducted the study, and what information you have about their ability to look up answers to these questions online. Notably, these questions are particularly easy to find on google—the number of episodes of 'Friends' is a fact about one of the most popular television shows of all time. If they were able to search for answers, this possibility interacts with group deliberation in ways that are uninteresting: a group collaborating can divide-and-conquer (i.e. assign different questions to search) whereas a collection of independent individuals faces some redundancy. E.g., was this study conducted in a physical lab setting that prevented participants from searching online for answers?

We took this concern very seriously. Participants were recruited from the Subject Database of Universidad Torcuato Di Tella, which we now clarify in the manuscript to improve transparency about the study population. The experiment was conducted online rather than in a physical laboratory setting, meaning that participants were not technically prevented from looking up answers during the task. While the instructions explicitly discouraged searching for answers online, we agree that this possibility represents a potential concern.

At the same time, several features of the experimental design make systematic "divide-and-conquer" searching effectively impossible. Participants did not know in advance which questions would later be discussed collectively, nor with whom they would eventually be assigned to a group, and the questions were presented sequentially during the task. As a result, participants could not coordinate in

advance to divide the burden of searching across group members for the subset of questions that would later be deliberated.

Empirically, we also find no substantial evidence for looked-up answers. If participants had systematically searched for the correct answers online, we would expect conversations to frequently produce the exact correct value for the question. In practice, only a small fraction of conversations produced answers that matched the true value exactly (between 0.6% and 2.8% across studies). Importantly, this level is consistent with what could plausibly occur without looking up for the answer, especially for some questions whose true values fall within commonly guessed ranges (e.g., How many kilograms of ice cream does a person in Argentina eat per year? Answer: 7).

To address this concern conservatively, we repeated our analyses after excluding all conversations that produced the exact correct answer, and found that the main results remain unchanged (e.g., Mann–Whitney U test between "Fermi" and "Numbers" conditions in Study 2: z = 1.95, p = 0.048; Cohen's d = 0.25). Following the reviewer's suggestion, we now report these robustness analyses in the Methods section (page 35):

Robustness checks against answer look-up

All studies were conducted online. Although participants were instructed not to search for answers during the task, the online setting does not technically prevent such behavior. We therefore conducted additional robustness checks to evaluate whether the results could be driven by participants looking up the correct answers.

First, we examined how often group conversations produced the exact true value of the target quantity. If participants had systematically searched for the answers online, we would expect exact matches to occur frequently. In practice, only a small fraction of conversations produced answers that matched the true value exactly (between 0.6% and 2.8% across studies). For some questions, such matches could plausibly without looking up the answer because the correct value falls within commonly guessed ranges (e.g., "How many kilograms of ice cream does a person in Argentina eat per year?"). Therefore, to address this concern conservatively, we repeated the main analyses after excluding all conversations that produced the exact correct answer. The results remain qualitatively unchanged (e.g., Mann–Whitney U test between "Fermi" and "Numbers" conditions in Study 2: z = 1.95, p = 0.04; Cohen's d = 0.25).

4- Title. Although it's not a breaking issue, I take some issue with the title. You don't show that collective problem decomposition "drives" the wisdom of deliberative crowds, you show that it improves the wisdom of crowds. You also show that collaborative decomposition is more effective than individual decomposition. Clearly, it is true also both that the wisdom of crowds still occurs without decomposition and that decomposition improves outcomes for individuals working alone (5D).

We agree with the reviewer that the original title overstated the strength of the claim. Following this suggestion, we have revised the title from "Collective problem decomposition drives the wisdom of deliberative crowds" to "Collective problem decomposition improves the wisdom of deliberative crowds." This wording more accurately reflects our results, which show that decomposition-based reasoning improves the accuracy of collective estimates rather than being a necessary condition for the wisdom of crowds.

Minor Comments

5- p.18, you don't report statistical significance or effect size for the improvements that result from individual/independent fermi decomposition.

We now report the corresponding effect sizes and statistical significance for the improvements resulting from individual/independent Fermi decomposition. These statistics have been added to the Results section (page 24).Thanks.

6- Some revision of terminology might help. I recognize these terms are used ambiguously in lay language, so you'll need to figure out a consistent but clear parlance. You use the term "deliberation" to refer to group discussion, which is confusing in this case because you also allow for individual deliberation i.e. careful thought. You use the term "discussed" to refer to individual deliberation (e.g. p.18) which is just confusing. I realize that the individuals are "discussing" with the empty chat interface but it's not really quite the same, I think. Perhaps you can find words that are explicitly group-restrictive to refer to peer-to-peer discussion, and explicitly intelligence-restrictive to refer to careful thinking.

This is a helpful suggestion. As the reviewer notes, the original terminology could be confusing because the manuscript used "discussed" in contexts where participants were not interacting with others. We have revised the terminology throughout the manuscript to clarify this distinction. In particular, we now use "deliberation" to refer to reasoning about a question (which can occur individually or collectively), while reserving terms such as "collective deliberation" when referring specifically to peer-to-peer interaction among participants. We have also replaced instances of "discussed" in the individual condition of Study 3 with terminology that more accurately reflects individual reasoning in the chat interface.

These changes improve the consistency and clarity of the terminology used throughout the manuscript and we thank the reviewer for this point.

7- For study 1, please explicitly state your prior for the Bayesian analysis, so we can follow your analysis without downloading the code. For study 3, you refer us to the methods for explanation of the generalized linear effects model, but then we have to look online to find out the model. If word limit allows, please give us basic details of the analysis right in the paper.

We have now clarified these points in the manuscript. In Methods (page 38), we now explicitly report the prior used in the Bayesian analysis so that the analysis can be fully understood without consulting the code.

Bayes factor analyses for equivalence testing

To assess evidence in favor of the absence of effects in several analyses, we complemented frequentist tests with Bayes factor analyses for equivalence testing. Bayes factors were computed using the default prior specification for standardized effect sizes proposed by Rouder et al. (2012). Specifically, we used a Cauchy prior distribution over standardized effect sizes centered at zero, following standard procedures in the Bayesian analysis of experimental designs. This default prior has desirable properties such as scale invariance and has become widely adopted in psychological research for evaluating evidence in favor of the null hypothesis.

For Study 3, we now provide the equation underlying the basic specification of the generalized linear effects model directly in the Methods section (page 39):

The generalized linear model was:

$$\Delta E_{ij} = \beta_0 + \beta_1 \, deliberated_{ij} + \mu_j + \varepsilon_{ij} \qquad [2]$$

where ΔE_{ij} denotes the change in error for observation i associated with question j and $deliberated_{ij}$ is a binary predictor indicating whether the question was deliberated. The model includes a fixed intercept and fixed effect for deliberation, as well as a random intercept μ_j to account for repeated observations across questions. The same type of generalized linear mixed-effects models were used in Study 3 (Fig. 5C and Fig. 5D) with a dummy coding for the collective condition.

These additions improve the transparency and reproducibility of the analysis. Thanks.

8- There are some minor deviations from the pre-registration. I don't see these as problematic (e.g. swapping a non-parametric test for a parametric test) but it is worth explaining why you made these decisions. If I'm interpreting correctly, it looks like you switched from reporting Stage i2/stage 3 error as DV, to change-in-error as a DV, which is a more powerful test. Please report all pre-registered analyses or deviations therefrom, with explanation. You also introduce some additional analyses—please label non-pre-registered analyses as "exploratory."

This is a helpful observation. The reviewer is correct that in Study 3 the main analyses reported in the manuscript focus on change in error between stages rather than directly analyzing the stage-i2 error as specified in the pre-registration.

Our motivation for reporting change-in-error was that this metric more directly captures the improvement produced by deliberation relative to each participant's initial estimate. However, we agree with the reviewer that the manuscript should clearly report the pre-registered analyses as specified and transparently distinguish them from additional analyses.

Following the reviewer's suggestion, we have revised the manuscript to:

i) Report the pre-registered analyses explicitly, including the mixed-effects regression with stage-i2 error as the dependent variable and the Wilcoxon rank-sum test comparing errors across conditions.

ii) Clearly label analyses based on change in error as exploratory analyses, introduced to provide a more interpretable measure of improvement.

Importantly, the results of the pre-registered analyses lead to the same qualitative conclusions as the change-in-error analyses reported in the original manuscript. These modifications to the original manuscript now appear on pages 23-24:

This design allowed us to directly compare the effectiveness of applying the Fermi method individually versus in groups as a strategy for improving accuracy (Figs. 5C and 5D). Following our pre-registration, we first tested whether final individual error at stage i2 differed between the Collective and Individual conditions. A mixed-effects linear regression with normalized individual error as the dependent variable, a dummy variable coding for the Collective condition, and random intercepts for the eight questions showed that participants in the Collective condition produced significantly lower errors at stage

i2 than those in the Individual condition (β = -0.24±0.07, p = 0.0007). A Wilcoxon rank-sum test comparing the distributions of individual errors across conditions yielded the same conclusion (z = 3.12, p = 0.002, Cohen's d = 0.26).

To facilitate interpretation of how deliberation affected performance relative to each participant's initial estimate, we also report an exploratory analysis examining the reduction in error between the initial individual estimates (stage i1) and the final individual estimates (stage i2). First, we focused on the collective condition (Fig. 5C). We observed that, while participants showed a modest improvement for non-deliberated questions (Cohen's d = 0.08), the improvement was substantially greater for collectively deliberated questions (Cohen's d = 0.69, β = −0.45 [−0.59, −0.31], SE = 0.07, $t(469)$ = −6.36, p = 5×10-10, R^2 = 0.10).

We then examined the individual deliberation condition (Fig. 5D), where participants worked alone but were instructed to apply the Fermi method. As in the collective condition, participants showed a modest improvement for non-deliberated questions (Cohen's d = 0.06) and a larger improvement for the ones that underwent individual deliberation (Cohen's d = 0.28, β = −0.17 [−0.28, −0.07], SE = 0.05, $t(469)$ = −3.28, p = 0.001, R^2 = 0.02). This suggests that applying the Fermi method individually may result in greater accuracy.

To formally compare conditions, we examined the reduction in error in the collective and individual conditions separately for deliberated and undeliberated items. For deliberated items, error reduction was substantially larger in the collective condition than in the individual condition (Mann–Whitney U test: z = 6.3, p = 3×10^{-10}; Cohen's d = 0.58). No such difference was observed for undeliberated items (Mann–Whitney U test: z = 1.0, p = 0.31; Cohen's d = 0.05). These results suggest that, although the Fermi method improves accuracy when applied individually, collective deliberation provides an additional benefit beyond individual reasoning alone.

We also carefully revisited the pre-registrations for Study 2 and did not identify deviations from the pre-registered statistical tests. However, in response to the reviewer's suggestion, we now explicitly distinguish pre-registered analyses from exploratory analyses throughout the manuscript to improve transparency.

These clarifications are now reported on pages 17-19. We thank the reviewer for asking us to clarify this point, which improves the transparency of the statistical analysis.

9- When you report confidence intervals, please indicate that they are (presumably) 95% intervals.

We now explicitly indicate throughout the manuscript that all reported confidence intervals are 95% confidence intervals. Thanks.

Reviewer #1

I already thought the ms. was strong, but the Authors' revisions have strengthened it further, so no notes, well done!

Response to Reviewer #1

Thank you for your kind assessment and for your support of the manuscript across both rounds. We are very happy that you found the revisions to have further strengthened the paper.

Reviewer #2 (Remarks to the Author):

The authors have taken my concerns seriously and have addressed them through significant revisions that have considerably improved the paper. I commend the authors for their hard work and careful efforts. I am satisfied with the changes, and I view this as a valuable contribution to the collective intelligence literature.

-Douglas Guilbeault

Response to Reviewer #2

We are particularly grateful for the depth and rigour of your engagement with our work. Your concerns in the first round were instrumental in shaping the most substantial revisions of the paper, including the introduction of the AI-based Fermi ratings and the additional robustness analyses. We are very glad that you find the revised manuscript a valuable contribution to the collective intelligence literature.

Reviewer #3 (Remarks to the Author):

The authors have satisfactorily addressed all of my comments.

I also considered the comments shared by Reviewer 2, which were thorough and had not occurred to me. I think Reviewer 2 is correct that we can't be totally sure about mechanisms, and that alternate explanations remain.

This possibility is not a huge concern for me---it is a feature of nearly any causal analysis. Moreover, the evidence provide makes clear that interventions instructing people to use decomposiiton help, and some evidence they're doing that---I think we can punt to future research to verify exactly what mechanism is between the treatment and the outcome. The process and intervention studied here are key to numeric estimation, but have not really been studied in the crowd/group context so it's natural that we will have to settle for incomplete insight.

Response to Reviewer #3

Thank you for your supportive evaluation. We fully agree that, as in any causal study of this kind, the exact mechanism connecting the treatment to the outcome cannot be settled with the present evidence alone. We hope that the formal definition of Fermi estimation, the linguistic indicators, and the AI-based ratings we have now included in the paper provide a useful foundation for future work that pins down the cognitive processes more directly. We appreciate the thoughtfulness with which you framed this point.
